# SPOROS: A pipeline to analyze DISE/6mer seed toxicity

**DOI:** 10.1371/journal.pcbi.1010022

**Published:** 2022-03-31

**Authors:** Elizabeth T. Bartom, Masha Kocherginsky, Bidur Paudel, Aparajitha Vaidyanathan, Ashley Haluck-Kangas, Monal Patel, Kaitlyn L. O’Shea, Andrea E. Murmann, Marcus E. Peter

**Affiliations:** 1 Department of Biochemistry and Molecular Genetics, Feinberg School of Medicine, Northwestern University, Chicago, Illinois, United States of America; 2 Department of Preventive Medicine/Division of Biostatistics, Feinberg School of Medicine, Northwestern University, Chicago, Illinois, United States of America; 3 Department of Medicine/Division Hematology/Oncology, Feinberg School of Medicine, Northwestern University, Chicago, Illinois, United States of America; University of Toronto, CANADA

## Abstract

microRNAs (miRNAs) are (18-22nt long) noncoding short (s)RNAs that suppress gene expression by targeting the 3’ untranslated region of target mRNAs. This occurs through the seed sequence located in position 2-7/8 of the miRNA guide strand, once it is loaded into the RNA induced silencing complex (RISC). G-rich 6mer seed sequences can kill cells by targeting C-rich 6mer seed matches located in genes that are critical for cell survival. This results in induction of Death Induced by Survival gene Elimination (DISE), through a mechanism we have called 6mer seed toxicity. miRNAs are often quantified in cells by aligning the reads from small (sm)RNA sequencing to the genome. However, the analysis of any smRNA Seq data set for predicted 6mer seed toxicity requires an alternative workflow, solely based on the exact position 2–7 of any short (s)RNA that can enter the RISC. Therefore, we developed SPOROS, a semi-automated pipeline that produces multiple useful outputs to predict and compare 6mer seed toxicity of cellular sRNAs, regardless of their nature, between different samples. We provide two examples to illustrate the capabilities of SPOROS: Example one involves the analysis of RISC-bound sRNAs in a cancer cell line (either wild-type or two mutant lines unable to produce most miRNAs). Example two is based on a publicly available smRNA Seq data set from postmortem brains (either from normal or Alzheimer’s patients). Our methods (found at https://github.com/ebartom/SPOROS and at Code Ocean: https://doi.org/10.24433/CO.1732496.v1) are designed to be used to analyze a variety of smRNA Seq data in various normal and disease settings.

## Author summary

We recently discovered a kill code embedded in the genome with powerful anti-cancer activity. It is based on only 6 nucleotides (comprised of A, G, C, or U) that when present in the sequence of a small double stranded RNA allows it to act like a microRNA (miRNA). miRNAs are important regulators of many cell functions. The ~2,300 known miRNAs in the human genome function through their business end, the seed sequence. When this seed sequence is 6 nucleotides long (6mer seed) and is comprised of mostly Gs, then these small RNAs can kill all cancer cells. Hence, this code is found in a number of miRNAs that have anti-cancer activities. However, the code is not limited to miRNAs and may also affect normal tissue under certain conditions. We have now developed SPOROS, a semi-automated bioinformatics pipeline that allows one to analyze any data set of sequenced small RNAs with a focus on their 6mer seed content and their potential to kill cells. We present two examples of such an analysis: the first example is a data set we generated on the expression of all small RNAs in a human colon cancer cell line compared to matching mutant cell lines that cannot produce most miRNAs; the second example is a publicly available data set of small RNAs isolated from normal brains and from brains of patients with Alzheimer’s disease.

This is a *PLOS Computational Biology* Methods paper.

## Introduction

micro(mi)RNAs are short (18-22nt long) noncoding RNAs that negatively regulate gene expression [1]. They are generated as double stranded (ds)RNA duplexes. Their activity involves only a very short region of complete complementarity between the ‘seed’, at position 2-7/8 of the guide strand of the miRNA [2,3] and ’seed matches’ predominantly located in the 3’ untranslated region (3’ UTR) of targeted mRNAs [4,5]. This targeting results in gene silencing [6]. miRNA biogenesis begins in the nucleus with the transcription of a primary miRNA precursor [7]. The Drosha/DGCR8 microprocessor complex first processes them into pre-miRNAs [8], which are then exported by Exportin-5 from the nucleus to the cytoplasm [9]. Once in the cytoplasm, Dicer/TRBP processes the pre-miRNAs further [10,11], and these mature dsRNA duplexes are then loaded onto argonaute (Ago) proteins forming the RNA-induced silencing complex (RISC) [12]. The active miRNA guide strand incorporates into the RISC [12], while the inactive passenger strand is degraded [13].

We previously discovered a powerful new cell death mechanism (6mer seed toxicity) that is based on a 6mer seed embedded in miRNAs. Any si-, sh, or miRNA that carries a 6mer seed of a certain nucleotide composition, can kill cancer cells by targeting the mRNAs of hundreds of genes that are critical for cell survival [14,15]. An arrayed high-throughput screen of all 4096 possible 6mer seeds in a neutral siRNA backbone with a chemically inactivated passenger strand in three human and three mouse cell lines revealed that the most toxic seeds were G-rich followed by seeds rich in Cs [16,17]. A consensus seed among the 100 most toxic seeds for human cells was identified as GGGGGC and we verified that it is toxic by targeting GCCCCC seed matches present in the 3’ UTR of numerous survival genes [17].

The number of putative human miRNAs has been estimated to be >2,300 [18]. The most widely established approach to study the role of miRNAs focuses on only the miRNAs that are significantly deregulated when comparing two states (e.g., tumor versus normal tissue, or two developmental stages of an embryo). Hence, most methods to normalize and analyze miRNAs are aimed at allowing investigators to identify deregulated individual miRNAs or groups of miRNAs. This makes the depiction of the relevant miRNAs more manageable as there is no need to visually display hundreds of miRNAs at the same time. However, there are two major drawbacks to this approach: First, the detected fold change in relative expression of a deregulated miRNA does not allow one to conclude that a miRNA is significantly expressed, and second, miRNAs that belong to different families but function in similar ways in different tissues or in a disease context in different patients, are hard to identify.

The 6mer seed toxicity concept requires analysis of short (s)RNAs, including miRNAs, in a different way. Rather than aligning all reads to the genome and finding the ones coding for miRNA genes, the only relevant information needed of any sRNA that is bound to the RISC and active in RNA interference (RNAi) function, is the precise knowledge of its 6mer seed (position 2–7 from the 5’ end). The nature of the sRNA initially is secondary when analyzing sRNAs that are bound to the RISC, but total small RNA Seq data can also be useful, as long as the reads are in the range of 18 and 25 nt long. It has become clear that this activity is not only found in miRNAs, but in any abundant sRNA such as tRNA or ribosomal (r)RNA fragments that can be loaded into the RISC and exert RNAi [19,20]. We now describe SPOROS (Greek for seed), a semi-automated pipeline that allows for the analysis of sRNAs by focusing not on individual miRNAs and their targets, but on the 6mer seed of any sRNA that is loaded into the RISC. SPOROS generates multiple output files that allow one to assess both composition and predicted seed toxicity changes in miRNAs in any small (sm)RNA Seq data set. We present two sample analyses to illustrate the power and utility of the pipeline. The first example is a smRNA Seq dataset of RISC-bound sRNAs in a wild-type (wt) human cancer cell line and two mutant cell lines lacking expression of either Drosha or Dicer, resulting in a fundamental reduction in miRNA expression. The second example is based on a publicly available small RNA Seq data set derived from postmortem normal and Alzheimer’s disease (AD) patient brains. The first example starts with raw sequence reads, while the second starts with a count table of reads by samples; SPOROS can be run either way. The focus in developing SPOROS was to provide simple and robust analysis tools that can be used without requiring advanced programming knowledge.

## Methods and data sets

### Example data sets

The first smRNA Seq data set of RISC-bound sRNAs was generated by performing an Ago pull down experiment followed by smRNA Seq as described before [16]. In brief, 10^6^ HCT116 wt, Drosha knock-out (k.o.) or Dicer k.o. cells [21] were subjected to an Ago pull down (in duplicate) using a bead bound GW182 protein [22]. After smRNA library preparation, the samples were subjected to 50 nt single end smRNA Seq on an Illumina HiSEQ4000 (accession number GSE182222). In this case, the raw fastq files serve as input for SPOROS. The second smRNA Seq data set was obtained from GEO (accession number GSE63501) and contains sRNAs (16–25 nt in length) derived from 7 control and 6 AD brains, and 3 brains from patients with severe primary age-related tauopathy, termed tangle-predominant dementia (TPD) [23]. It was reported that AD and TPD brains had a downregulation of the highly conserved brain miRNA miR-219. In this example, the sample-specific read counts are compiled into a table which is used as input for SPOROS. While compilation of the table is not part of the SPOROS pipeline, scripts used for this purpose are made publicly available within both Github and Code Ocean for maximum transparency.

### The SPOROS pipeline

**Fig 1:**
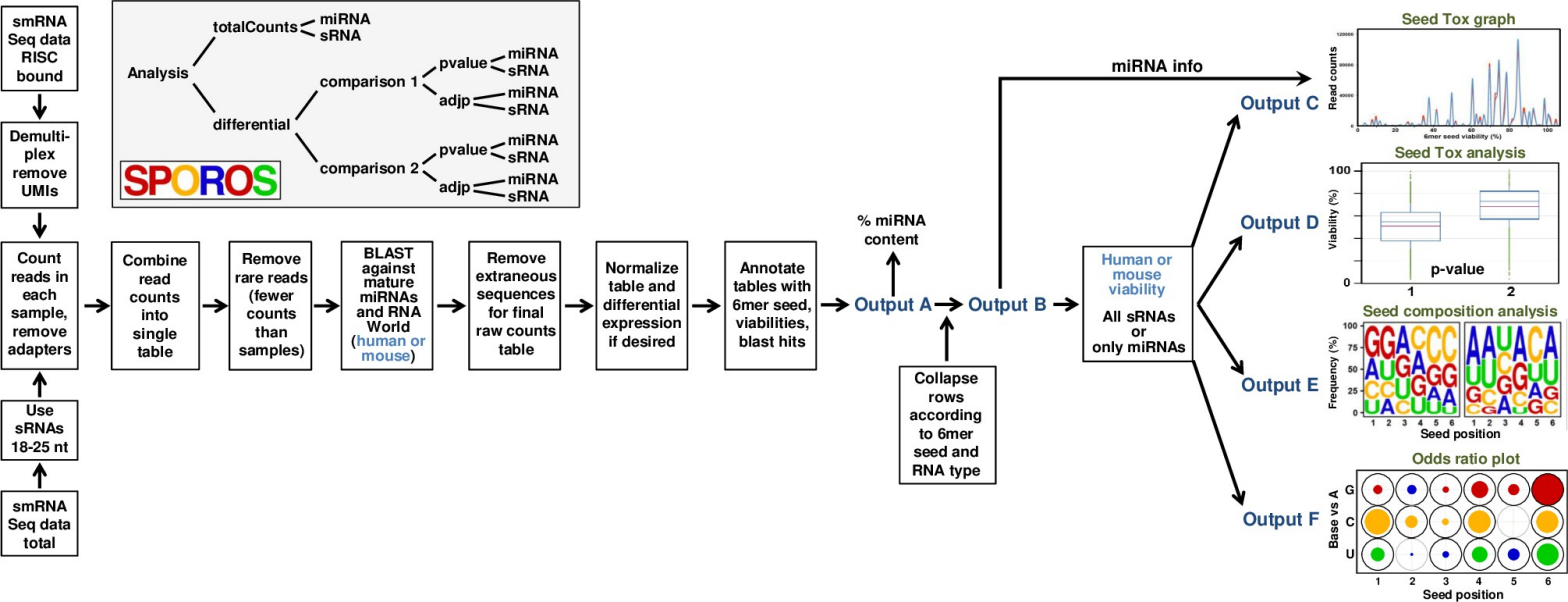
SPOROS workflow developed to analyze seed toxicity of smRNA Seq data. From left to right: smRNA seq data, either total or RISC-bound, are trimmed and cleaned and then compiled into a counts table. Rare reads are removed (fewer counts than the number of samples) and the remaining reads are BLASTed against all mature miRNAs or RNA world data sets of all small RNAs (either human or mouse). Reads that hit artificial sequences in the RNA world datasets are again removed. The remaining raw counts table can be normalized to 1 million reads per sample or column or used for differential expression analysis. RawCounts, normCounts, or differential tables are annotated with 6mer seed, 6mer seed viability, miRNA, RNA world to generate Output A tables. At this point the miRNA content (%) can be determined. Reads of this Output A file are collapsed according to 6mer seed and RNA type resulting in Output B. At this point all short RNAs can be analyzed (sRNA) or just the miRNA fraction. Output B is fed into four scripts generating four output files: C: A Seed Tox graph that depicts all miRNAs as peaks according to their seed viability; D: Average predicted 6mer seed toxicity of all reads in a samples depicted as box and whisker plot; E: Weblogo plot showing the average seed composition in positions 1–6 of the 6mer seed in each sample; F: The result of a multinomial mixed model odds ratio analysis allowing to compare both different 6mer seeds as well as differences in each position of different seeds. The hierarchy of folders and subfolders generated by SPOROS is shown in a grey box.

The goal of the SPOROS pipeline is to display and analyze abundance of sRNAs according to their predicted 6mer seed toxicity (**Fig** 1). It can be accessed at https://github.com/ebartom/SPOROS and as an executable Code Ocean capsule at https://doi.org/10.24433/CO.1732496.v1. At its heart, SPOROS is a Perl-based decision tree. Given a few essential arguments (location and type of the input data, organism, experimental parameters if relevant), SPOROS will generate a commented shell script listing each step in the pipeline. This script can be run on the command line to carry out the pipeline in its entirety, and all of the scripts and necessary dependencies have been set up within the Code Ocean capsule for ease of use. The code and resource files can also be downloaded from Github or Code Ocean and installed within any Unix system. The two examples are both from human samples, but support for mouse samples is also fully implemented. Any smRNA Seq data set (either total or bound to the RISC) can be used, either starting from raw fastq files, or from a table of read counts. While the 6mer seed toxicity concept is based mostly on the activity of miRNAs, it can be applied to any sRNA that enters the RISC as a guide, and hence the relevant activity of an sRNA is determined by its position 2–7 (the 6mer seed). This allows one to display all sRNAs in a graph as a function of only the predicted toxicity of its 6mer seed. While the 3’ end of the RNAs is not that relevant for their activity, for this analysis to succeed the knowledge of the exact 5’ start is critical. Consequently, the first step of the analysis is to de-multiplex the samples and remove any Unique Molecular Identifier (UMI) and adapter sequences. Trim_galore is used to identify and remove standard Illumina adapters. 5’ adapter sequences are removed first, followed by the removal of the 3’ adapters which in the case of our libraries is the substring TCCGACGATC. The adapter and primary sequences are based on our library prep for Ago pulldown libraries. When analyzing RISC bound sRNAs all reads >6 nts in length are being analyzed. The vast majority of these reads will be in the range of 18–25 nt and we did not find any RISC bound miRNA derived reads shorter than 18nt. When analyzing total smRNA Seq data, all reads that are longer than 25 or shorter than 18 nt are removed, as shorter and longer reads have a reduced chance of entering the RISC. Once any extraneous sequences are removed, a table of all unique reads observed in all samples, and their counts in each sample is generated. To reduce the files size at this point and remove most reads that are likely the result of sequencing errors, all reads with a normCount of less than n (n being the number of samples in an analysis) are deleted. An intermediary table containing all reads is also generated, and if the user prefers not to use such a threshold, this table can be used as input for SPOROS. In general, if demultiplexing and adapter sequences vary significantly, or if the desire is to keep all reads, no matter how rare, SPOROS can be started from a counts table.

At this point, all of the reads corresponding to rows in the count table are annotated using BLAST. More specifically, a BLAST search is performed against a list of small RNAs (data sets for human and mouse were obtained from Dr. Thomas Tuschl, and can be found in the Code Ocean capsule: https://doi.org/10.24433/CO.1732496.v1). We allowed a 95% identity for the search. To further refine the read list, any read that contains the following words in this Blast assignment is removed: "Tuschl", "artifical", "marker", "adapter", "artificial". Reads are also annotated with miRNA information by blasting each read against a curated list of either human or mouse mature miRNAs (lists are in **S1** and **S2 Tables**). For this analysis, we set the stringency so that only hits with at least 18 nt of complete identity between the queried read and the mature miRNA are counted.

**Fig 2:**
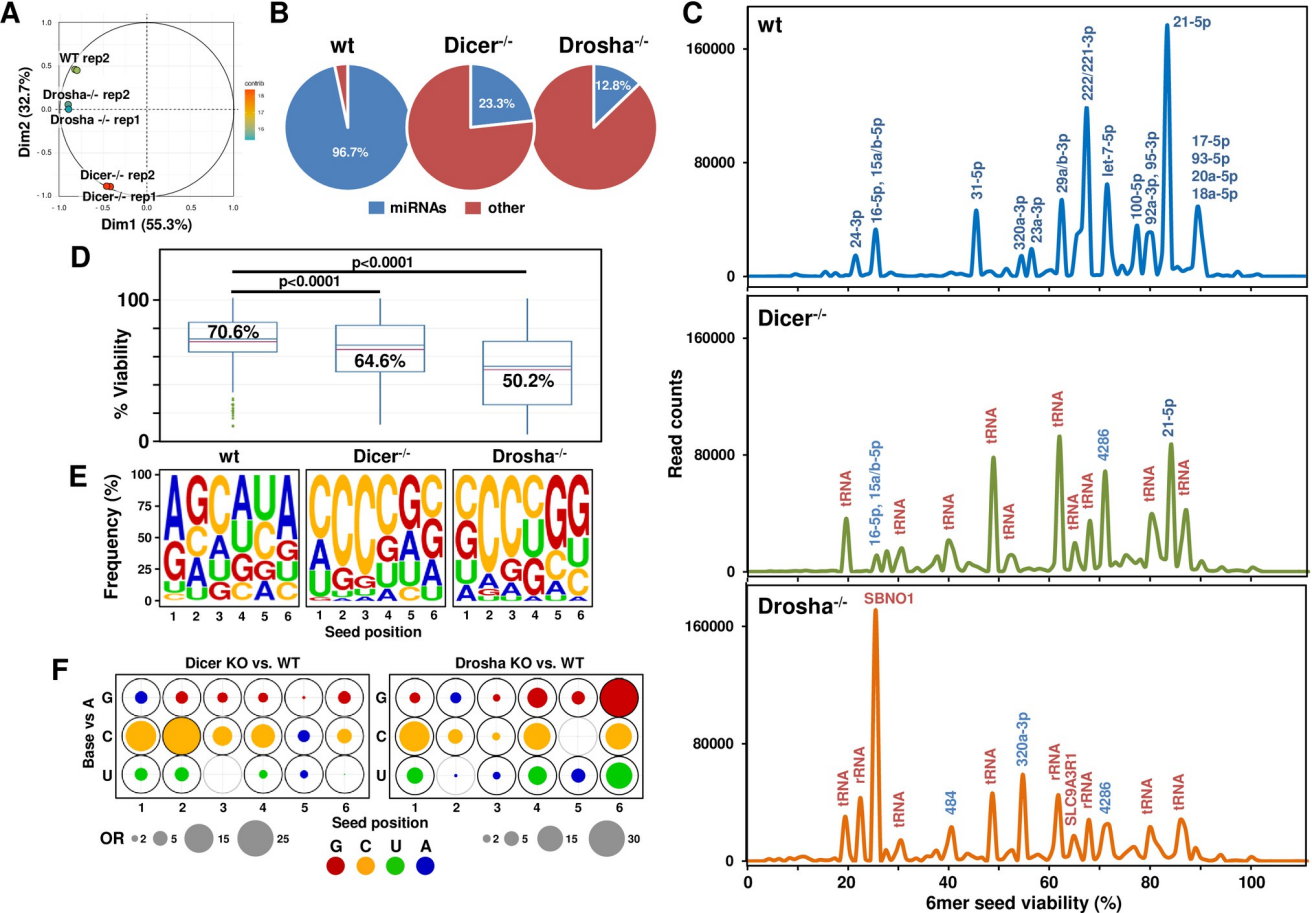
The RISC in HCT116 Dicer and Drosha k.o. cells is enriched in sRNAs with toxic 6mer seeds compared to wt cells. (A) Principal component analysis (PCA) plot illustrating the differences in RISC composition of Dicer and Drosha k.o. cells compared to HCT116 wt cells, and the reproducibility of technical and biological replicates. The x-axis represents dimension 1 (dim1) and explains 55.3% of the variance, while the y-axis represents dimension 2 (dim2) and explains 32.7% of the variance. Each cell type was analyzed as two biological replicates. Each spot represents a single replicate sample from each cell type. Green- HCT116 wt, blue- Drosha k.o. and red- Dicer k.o. (B) Pie charts showing RISC composition of HCT116 wt, Dicer and Drosha k.o. cells. Abundance of miRNAs is shown in blue and all other sRNAs in red. (C) RISC-bound sRNA Seed Tox graphs of HCT116 wt, Dicer k.o., and Drosha k.o. cells. When a peak is labeled with multiple miRNAs (blue), the most abundant one is listed first. RNAs are only labeled if they account for 1000 reads or more. miRNAs are labeled in blue, other sRNAs in red. (D) Average predicted 6mer seed toxicity of all RISC-bound sRNAs enriched in cells in C. p values were calculated using a Wilcoxon rank test. (E) Seed composition of all RISC-bound sRNAs enriched in cells in C. (F) Positional changes in the 6mer seed composition between genotypes. Filled circles at each position represent the odds ratio (OR) estimates comparing the odds of observing G, C and U vs. A between genotypes, based on the multinomial mixed effects model. A was set as the reference because A-rich 6mer seeds were the least toxic [16]. The outer circle corresponds to the largest observed OR for each pairwise genotype comparison (e.g., Dicer k.o. vs. wt), with bold black circles denoting statistical significance based on p-values adjusted using Tukey’s method. OR<1 estimates are represented with blue circles with area OR* = 1/OR, indicating that A is more likely in this position. Circle area is scaled to be proportional to the OR.

**Fig 3:**
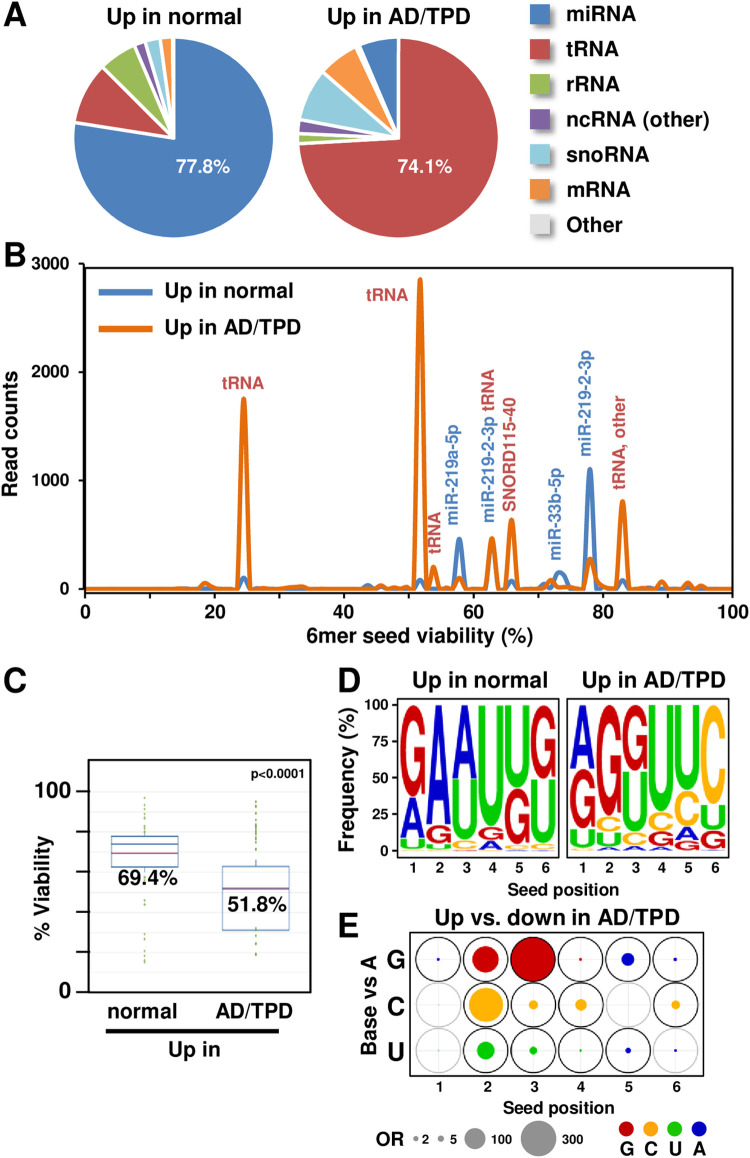
sRNAs enriched in AD brains contain more toxic seeds than in control brains. (A) Pie charts showing composition of sRNAs differentially expressed in normal and AD patient brains. (B) Seed Tox plots of sRNAs differentially expressed in brain samples in A. When a peak is labeled with multiple miRNAs (blue), the most abundant one is listed first. RNAs are only labeled if they account for 1000 reads or more. miRNAs are labeled in blue, other sRNAs in red. (C) Average predicted 6mer seed toxicity of all differentially regulated sRNAs in brain samples in A. p-value was calculated using a Wilcoxon rank test. (D) Seed composition of all RISC-bound differentially regulated sRNAs in brain samples in A. (E) Positional changes in the 6mer seed composition in reads significantly up- and down-regulated in AD/TPD patients compared to normal controls. Filled circles at each position represent the odds ratio (OR) estimates comparing the odds of observing G, C and U vs. A between groups (“Up” vs. “Down”), based on the multinomial mixed effects model. A was set as the reference because A-rich 6mer seeds were the least toxic [16]. The outer circle corresponds to the largest observed OR for each pairwise group comparison with bold black circles denoting statistical significance based on p-values adjusted using Tukey’s method. OR<1 estimates are represented with blue circles with area OR* = 1/OR, indicating that A is more likely in this position. Circle area is scaled to be proportional to the OR.

At this step, we have a table of raw read counts in each sample, which will become Output A. Reads originating from artificial sequences have been removed, as have rare reads. This table can be left as raw read counts, normalized to 1 million reads per column, or normalized and having differentially expressed reads identified with EdgeR. In each case, for ease of downstream processing, SPOROS extracts the 6mer seed from each read, and annotates each table row with the 6mer Seed, predicted 6mer seed toxicity, miRNA hit (if any) and RNA world hit (if any). The predicted 6mer seed toxicity is the % viability determined by transfecting three human and three mouse cell lines with siRNAs carrying all of the 4096 possible 6mer seeds ([16,17], 6merdb.org). By default, the average seed viabilities of the three human and three mouse cell lines are added. An option at this stage is to analyze all sRNAs/miRNAs in the data sets or only the ones significantly deregulated (<0.05 adjusted p-value, or just p-value) between conditions. We will show an example for each case (**Figs 2** and **3**). SPOROS automatically generates subfolders ("totalCounts" and "differential"). The differential analysis between two groups is performed by taking the significantly differentially expressed reads and calculating the delta read count (absolute normalized counts) for each row between two groups. Group1 is usually the control and Group2 the perturbed sample. Another layer of subfolders is generated allowing for the analysis of all sRNAs or only miRNAs ("sRNA" and "miRNA"). The hierarchy of subfolders generated is shown in the grey box in **Fig 1**. SPOROS then produces output files that can be used to create display figures (**Fig 1** and **Table 1**).

**Table 1:** Main output files of SPOROS.

Output	File name starts with	Description
A	A_normCounts	Contains all normalized data (each sample normalized to 1 million reads) with seeds, seed toxicities, miRNA, and RNA world information added. A file with raw counts ("A_rawCounts") is also generated for comparison.
B	B_collapsed	Generated by adding up and collapsing all rows that contain the same 6mer seed and either the same name in the miRNA or the RNA world blast columns. Counts are added up for each seed and miRNA combination. An intermediary file (file name starts with “Int_seedKeyed”) is also created, with each seed listed only once, even if it originates from multiple sRNAs. Depending on the species, subsequent steps will be done either with human or with the mouse seed viability data. Of note, in the analysis we use viability as a measure of predicted 6mer seed toxicity. Thus, seeds with a low viability percentage are considered more toxic and the ones with a high viability percentage are considered less toxic.
C	C_binned	Generated by aggregating all rows according to the predicted 6mer seed toxicity in 1% rounded steps (creating 1% sized bins). Counts are added up for all seeds that have toxicity within each 1% bin. These files contain a row for each bin even if the count is 0, allowing for easy plotting of the data. We use Excel to generate the graphs of read counts vs. viability bin, with line smoothing turned on. The resulting plot, the Seed Tox graph, allows one to display abundance of all miRNAs solely based on their predicted 6mer seed toxicity. The peaks in the Seed Tox graph can then be manually labeled with the most prominent miRNAs that fall within that particular viability range. This information can be obtained from Output file B. Most peaks will contain multiple miRNAs and we usually label the ones whose abundance is above a certain threshold (e.g., 1000 reads). We chose to generate this Seed Tox graph over a density plot (**S1 Fig**) because it allows to compare the actual read numbers in each peak between samples and different analyses.
D	D_toxAnalysis	These files are created from Output B to display the average seed viability in each analyzed group ("avg") or in individual samples ("rep1, rep2. . ."). The file expands each row according to the number of reads in that row after a normalization to 1000 rows (this normalization, i.e., dividing by 1000, prevents files from getting too large and thus increasing computational complexity of the downstream statistical analysis). The data column can be used in any standard statistics program to create a plot. We usually summarize the distributions using a boxplot and SPOROS creates a basic boxplot of all the defined average files. A nonparametric rank test can be performed to compare viability between groups. We currently use StatPlus (v.7.5) and the Wilcoxon ranksum test if there are two groups, or the Kruskal-Wallis test if there are more than 2 groups.
E	E_seedAnalysis	These files are also created from Output B in a similar way as Output D, except instead of expanding each row according to toxicity, the row is expanded according to the 6mer seed. SPOROS uses this output file to generate a custom Weblogo (http://weblogo.threeplusone.com/) to display nucleotide frequencies in each of the 6 seed positions. The Weblogo is generated as a high resolution png file.
F	F_seedExpand	These files are used in the statistical analysis comparing 6mer seed composition between different samples. It is generated from output E by further expanding the rows so that each nucleotide is in a different row for each seed, and rows are additionally indexed by position 1…6, thus representing each seed with 6 rows. As in Output D, seed counts are divided by 1000, and seeds with counts <1000 are omitted from this analysis. For example, if a particular seed occurs 5000 times in a sample, the rescaled seed count will be 5, and these seeds will be represented by 5×6 = 30 rows. In total, Output F contains 6N rows, where N is the total scaled seed count per sample.

In addition to these analyses, to analyze seed composition data, we developed a novel framework using multinomial mixed effects regression models [24]. A similar assumption that nucleotides follow a multinomial distribution at a given position has been used to test similarity between DNA sequences [25] and a multinomial logistic regression model without a random effect has been used for the analysis of codon frequencies [26].

Our multinomial mixed effects approach allows one to compare differences in sequence patterns between groups and positions and provides a statistical framework for both testing and estimation of such differences. Unlike analyses which compare counts between groups, here each sRNA seed represents the unit of analysis, and nucleotides are compared between positions and samples. Using terminology from the generalized linear mixed effects models literature [24,27] each observed read can be thought of as a "subject", represented by its seed, and the nucleotides in each position can be thought of as 6 potentially correlated measurements within a “subject”. Correlation between positions could occur, for example, if certain patterns are likely. An example would be the enrichment of Gs versus other nucleotides towards the 5’ end of the 6mer seed as reported [17]. The outcome variable in the model is the nucleotide in each position of each seed. We assume that at each position nucleotides follow a multinomial distribution with 4 possible outcomes (A, C, G, U), and, as in logistic regression, we must set one of the levels as the reference (we set A as the reference because A-rich 6mer seeds were the least toxic [16]). Group (e.g., genotype or experimental condition), position and their interaction are included as the "fixed effect" predictors, and the seed itself (i.e., the seed id) is included as the “random effect”. We note that a unique id is assigned to the seed within each read, rather than to each unique 6mer sequence, thus accounting for seed abundance by including a distinct “subject” for each observed sRNA read. For example, if two distinct sRNA’s with the same 6mer seed occur 50 and 100 times each, respectively, the model will include 150 “subjects” to represent this. Including the seed random effect in the model allows us to account for the potential within-seed correlation between positions. The estimated model provides tests of whether there are group or position differences, as well as the odds ratios (ORs) comparing the odds of observing G, C or U vs. A between groups at each position.

These models can be fitted using PROC GLIMMIX in SAS statistical software [28] but must be done outside of Code Ocean which does not currently support SAS.

Outputs A-E can be generated for either total reads (as in Fig 2) or only for those reads that are enriched in one set of samples relative to another (as in Fig 3). In the case of a differential analysis, SPOROS users create a comparisons table setting up one set of samples (denoted with 1) as Group1 and another set of samples (denoted with -1) as Group2. Samples irrelevant to a particular pairwise comparison are denoted in the comma separated table with a 0 (more detail is included with the SPOROS documentation on Github and in Code Ocean). The R package EdgeR [29] is used to identify reads differentially expressed within each pairwise comparison. Output A file names contain “diff” (adjusted p-value < 0.05 and logFC > 0.585 or < -0.585). These differentially regulated reads are used for Output C-E, see **Fig 3B–3D**. All final SPOROS output files that were used to generate Figs 2 and 3 are in S1 and S2 Datasets. They are also available within the Code Ocean capsule and can be recreated there from input data on demand.

## Results and discussion

### Example #1: analysis of RISC-bound sRNAs (all sRNAs)

For the first example we chose to analyze RISC-bound sRNAs isolated from wt HCT116 cells and cells deficient for either Drosha or Dicer (**Fig 2**). These two k.o. cells cannot produce canonical miRNAs or only at strongly reduced levels [21]. As previously described [16,22,30,31], we used a GW182 peptide coupled to GST to pull down all four Ago proteins which are critical for RISC formation and function [32]. A principal component analysis (PCA) shows all three genotypes cluster independently with biological replicates for each genotype tightly grouped together (**Fig 2A**). (PCA script in **S1 Text)**. Wild-type cells contained >96% RISC-bound miRNAs and this amount was reduced to ~23% in the Dicer k.o. cells and further reduced to ~13% in Drosha k.o. cells (**Fig 2B**).

When comparing the three genotypes using the Seed Tox graph, it became apparent that in both Dicer and Drosha k.o. cells, most miRNAs in the RISC were replaced by other sRNAs, most notably tRNA fragments (**Fig 2C**). This likely occurred because in contrast to normal cells [33,34], tumor cells maintain expression of Argonaute proteins in the absence of miRNAs [14].

The change in RISC composition in the two mutant cells resulted in a reduction in average seed viability of all reads (**Fig 2D**). This was most prominent for the Drosha k.o. cells which have the lowest amount of miRNAs, suggesting that endogenous miRNAs which carry mostly nontoxic seeds [30] likely protect cells from potentially toxic endogenous sRNAs entering the RISC. These sRNAs (e.g., tRNA or rRNA fragments) often contain C-rich sequences, which is likely the reason why the average 6mer seed composition in the RISC shifted towards C-richness in the mutant cells with sequences being somewhat more G-rich in the Drosha k.o. cell lines (**Fig 2E**). Together, these trends likely account for the more strongly reduced seed viability (**Fig 2D**) in these cells.

Multinomial mixed effects models revealed that differences between the three genotypes differ by position (p<0.0001, interaction term) (see https://github.com/ebartom/SPOROS and S3 Dataset). Model-based odds ratio (OR) estimates comparing G, C and U vs. A between genotypes are graphically summarized in **Fig 2F**, and the majority are statistically significant (dark black outer circles). For example, relative to A, C is more likely to occur in almost all positions of the seed in Dicer k.o. cells than in wt (OR = 5.9 to 28.4 in all positions represented by large orange circles, except position 5 where OR = 0.22 which is represented by a blue circle; p<0.0001 at all positions). Similarly, relative to A, G is significantly more likely to occur in Dicer k.o. than in wt in positions 2–6 (red circles), but the differences between genotypes are smaller (OR = 1.9 to 4.5; p<0.0001 at all positions).

We previously showed that these Drosha k.o. cells grow slower than their wt counterparts and knocking down Ago2 corrected the reduced growth rate [31], suggesting that endogenous sRNAs with toxic seeds that entered the RISC were causing a growth reduction. Importantly it demonstrated the relevance of including non-canonical sRNAs in the predicted 6mer seed toxicity analysis. Non-canonical sRNAs in the RISC also exert RNAi activity.

### Example #2: analysis of total cellular smRNAs (differentially expressed sRNAs)

The data set of the second example on sRNAs from AD and TPD brains [23]. The SPOROS analysis shows that sRNAs that are significantly enriched in either control or AD brains have a profound shift from mostly miRNAs to mainly tRNA fragments (**Fig 3A**), a phenomenon quite similar to that observed in the HCT116 Drosha k.o. cells in which miRNA biogenesis is impaired. To determine how this shift influences the predicted 6mer seed toxicity, we used SPOROS to do a differential analysis of the read counts made available in GEO (GSE63501), starting from a table of counts, and running a differential analysis. The Seed Tox graph based on Output B revealed a shift from mostly nontoxic miRNAs in control brains to sRNAs with more toxic seeds in AD patients. Consistent with the published data, the most profoundly downregulated miRNA in the AD/TPD brains is miR-219 [23]. However, most changes are seen in tRNA fragments many of which carry toxic seeds (**Fig 3B**). This shift to seeds with lower viability also became apparent in the average predicted 6mer seed toxicity analysis of all reads (**Fig 3C**) and this was mostly due to a significant increase in Gs towards the 5’ end of the 6mer seed of the sRNA in the AD brains (**Fig 3D**). This was confirmed by model-based OR estimates (see https://github.com/ebartom/SPOROS and S4 Dataset) comparing G, C and U vs. A between groups of seeds that are significantly enriched in the AD/TPD brains (“Up” group) or enriched in normal brains (“Down” group) which are graphically summarized in **Fig 3E**. The majority of comparisons are statistically significant (dark black outer circles). Relative to A, G is strikingly more likely to occur in positions 2 and 3 of seeds in the “Up” vs. “Down” groups (OR = 175.7 and 490.7; p<0.0001). Similarly, relative to A, C is significantly more likely to occur in “Up” vs. “Down” group in positions 2–4 (OR = 20.2 to 294.5; p<0.0001). This analysis suggests that in AD brains the repertoire of sRNAs available to potentially function through RNAi are predicted to be more toxic.

The human genome contains a large number of predicted miRNAs [18]. They have been shown to regulate almost all biological processes and to be deregulated in countless disease states [35]. The state-of-the-art method to analyze miRNAs is by RNA Seq. Almost all RNA Seq data are analyzed with the goal of identifying differentially expressed genes after aligning reads to a genome and employing appropriate normalization to account for transcript length (reads / fragments per kilobase gene model) or variation between data sets. These types of analyses, while standard, have major shortcomings and all methods to normalize these large data sets to identify and study individual miRNAs have shortcomings [36]. The analysis of predicted 6mer seed toxicity does not focus on individual miRNAs or canonical miRNA families but treats all sRNAs solely based on their position 2–7. This allows for the ranking of all sRNAs into blocks from highly toxic to nontoxic sRNAs and often it is the balance between the sum of toxic versus nontoxic sRNAs bound to the RISC that determines the responses of cells [30]. The analyses in the two examples presented (cancer and AD), were chosen to demonstrate the power and the potential of the SPOROS pipeline to predict 6mer seed toxicity. In example #1, the data support the view that most miRNAs carry nontoxic seeds and are in part protecting cells from loading of endogenous sRNAs, which by nature are more G/C rich and hence when entering the RISC, exert toxicity. In the AD example #2, the data suggest that in AD brains, equilibrium shifts away from nontoxic miRNAs to more toxic sRNAs, such as tRNA fragments. While this result needs to be validated by performing Ago pull-down experiments with AD patient brains, it is intriguing that a recent study in another neurodegenerative disease, Huntington’s disease (HD), reported that sRNAs isolated from HD brains were toxic when injected into mouse brains [37]. This was shown in part to be due to an increase in tRNAs in the disease brains when compared to normal control brains. An analysis of predicted 6mer seed toxicity could become important not only in the context of cancer but also in other diseases (recently reviewed in [15]). The methods we have developed to predict 6mer seed toxicity will allow for further study of the role of DISE in multiple disease situations.

## Supporting information

S1 Fig Displaying predicted 6mer seed toxicity in a density plot. RISC-bound sRNA predicted 6mer seed toxicity data of HCT116 wt, Dicer k.o., and Drosha k.o. cells displayed as density plots. The same source data were used to generate **Fig 2C**.
(TIF)

S1 Table Data on human mature miRNAs used in the BLAST search. Data are from miRBase supplemented with adapter and artificial sequences to ensure that these are appropriately flagged. This file also contains four HIV-1 encoded miRNAs.
(PDF)

S2 Table Data on mouse mature miRNAs used in the BLAST search. Data are from miRBase supplemented with adapter and artificial sequences to ensure that these are appropriately flagged.
(PDF)

S1 Text Script used to generate Fig 2A. (PDF)

S1 Dataset Final SPOROS output files Fig 2. (ZIP)

S2 Dataset Final SPOROS output files Fig 3. (ZIP)

S3 Dataset Code for Fig 2. (ZIP)

S4 Dataset Code for Fig 3. (ZIP)
