# Supplementary figures and images for: SPOROS: A pipeline to analyze DISE/6mer seed toxicity
Source: PLoS Comput Biol. 2022 Mar 31;18(3):e1010022. doi: 10.1371/journal.pcbi.1010022 (PMC9004739; doi:10.1371/journal.pcbi.1010022)

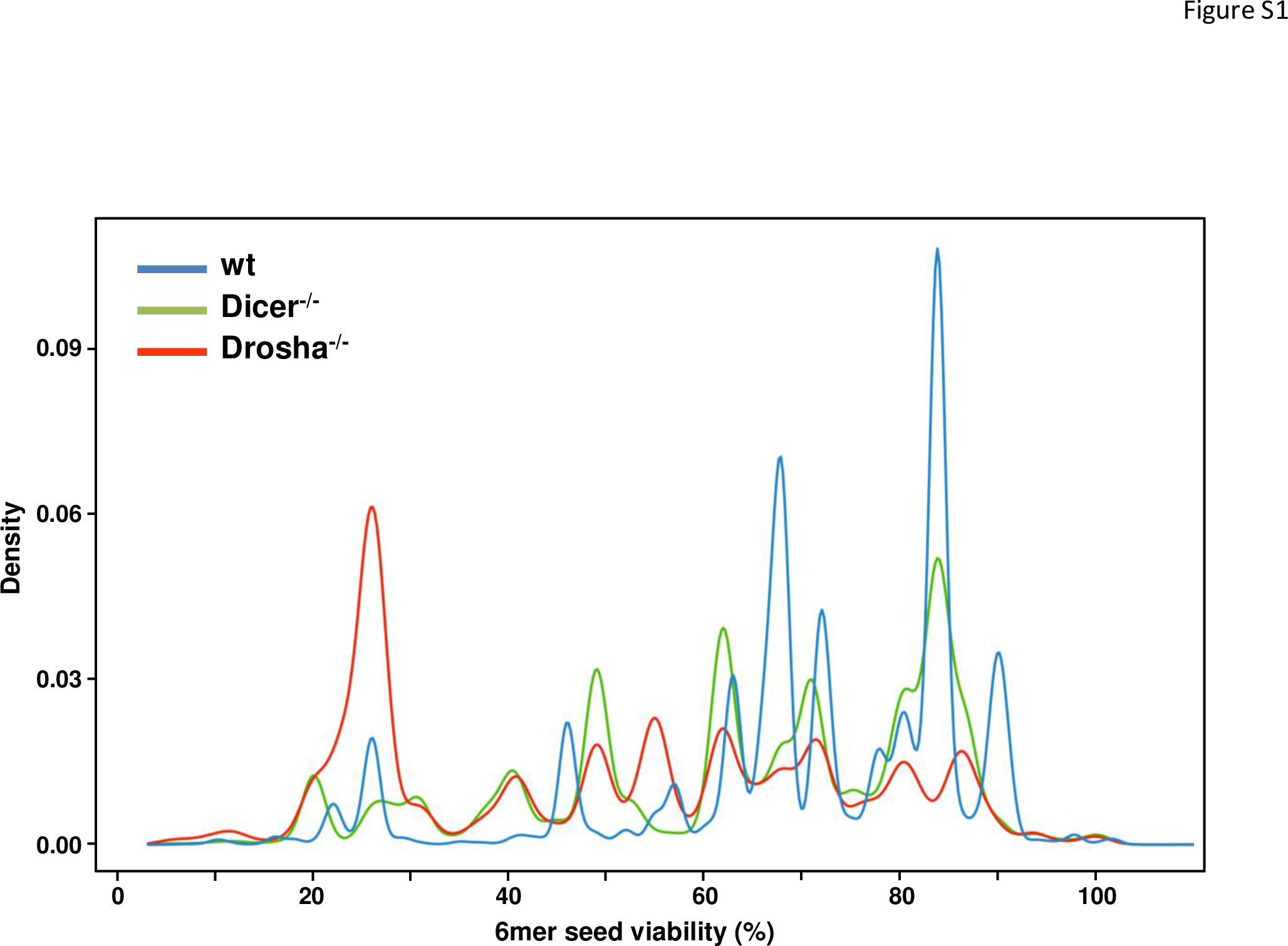

Supplement: S1 Fig — RISC-bound sRNA predicted 6mer seed toxicity data of HCT116 wt, Dicer k.o., and Drosha k.o. cells displayed as density plots. The same source data were used to generate Fig 2C. (TIF) [file pcbi.1010022.s001.tif]

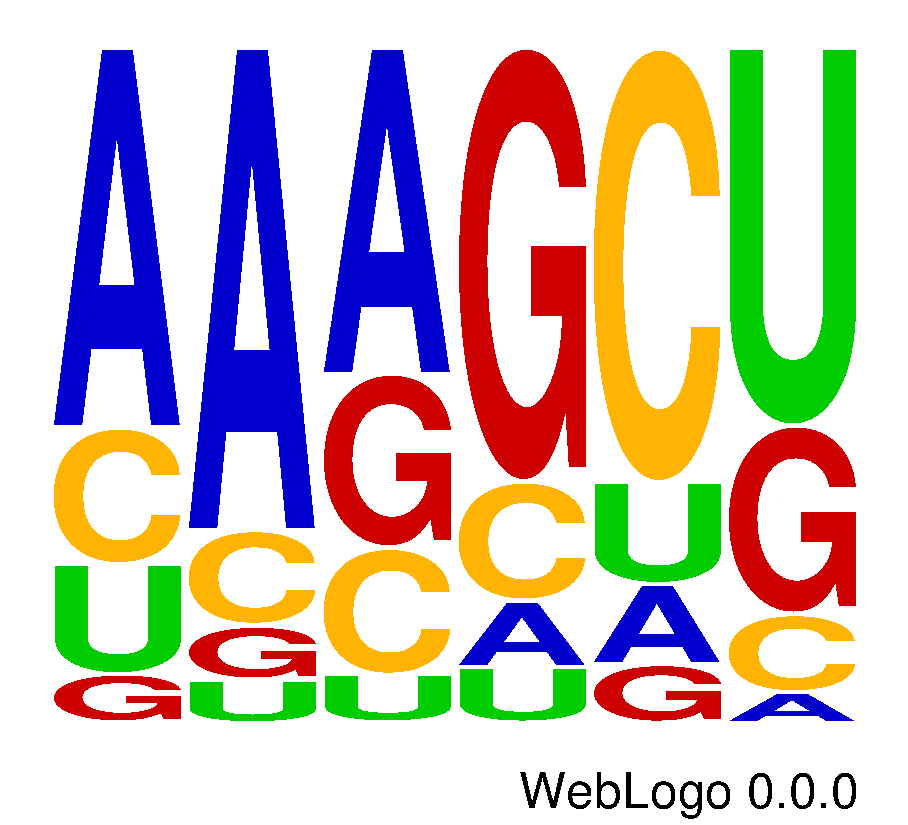

Supplement: S1 Dataset — (ZIP) [file pcbi.1010022.s005.zip › Final SPOROS output Figure 2/miRNA/E_seedAnalysis.DroshaKO.rep1.miRNA.Figure2.png]

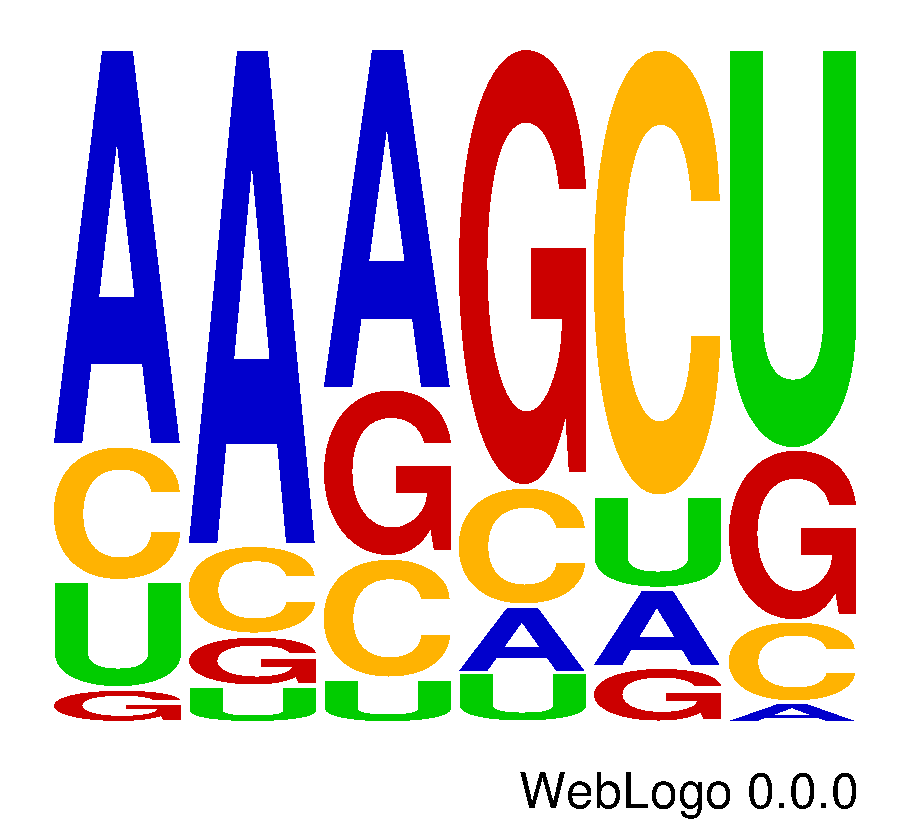

Supplement: S1 Dataset — (ZIP) [file pcbi.1010022.s005.zip › Final SPOROS output Figure 2/miRNA/E_seedAnalysis.DroshaKO.rep2.miRNA.Figure2.png]

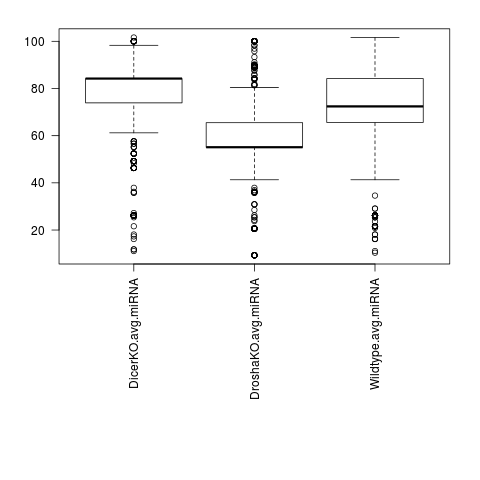

Supplement: S1 Dataset — (ZIP) [file pcbi.1010022.s005.zip › Final SPOROS output Figure 2/miRNA/D_toxAnalysis.combined.miRNA.txt.box.png]

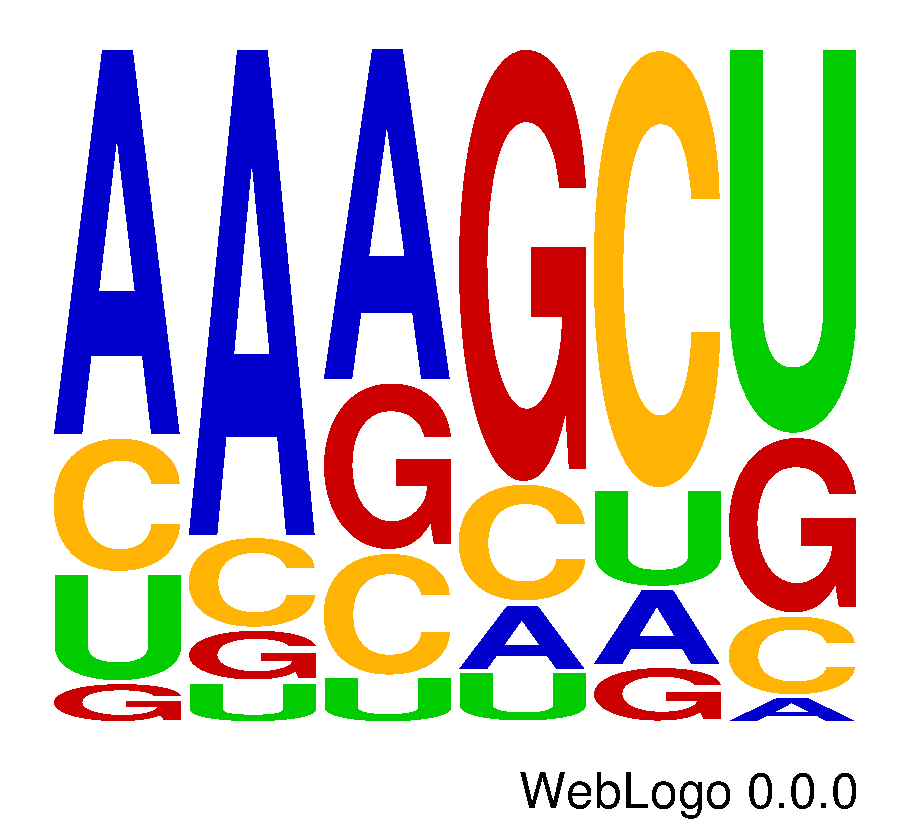

Supplement: S1 Dataset — (ZIP) [file pcbi.1010022.s005.zip › Final SPOROS output Figure 2/miRNA/E_seedAnalysis.DroshaKO.avg.miRNA.Figure2.png]

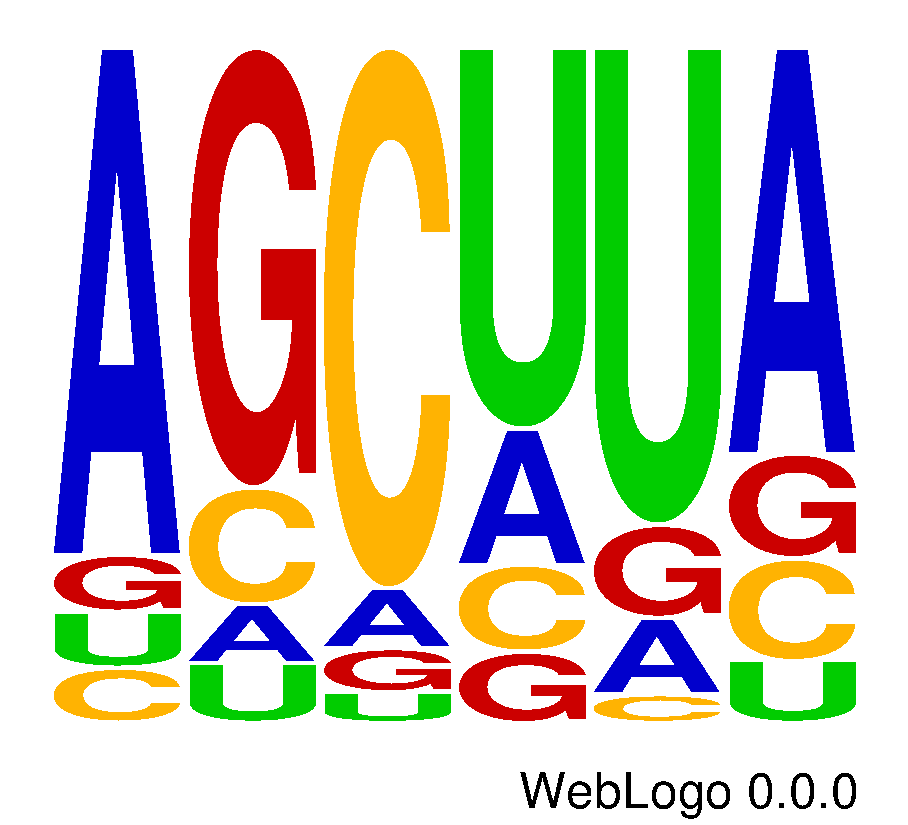

Supplement: S1 Dataset — (ZIP) [file pcbi.1010022.s005.zip › Final SPOROS output Figure 2/miRNA/E_seedAnalysis.DicerKO.rep1.miRNA.Figure2.png]

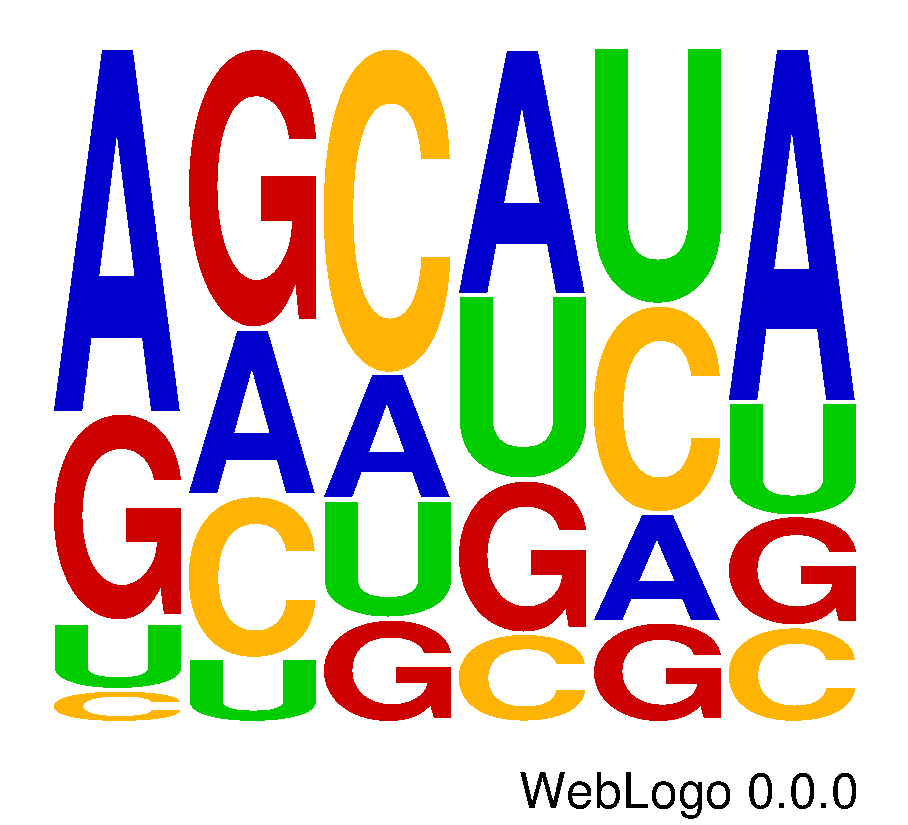

Supplement: S1 Dataset — (ZIP) [file pcbi.1010022.s005.zip › Final SPOROS output Figure 2/miRNA/E_seedAnalysis.Wildtype.rep2.miRNA.Figure2.png]

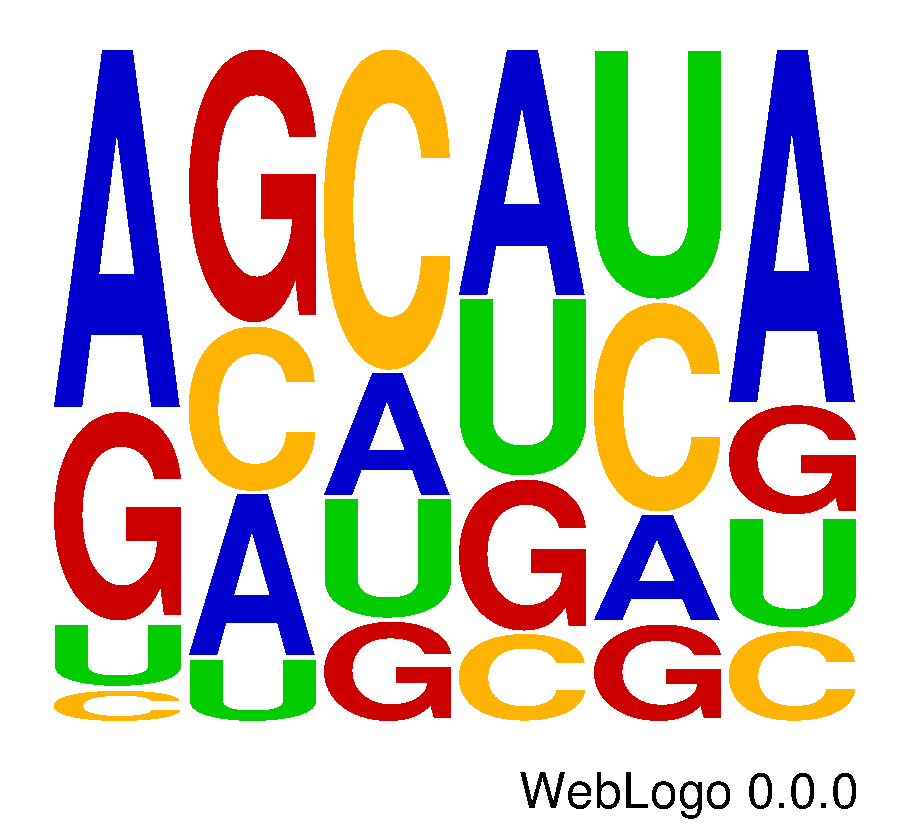

Supplement: S1 Dataset — (ZIP) [file pcbi.1010022.s005.zip › Final SPOROS output Figure 2/miRNA/E_seedAnalysis.Wildtype.avg.miRNA.Figure2.png]

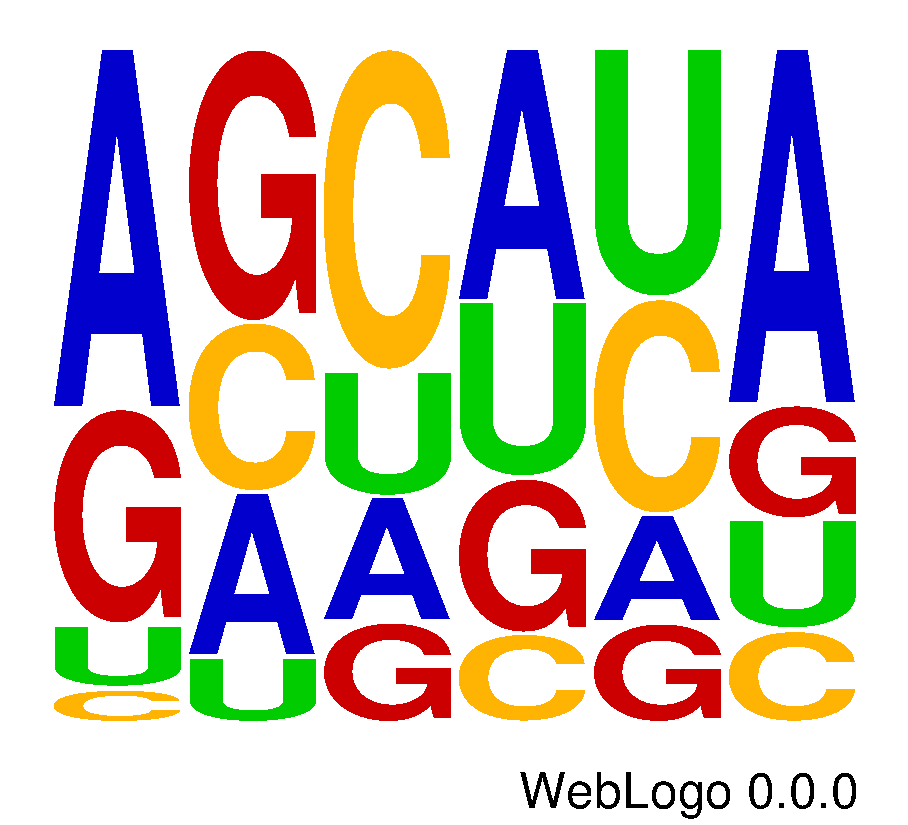

Supplement: S1 Dataset — (ZIP) [file pcbi.1010022.s005.zip › Final SPOROS output Figure 2/miRNA/E_seedAnalysis.Wildtype.rep1.miRNA.Figure2.png]

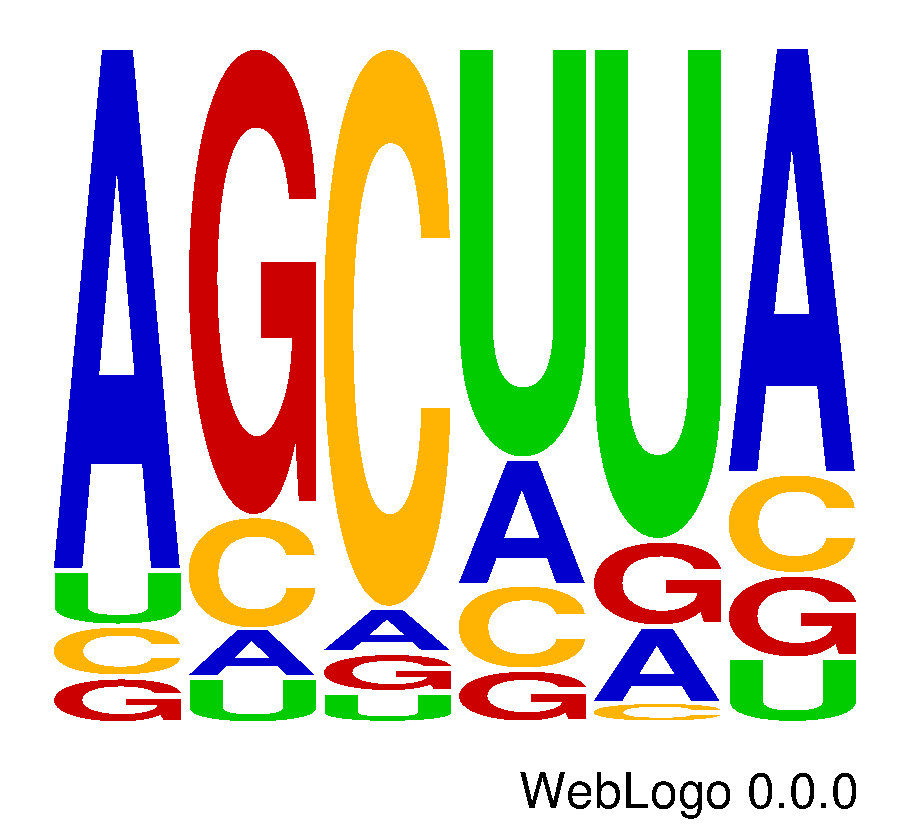

Supplement: S1 Dataset — (ZIP) [file pcbi.1010022.s005.zip › Final SPOROS output Figure 2/miRNA/E_seedAnalysis.DicerKO.rep2.miRNA.Figure2.png]

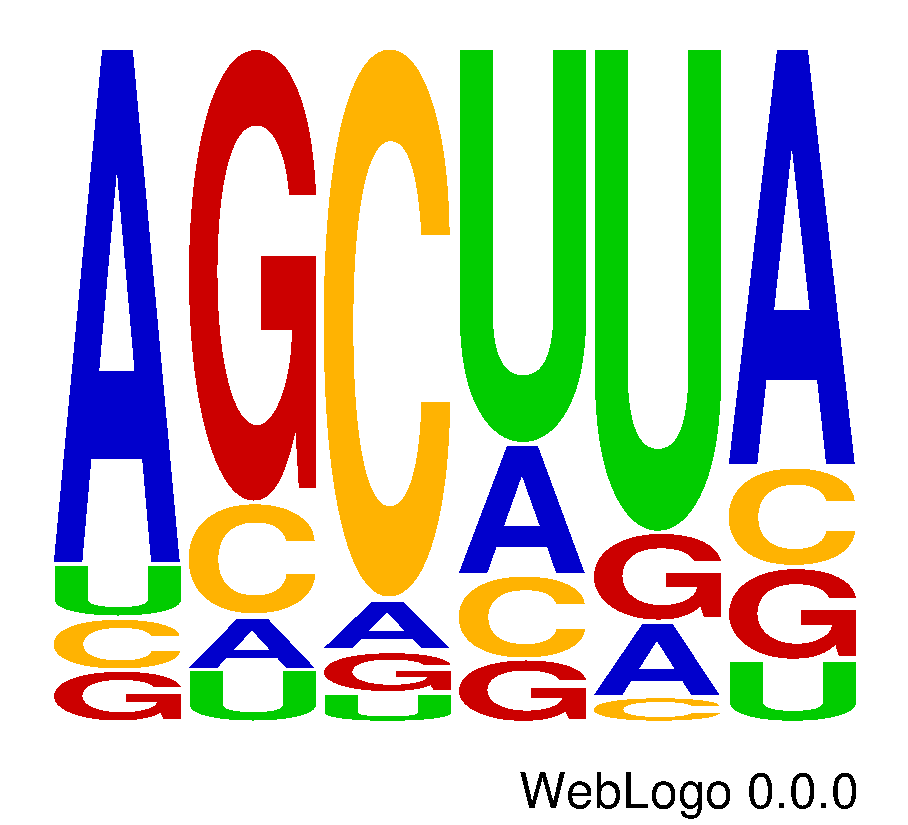

Supplement: S1 Dataset — (ZIP) [file pcbi.1010022.s005.zip › Final SPOROS output Figure 2/miRNA/E_seedAnalysis.DicerKO.avg.miRNA.Figure2.png]

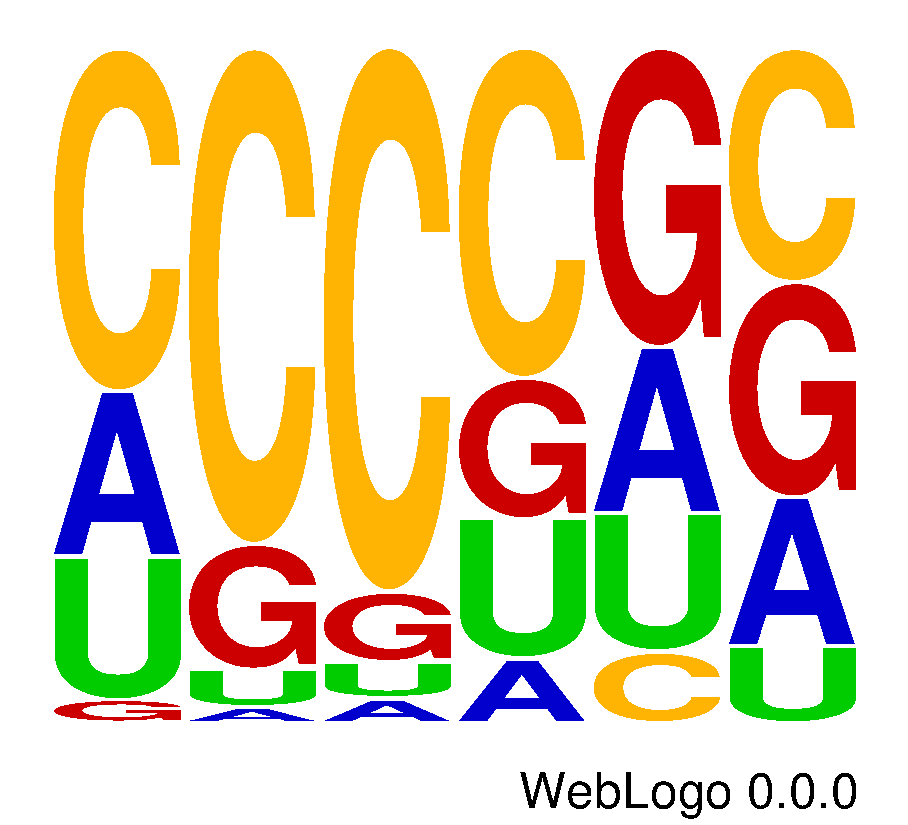

Supplement: S1 Dataset — (ZIP) [file pcbi.1010022.s005.zip › Final SPOROS output Figure 2/sRNA/E_seedAnalysis.DicerKO.avg.sRNA.Figure2.png]

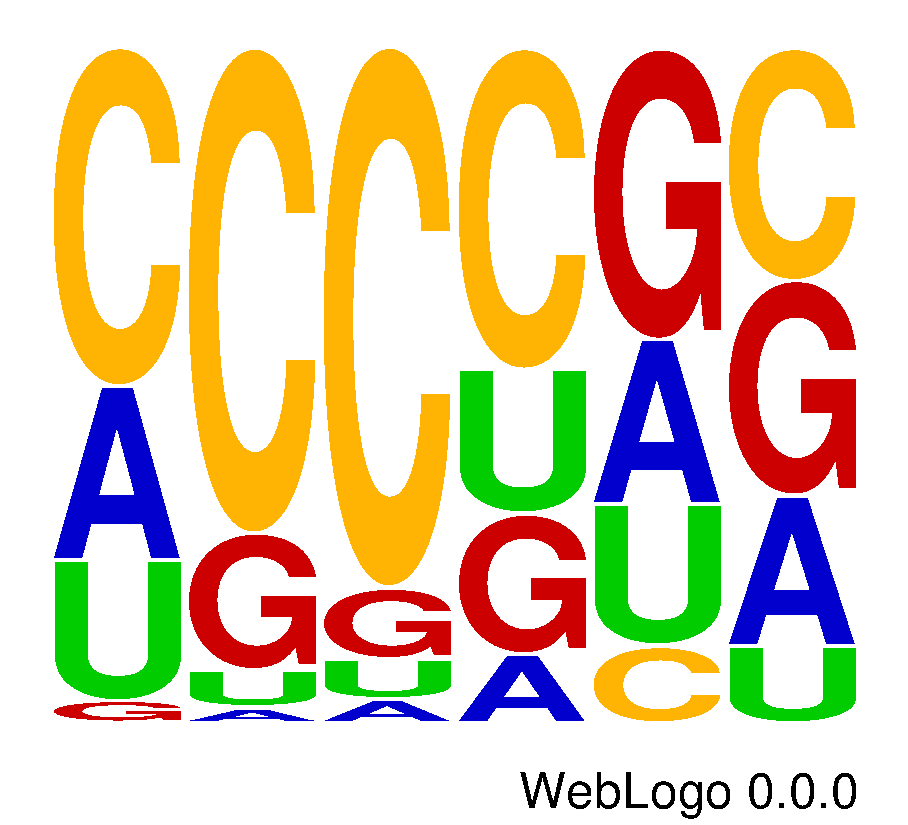

Supplement: S1 Dataset — (ZIP) [file pcbi.1010022.s005.zip › Final SPOROS output Figure 2/sRNA/E_seedAnalysis.DicerKO.rep2.sRNA.Figure2.png]

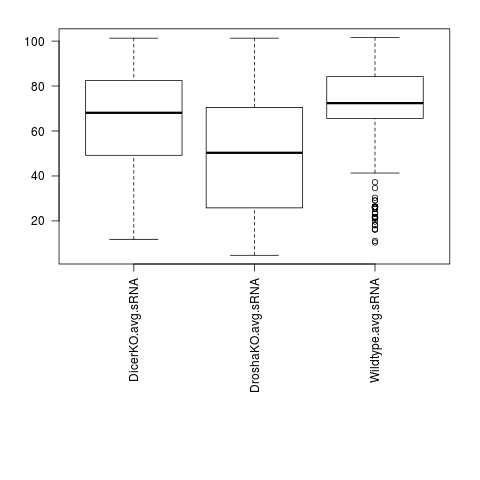

Supplement: S1 Dataset — (ZIP) [file pcbi.1010022.s005.zip › Final SPOROS output Figure 2/sRNA/D_toxAnalysis.combined.sRNA.txt.box.png]

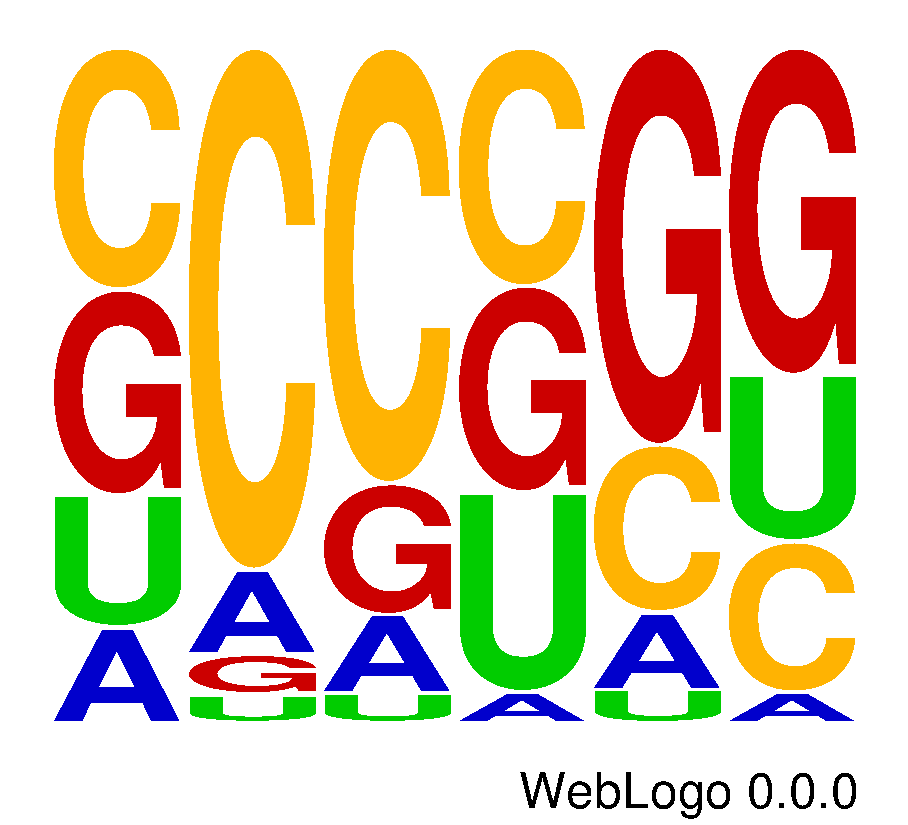

Supplement: S1 Dataset — (ZIP) [file pcbi.1010022.s005.zip › Final SPOROS output Figure 2/sRNA/E_seedAnalysis.DroshaKO.rep2.sRNA.Figure2.png]

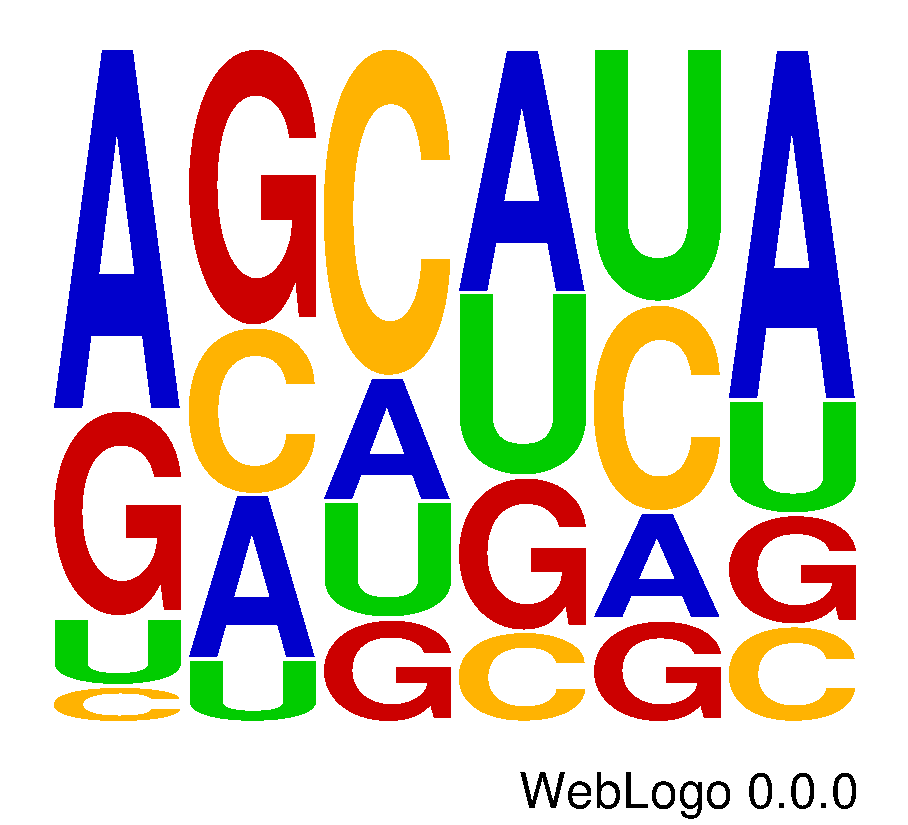

Supplement: S1 Dataset — (ZIP) [file pcbi.1010022.s005.zip › Final SPOROS output Figure 2/sRNA/E_seedAnalysis.Wildtype.rep2.sRNA.Figure2.png]

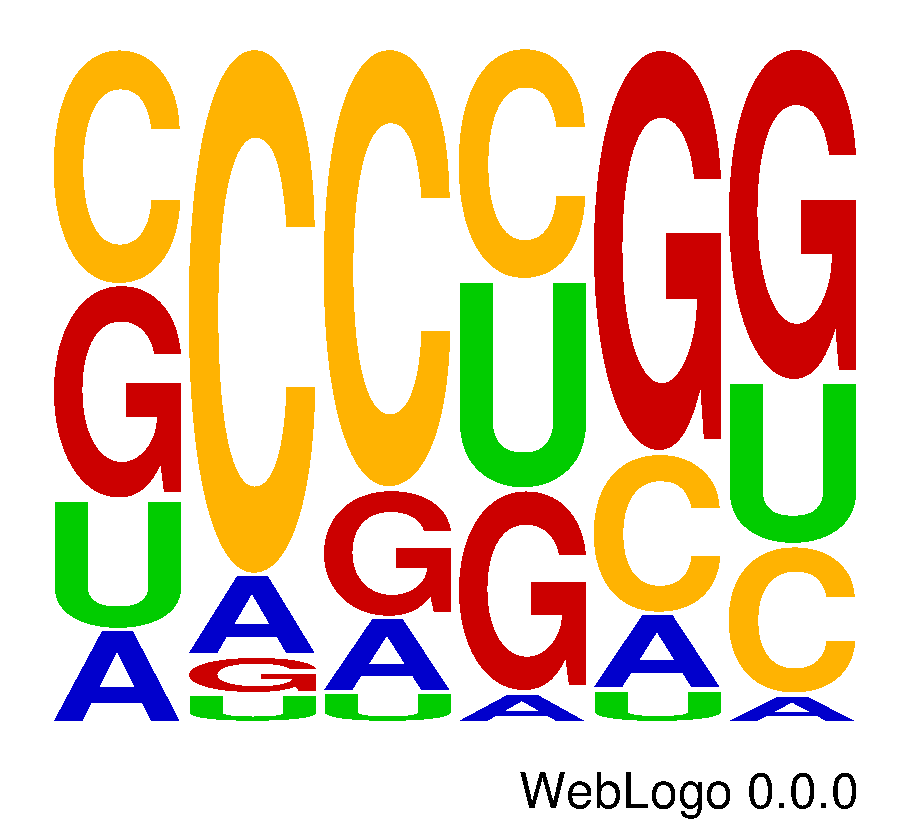

Supplement: S1 Dataset — (ZIP) [file pcbi.1010022.s005.zip › Final SPOROS output Figure 2/sRNA/E_seedAnalysis.DroshaKO.avg.sRNA.Figure2.png]

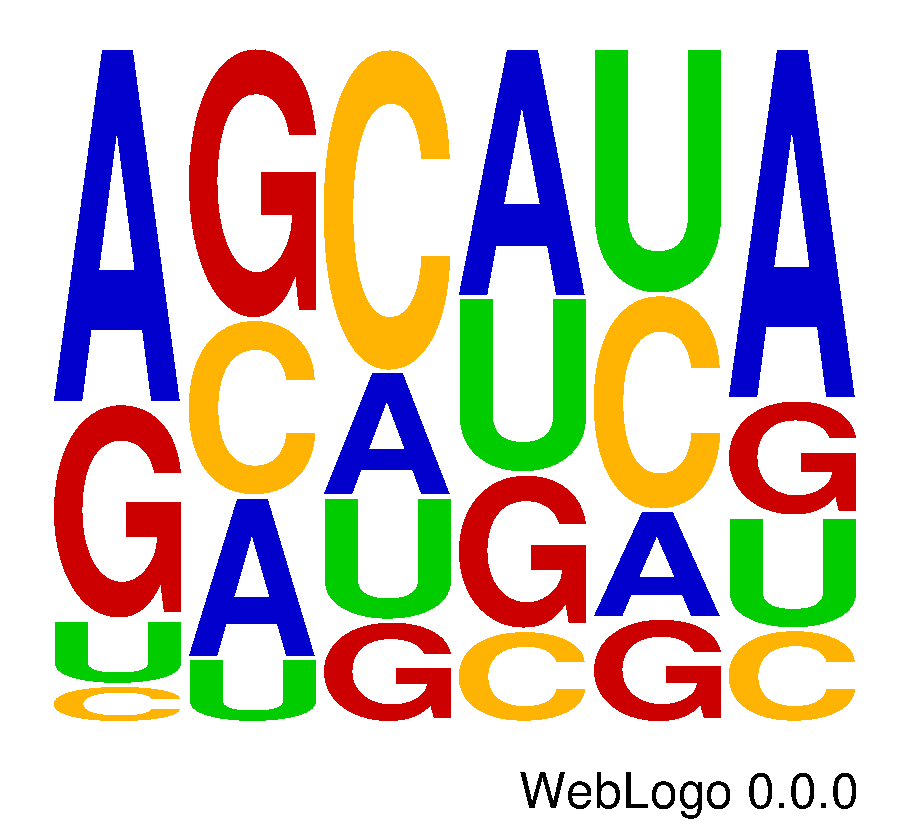

Supplement: S1 Dataset — (ZIP) [file pcbi.1010022.s005.zip › Final SPOROS output Figure 2/sRNA/E_seedAnalysis.Wildtype.rep1.sRNA.Figure2.png]

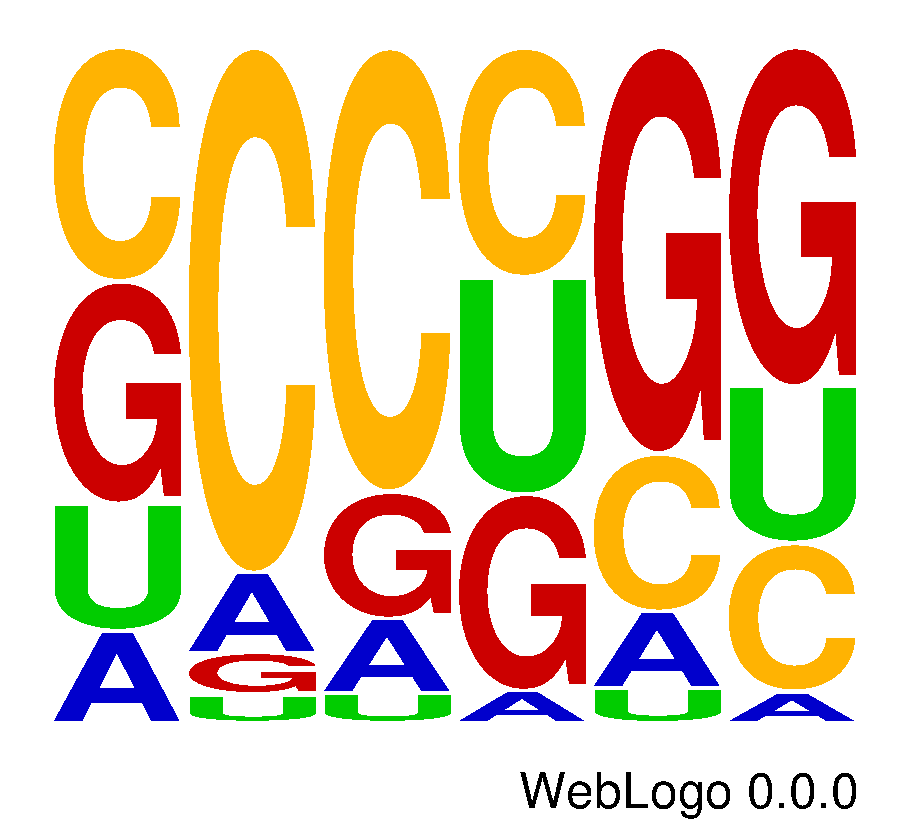

Supplement: S1 Dataset — (ZIP) [file pcbi.1010022.s005.zip › Final SPOROS output Figure 2/sRNA/E_seedAnalysis.DroshaKO.rep1.sRNA.Figure2.png]

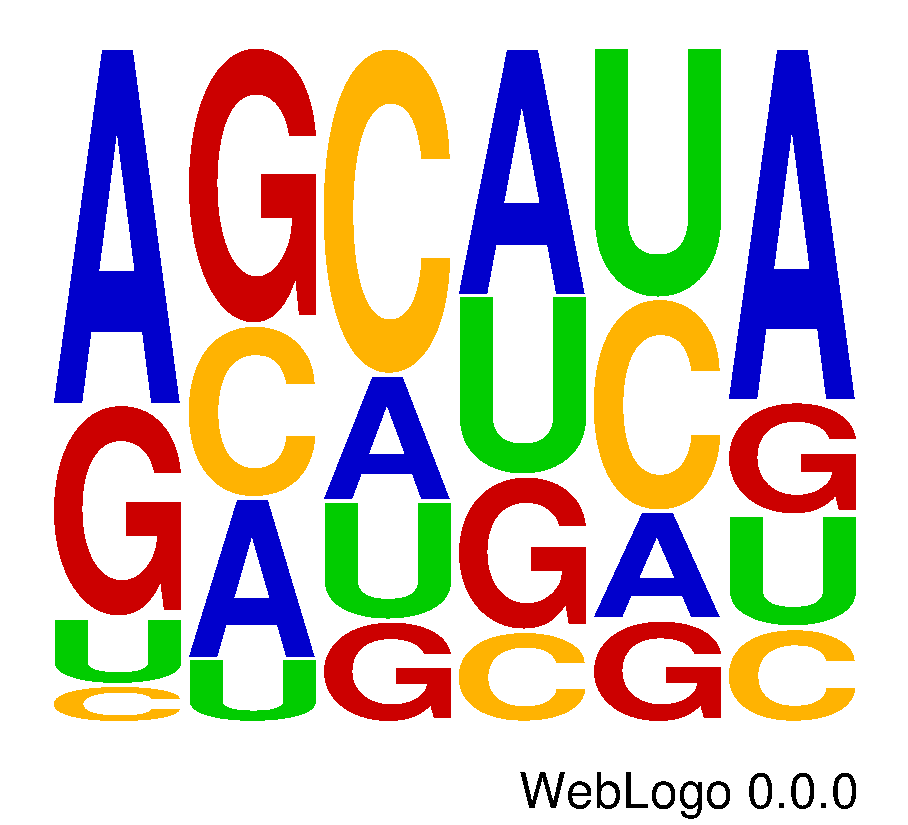

Supplement: S1 Dataset — (ZIP) [file pcbi.1010022.s005.zip › Final SPOROS output Figure 2/sRNA/E_seedAnalysis.Wildtype.avg.sRNA.Figure2.png]

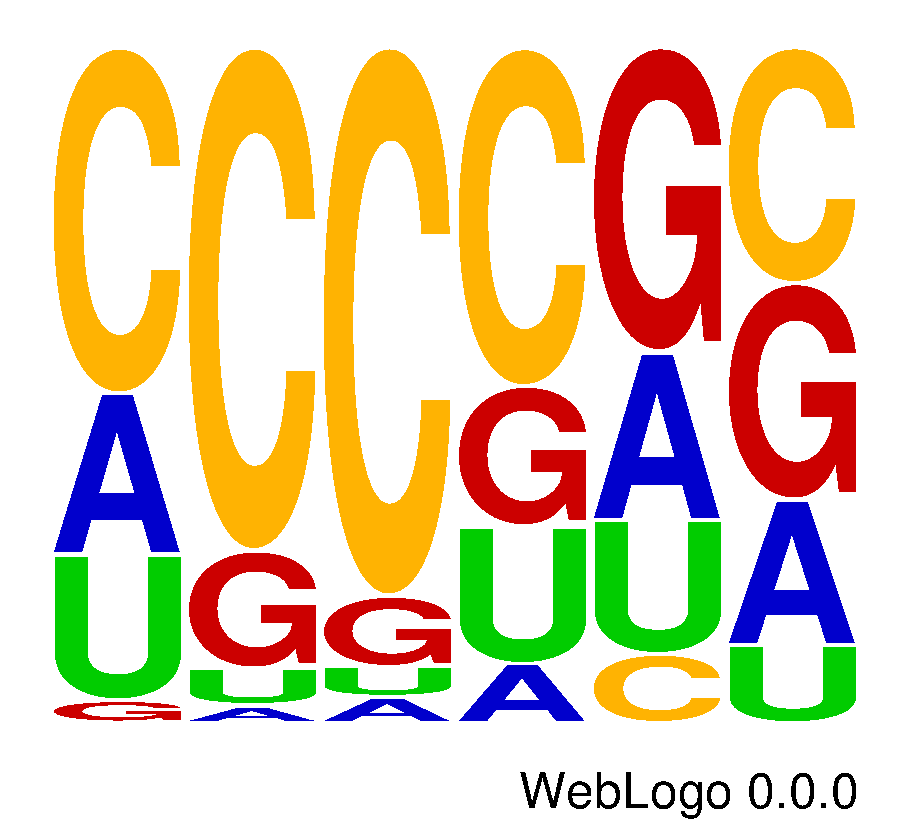

Supplement: S1 Dataset — (ZIP) [file pcbi.1010022.s005.zip › Final SPOROS output Figure 2/sRNA/E_seedAnalysis.DicerKO.rep1.sRNA.Figure2.png]

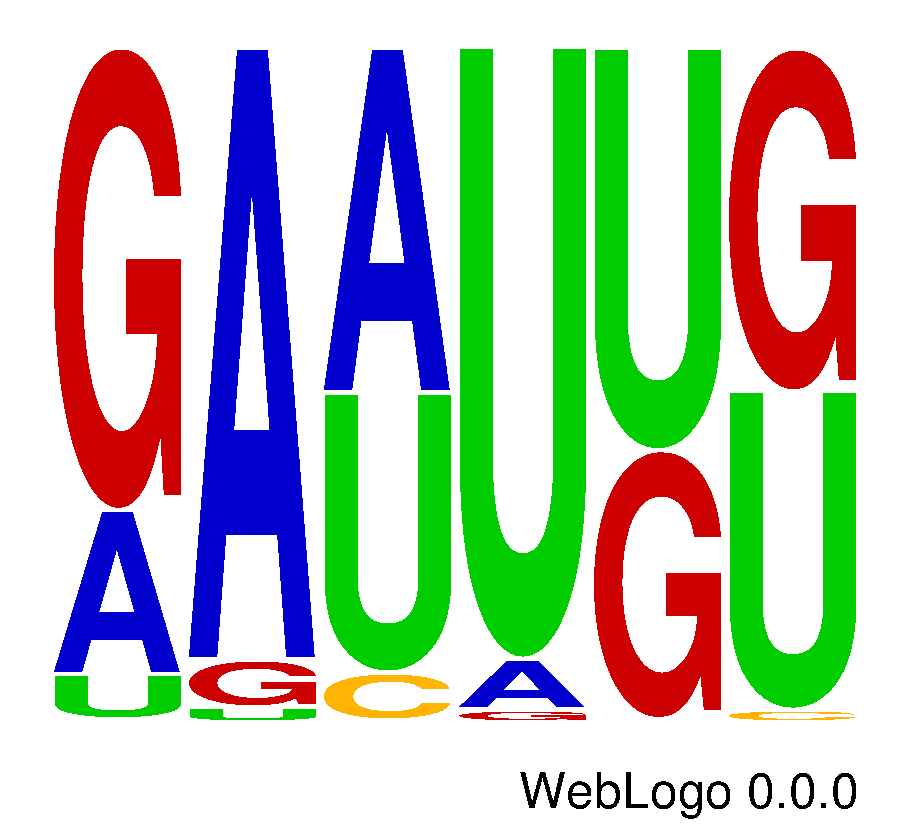

Supplement: S2 Dataset — (ZIP) [file pcbi.1010022.s006.zip › Final SPOROS output Figure 3/differential/ADnTPDvsCtrl/adjp/miRNA/E_seedAnalysis.Delta.miRNA.dn.png]

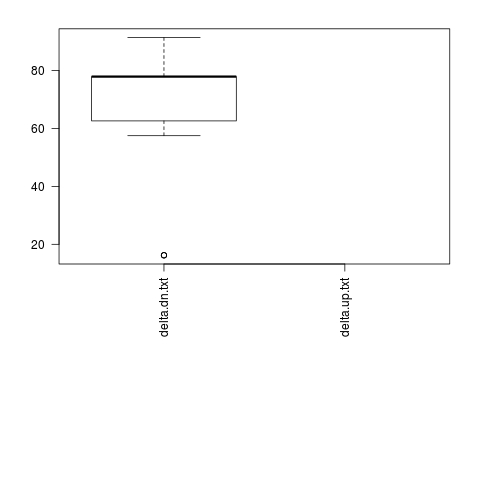

Supplement: S2 Dataset — (ZIP) [file pcbi.1010022.s006.zip › Final SPOROS output Figure 3/differential/ADnTPDvsCtrl/adjp/miRNA/D_toxAnalysis.combined.miRNA.txt.box.png]

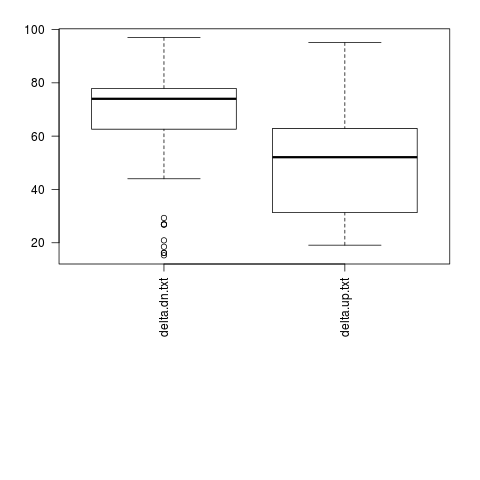

Supplement: S2 Dataset — (ZIP) [file pcbi.1010022.s006.zip › Final SPOROS output Figure 3/differential/ADnTPDvsCtrl/adjp/sRNA/D_toxAnalysis.combined.sRNA.txt.box.png]

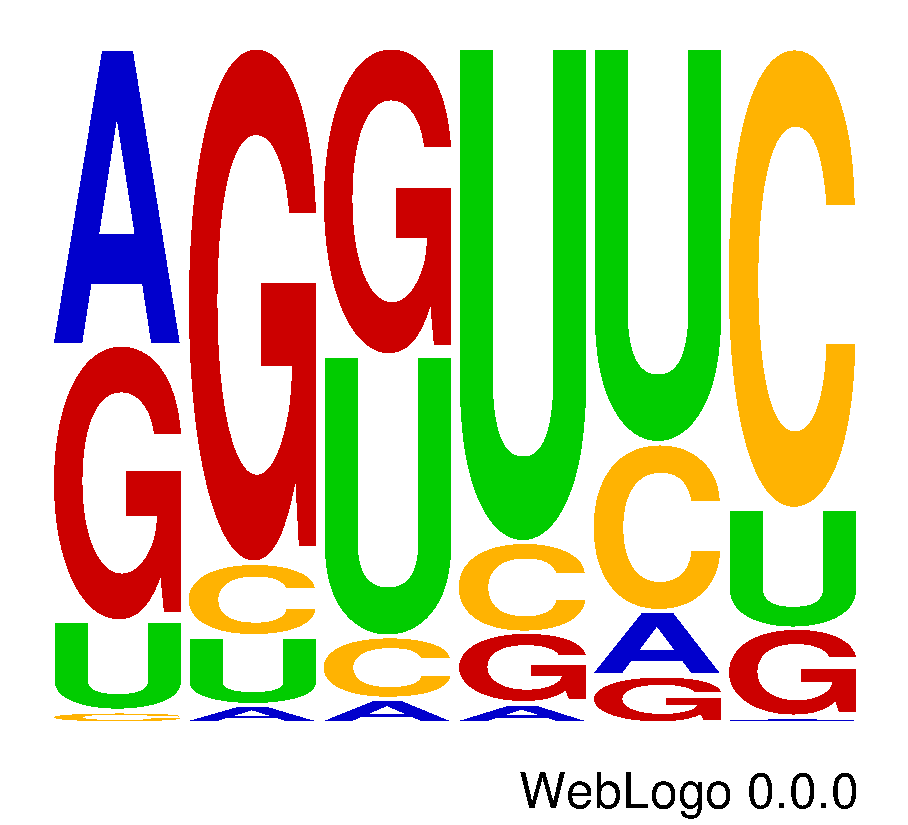

Supplement: S2 Dataset — (ZIP) [file pcbi.1010022.s006.zip › Final SPOROS output Figure 3/differential/ADnTPDvsCtrl/adjp/sRNA/E_seedAnalysis.Delta.sRNA.up.png]

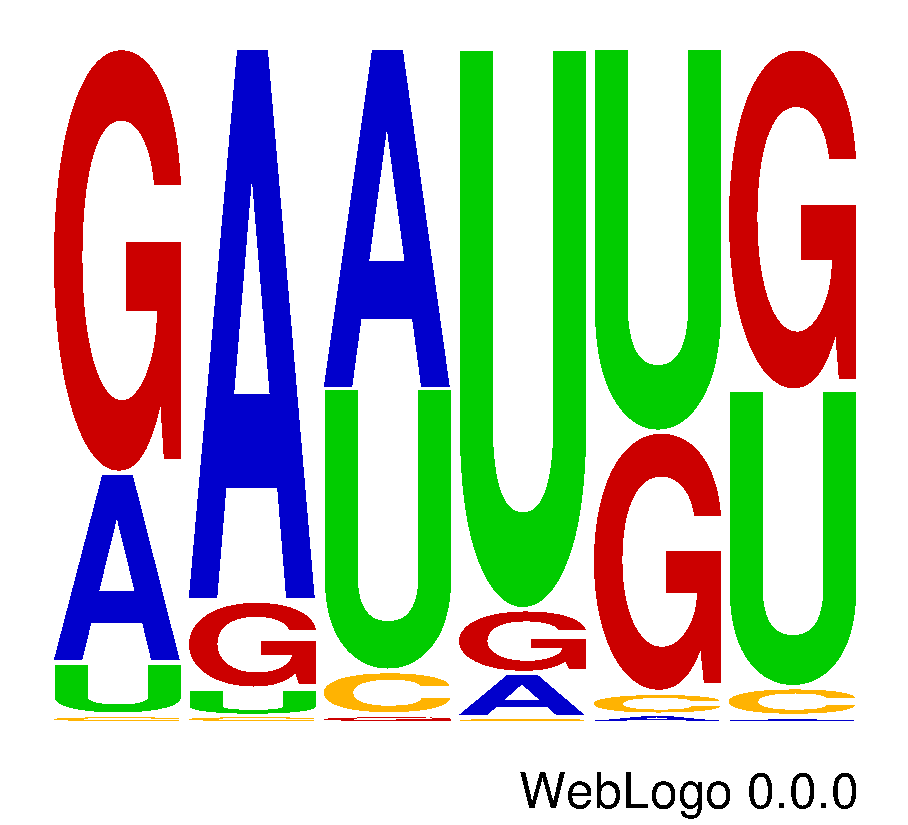

Supplement: S2 Dataset — (ZIP) [file pcbi.1010022.s006.zip › Final SPOROS output Figure 3/differential/ADnTPDvsCtrl/adjp/sRNA/E_seedAnalysis.Delta.sRNA.dn.png]

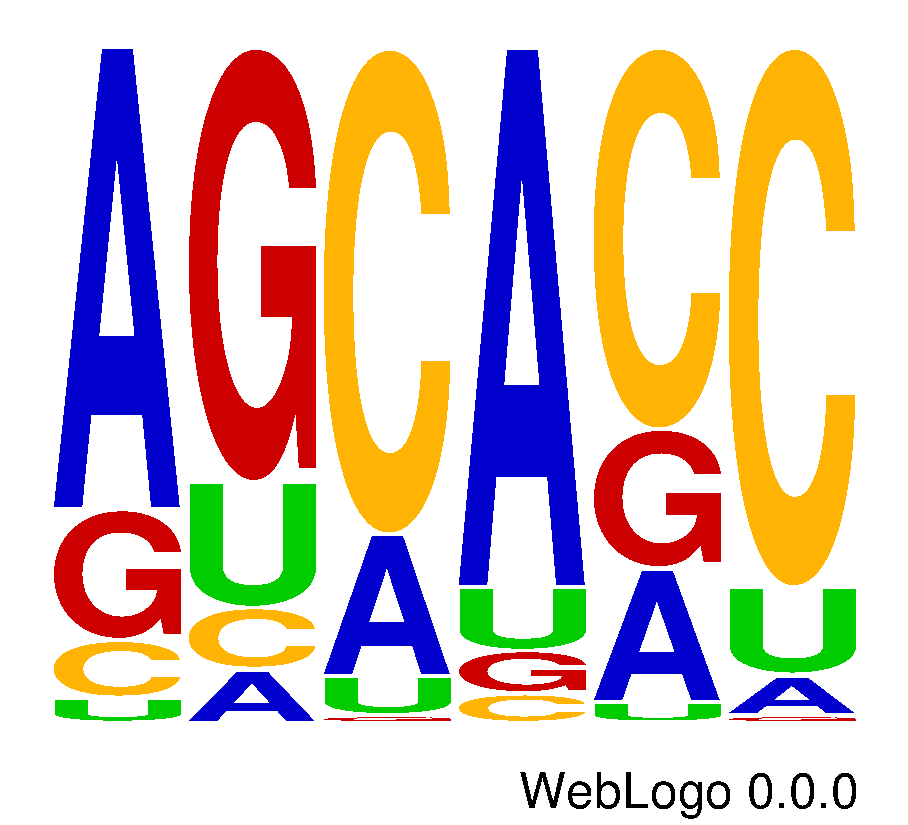

Supplement: S2 Dataset — (ZIP) [file pcbi.1010022.s006.zip › Final SPOROS output Figure 3/differential/ADnTPDvsCtrl/pvalue/miRNA/E_seedAnalysis.Delta.miRNA.up.png]

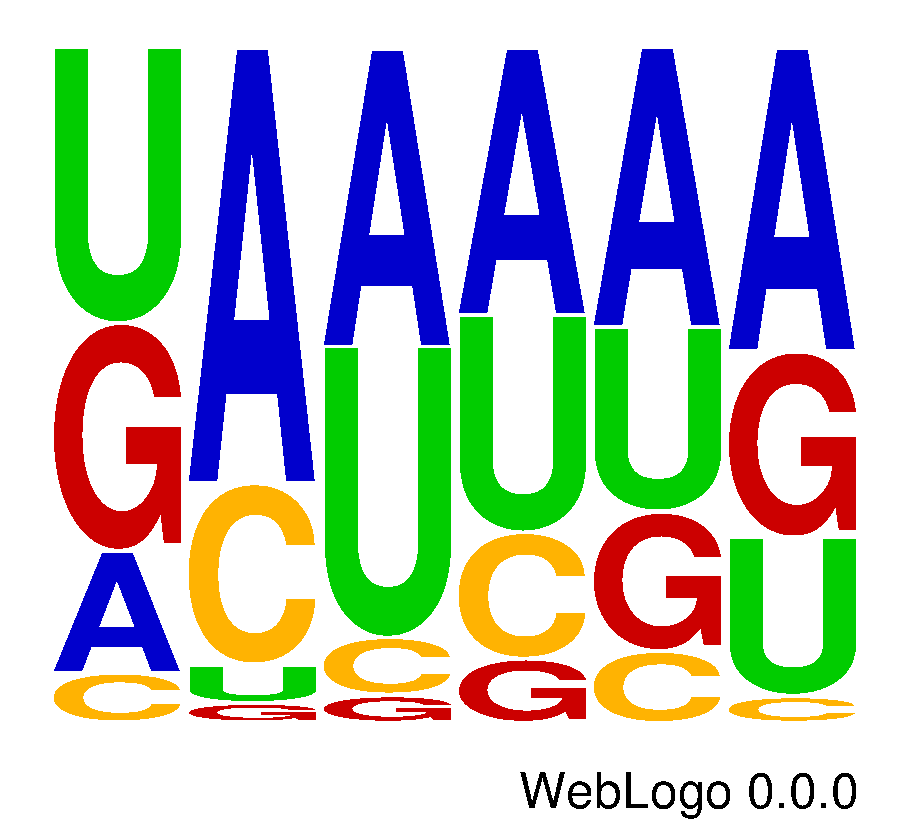

Supplement: S2 Dataset — (ZIP) [file pcbi.1010022.s006.zip › Final SPOROS output Figure 3/differential/ADnTPDvsCtrl/pvalue/miRNA/E_seedAnalysis.Delta.miRNA.dn.png]

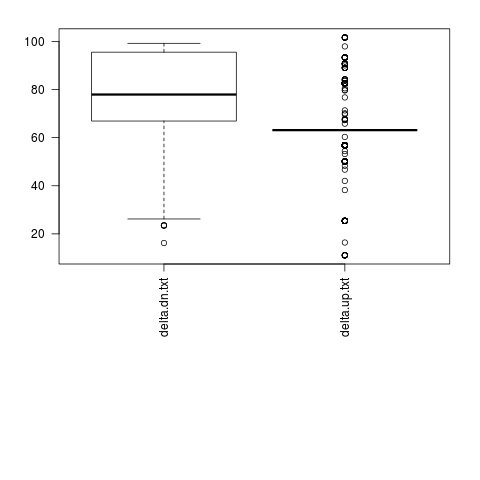

Supplement: S2 Dataset — (ZIP) [file pcbi.1010022.s006.zip › Final SPOROS output Figure 3/differential/ADnTPDvsCtrl/pvalue/miRNA/D_toxAnalysis.combined.miRNA.txt.box.png]

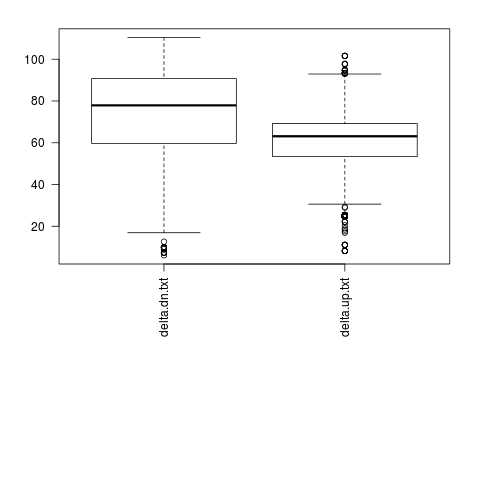

Supplement: S2 Dataset — (ZIP) [file pcbi.1010022.s006.zip › Final SPOROS output Figure 3/differential/ADnTPDvsCtrl/pvalue/sRNA/D_toxAnalysis.combined.sRNA.txt.box.png]

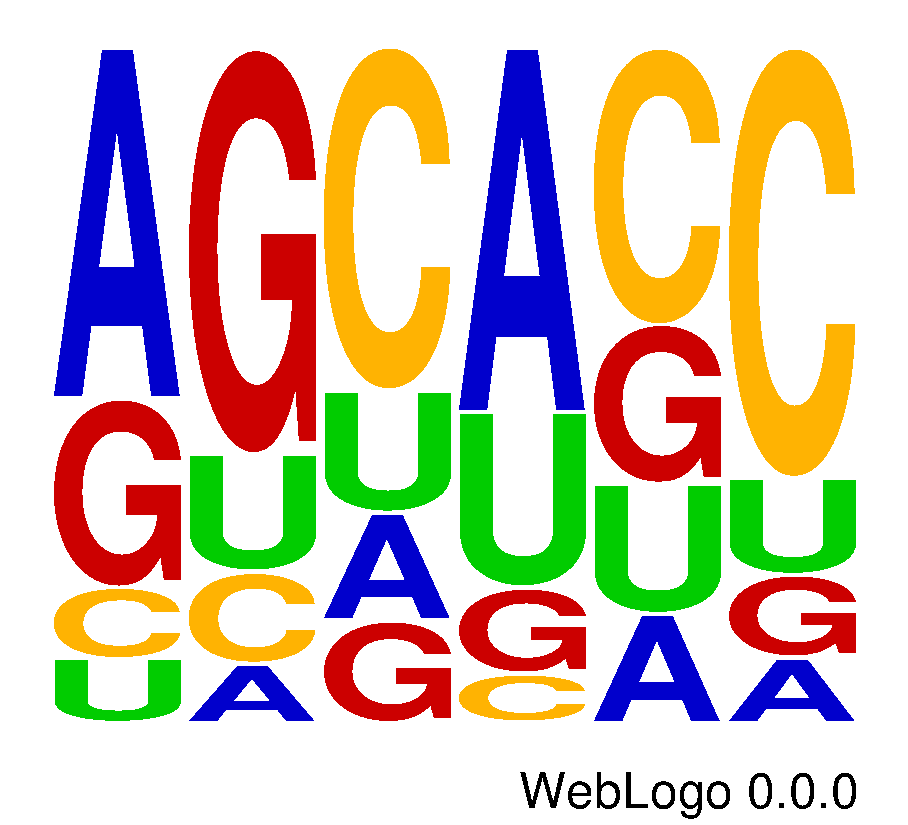

Supplement: S2 Dataset — (ZIP) [file pcbi.1010022.s006.zip › Final SPOROS output Figure 3/differential/ADnTPDvsCtrl/pvalue/sRNA/E_seedAnalysis.Delta.sRNA.up.png]

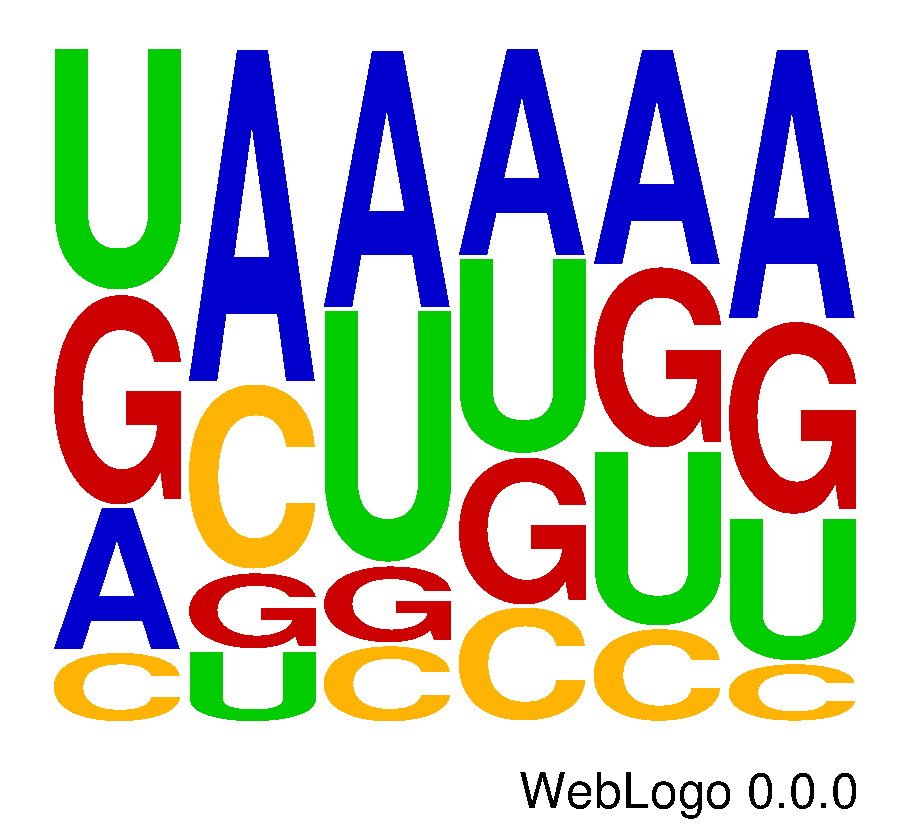

Supplement: S2 Dataset — (ZIP) [file pcbi.1010022.s006.zip › Final SPOROS output Figure 3/differential/ADnTPDvsCtrl/pvalue/sRNA/E_seedAnalysis.Delta.sRNA.dn.png]
