# Supplementary material for: SPOROS: A pipeline to analyze DISE/6mer seed toxicity
Source: PLoS Comput Biol. 2022 Mar 31;18(3):e1010022. doi: 10.1371/journal.pcbi.1010022 (PMC9004739; doi:10.1371/journal.pcbi.1010022)
Supplement: S1 Text — (PDF) [file pcbi.1010022.s004.pdf]

S1 Text. Script used to generate Fig 2A.

Authors: Alboukadel Kassambara, Fabian Mundt

Link to package:
https://CRAN.R-project.org/package=factoextra

description of use:
http://www.sthda.com/english/rpkgs/factoextra

R Script used for PCA plot in Fig 2A adapted from Statistical tools for high-throughput data analysis (http://www.sthda.com/english/wiki/fviz-pca-quick-principal-component-analysis-data-visualization-r-software-and-data-mining)

```
library(tidyverse)
library(data.table)
library(factoextra)

setwd("/Users/api/Desktop/DISE42_for methods")

#read in data
DISE42 <- fread("DISE-
042.noAdapter.notUMId.normCounts.table.withTox.withMiRNAandRNAworld.blast.trunc.minSu
m6.txt")

# make new dataframe with just the data from uninfected cells
DISE42_Uninfected <- cbind(DISE42[,1:2], DISE42[,4])
DISE42_Uninfected <- cbind(DISE42_Uninfected, DISE42[,10:11])
DISE42_Uninfected <- cbind(DISE42_Uninfected, DISE42[,16:17])
DISE42_Uninfected <- cbind(DISE42_Uninfected, DISE42[,22:23])
DISE42_Uninfected <- cbind(DISE42_Uninfected, DISE42[,32])

# abbreviate the column names
colnames(DISE42_Uninfected) <- c("Read", "Seed", "Avg_viability", "DicerKO.rep1",
"DicerKO.rep2", "DroshaKO.rep1", "DroshaKO.rep2", "WT.rep1", "WT.rep2", "RNAworld")

# This code splits the RNAworld column by the symbols | or _ and takes the first element to
extract
# just the RNA name from the alignment result
DISE42_RNAworld <- lapply(strsplit(DISE42_Uninfected$'RNAworld', "[|_]"), "[", 1)
DISE42_RNAworld <- as.data.frame(unlist(DISE42_RNAworld))
DISE42_Uninfected <- cbind(DISE42_Uninfected, DISE42_RNAworld)

# Add the read counts for reads mapping to the same RNA
DISE42_Uninfected_agg <- aggregate(DISE42_Uninfected[,4:9], by =
list(DISE42_Uninfected$`unlist(DISE42_RNAworld)`),
                        FUN = sum, na.rm = TRUE)
# changes all NAs to 0
DISE42_Uninfected_agg[is.na(DISE42_Uninfected_agg)] <- 0

# makes a new dataframe for the PCA analysis
```

```
DISE42_Uninfected_agg.names <- DISE42_Uninfected_agg$Group.1
rownames(DISE42_Uninfected_agg) <- DISE42_Uninfected_agg.names
DISE42_Uninfected_agg2 <- DISE42_Uninfected_agg[,2:7]

# compute PCA and save the results in DISE42_Uninfected_agg.pca
DISE42_Uninfected_agg.pca <- prcomp(DISE42_Uninfected_agg2, scale = TRUE)

# visualize the PCA
# how many dimensions explain the most variation
fviz_eig(DISE42_Uninfected_agg.pca)

# which RNAs contribute most to the variation
fviz_pca_ind(DISE42_Uninfected_agg.pca,
             col.ind = "cos2", # Color by the quality of representation
             gradient.cols = c("#00AFBB", "#E7B800", "#FC4E07"),
             repel = FALSE     # Avoid text overlapping
)

# how do the samples group
fviz_pca_var(DISE42_Uninfected_agg.pca, col.var="steelblue")+
  theme_minimal()

fviz_pca_var(DISE42_Uninfected_agg.pca, geom = c("point", "text"), col.var="black", repel =
TRUE)+
  theme_minimal()

fviz_pca_var(DISE42_Uninfected_agg.pca, geom = c("point", "text"), col.var = "contrib",
gradient.cols = c("#00AFBB", "#E7B800", "#FC4E07"), repel = TRUE)

library("corrplot")
var <- get_pca_var(DISE42_Uninfected_agg.pca)
corrplot(var$cos2, is.corr = FALSE)


# how do the samples group
fviz_pca_var(DISE42_Uninfected_agg.pca,
             col.var = "contrib", # Color by contributions to the PC
             gradient.cols = c("#00AFBB", "#E7B800", "#FC4E07"),
             repel = TRUE     # Avoid text overlapping
)

# overlay the RNA and sample grouping results
fviz_pca_biplot(DISE42_Uninfected_agg.pca, repel = FALSE,
                col.var = "#2E9FDF", # Variables color
                col.ind = "#696969"  # Individuals color
)
```
