# Supplementary material for: SPOROS: A pipeline to analyze DISE/6mer seed toxicity
Source: PLoS Comput Biol. 2022 Mar 31;18(3):e1010022. doi: 10.1371/journal.pcbi.1010022 (PMC9004739; doi:10.1371/journal.pcbi.1010022)
Supplement: S3 Dataset — (ZIP) [file pcbi.1010022.s007.zip › Suppl. Dataset 3.pdf]

SPOROS pipeline analysis of seed composition data using multinomial mixed models

1 Read in Output F from GitHub

```
> ## read in raw data from GitHub
> f.dicer <- "https://raw.githubusercontent.com/ebartom/SPOROS/main/Figure2.SPO
ROSpaper/totalCounts/sRNA/F_seedExpand.DicerKO.avg.sRNA.Figure2.txt"
> f.drosha <- "https://raw.githubusercontent.com/ebartom/SPOROS/main/Figure2.SP
OROSpaper/totalCounts/sRNA/F_seedExpand.DroshaKO.avg.sRNA.Figure2.txt"
> f.wildtype <- "https://raw.githubusercontent.com/ebartom/SPOROS/main/Figure2.
SPOROSpaper/totalCounts/sRNA/F_seedExpand.Wildtype.avg.sRNA.Figure2.txt"
>
> dicer <- read.table(f.dicer, header=TRUE, sep="\t")
> drosha <- read.table(f.drosha, header=TRUE, sep="\t")
> wildtype <- read.table(f.wildtype, header=TRUE, sep="\t")
```

There are 913 seeds in DicerKO data.

There are 889 seeds in DroshaKO data.

There are 951 seeds in Wildtype data.

There are 2753 seeds in the combined data set.

Example of each data set:

```
> # look at each file to check that data were read and combined correctly
> dicer %>% head
```

```
    Seed  Sample    SeedID Pos Base
1 AAAGUG DicerKO DicerKO.1   1    A
2 AAAGUG DicerKO DicerKO.1   2    A
3 AAAGUG DicerKO DicerKO.1   3    A
4 AAAGUG DicerKO DicerKO.1   4    G
5 AAAGUG DicerKO DicerKO.1   5    U
6 AAAGUG DicerKO DicerKO.1   6    G
```

```
> drosha %>% head
```

```
    Seed   Sample     SeedID Pos Base
1 AAAAGC DroshaKO DroshaKO.1   1    A
2 AAAAGC DroshaKO DroshaKO.1   2    A
3 AAAAGC DroshaKO DroshaKO.1   3    A
4 AAAAGC DroshaKO DroshaKO.1   4    A
5 AAAAGC DroshaKO DroshaKO.1   5    G
6 AAAAGC DroshaKO DroshaKO.1   6    C
```

```
> wildtype %>% head
```

```
    Seed   Sample     SeedID Pos Base
1 AAAGAA Wildtype Wildtype.1   1    A
2 AAAGAA Wildtype Wildtype.1   2    A
3 AAAGAA Wildtype Wildtype.1   3    A
4 AAAGAA Wildtype Wildtype.1   4    G
5 AAAGAA Wildtype Wildtype.1   5    A
6 AAAGAA Wildtype Wildtype.1   6    A
```

```
> dataCombined %>% head
```

```
    Seed  Sample    SeedID Pos Base
1 AAAGUG DicerKO DicerKO.1   1    A
2 AAAGUG DicerKO DicerKO.1   2    A
3 AAAGUG DicerKO DicerKO.1   3    A
4 AAAGUG DicerKO DicerKO.1   4    G
5 AAAGUG DicerKO DicerKO.1   5    U
6 AAAGUG DicerKO DicerKO.1   6    G
```

```
> dataCombined %>% tail
```

```
          Seed   Sample       SeedID Pos Base
   16513 UUGGCA Wildtype Wildtype.951   1    U
   16514 UUGGCA Wildtype Wildtype.951   2    U
   16515 UUGGCA Wildtype Wildtype.951   3    G
   16516 UUGGCA Wildtype Wildtype.951   4    G
   16517 UUGGCA Wildtype Wildtype.951   5    C
   16518 UUGGCA Wildtype Wildtype.951   6    A
```

Do the counts match?

```
> # check total seeds/id's
> (nrow(dicer)/6 + nrow(drosha)/6 + nrow(wildtype)/6 ==
+    length(unique(dataCombined$SeedID)))
```

```
   [1] TRUE
```

2 Save the data

Data saved for SAS analyses into:

```
   [1] "G:\\PeterM_XXX\\Analysis\\Data\\MethodsPaper\\data.seedLong.2021-08-12.
csv"
```

3 SAS Analysis using PROC GLIMMIX

```
> * update the path in the INFILE statement to match datapath above;
+ * note that sas uses single backslash in paths, e.g. "C:\sasdata";
+
+ data seedlong;
+ informat seed $6. sample $20. seedid $30.;
+ infile "G:\\PeterM_XXX\\Analysis\\Data\\MethodsPaper\\data.seedLong.2021-08-1
2.csv"
+   dlm="," firstobs=2;
+ input Seed $ sample $ seedid $ pos base $ ;
+ run;
+
+ proc glimmix data=seedlong outdesign = xx method=rmpl;
+ class seed seedid sample(ref="Wildtype") pos base(ref="A");
+ model base = sample pos sample*pos /dist=multinomial link=glogit s or(label)
ddfm=bw;
+ random intercept/ subject = seedid group=base type=chol;
+ store gmxres;
+ run;
```

Model Information

**Data Set**	WORK.SEEDLONG
**Response Variable**	base
**Response Distribution**	Multinomial (nominal)
**Link Function**	Generalized Logit
**Variance Function**	Default
**Variance Matrix Blocked By**	seedid
**Estimation Technique**	Residual MPL
**Degrees of Freedom Method**	Between-Within

Class Level Information

Class	Levels	Values

**seed**	240	AAAAGC AAAGAA AAAGCA AAAGCU AAAGUG AACACC AACACU AACCGG AACGGA AAGCUG AAGGUG AAUACU AAUCCC ACAGUA ACAUUC ACCCCA ACCCGG ACCCGU ACCCUG ACCGAG ACCGCC ACCGGG ACCUCC ACCUCG ACCUGG ACGCCU ACGCGA ACUCUG ACUGCU ACUGGC AGCACC AGCAGC AGCCGU AGCCUG AGCGAG AGCUGC AGCUGG AGCUUA AGGAGC AGGUAG AGUACG AGUGCA AGUUUC AUAUAU AUCCAG AUCCCA AUCCCC AUCCGG AUCGGG AUGACA AUGCCU AUGGCA AUGGCG AUGGCU AUUAUU AUUGCA CAAAAC CAAAGU CAAGAG CACAGU CACCAG CACCGC CACCGG CACCUG CACUGC CAGGCU CAGUGC CAUAGC CCACCC CCACGG CCAGCU CCAGGA CCCACA CCCACC CCCACU CCCAUA CCCCAC CCCCAG CCCCCA CCCCGA CCCCGC CCCCGG CCCCGU CCCCUG CCCGCA CCCGCG CCCGGA CCCGGC CCCGGG CCCUGA CCCUGC CCCUGG CCCUGU CCGAGU CCGCCG CCGCGG CCGGAG CCGGCG CCGGCU CCGGGC CCGGGU CCUCAC CCUCGU CCUCUU CCUGCC CCUGCU CCUGGC CCUGGU CGAAAC CGAAUC CGACCG CGAGGA CGAUUC CGCACU CGCCGC CGCCUG CGCGAC CGCGGG CGGAGG CGGCGG CGGGCG CGGGUC CGGGUG CGUACC CGUACG CGUAUC CUACAU CUAUGC CUCACC CUCAUG CUCGCU CUCGGC CUCGGU CUCUCG CUGACU CUGAUU CUGCAG CUGCCC CUGGAC CUGGUC CUGGUU CUUUGG GAACAG GAGCCA GAGGGG GAGGGU GAGGUA GCACCA GCAGAG GCAGCA GCAUCC GCCCGC GCCGCG GCCGUG GCCUGG GCGAGG GCGCGU GCGGGC GCGGGG GCGGGU GCUACA GCUAUG GCUCAG GCUCGA GCUCGG GCUGGU GCUUAU GGAAGA GGAGAG GGCAAG GGCAGU GGCCGA GGCGGC GGCUCA GGCUGG GGGCUG GGGGCC GGGUCG GGUACG GGUGCG GUAAAC GUACCG GUAGCG GUAGUG GUGCAA GUGCGC GUUGUA GUUUCU UAAGCC UAAUAC UACAGU UAGACU UAGAGG UAGCAC UAGCGA UAGCUU UAUAAU UAUACG UAUCAG UCAAGU UCACAG UCACAU UCACCA UCACCU UCAGGA UCAUGG UCCAGC UCCAGU UCCCAC UCCCCG UCCCCU UCCCGG UCCCUC UCCCUG UCCGAG UCCGGC UCCGGG UCCUGC UCGCUU UCGGCG UCUAAA UCUCAC UCUCGC UCUCGG UCUUUG UGCAUA UGCAUU UGCCGC UGCGCA UGGUCC UGUACG UGUCUG UGUGCG UUCCCG UUCCGG UUCGAU UUCUCA UUGCAC UUGGCA UUUCCG
**seedid**	2753	not printed
**sample**	3	DicerKO DroshaKO Wildtype
**pos**	6	1 2 3 4 5 6
**base**	4	A C G U

**Number of Observations Read**	16518

Number of Observations Used	16518

Response Profile

Ordered Value	base	Total Frequency
1	A	3204
2	C	6355
3	G	4285
4	U	2674

In modeling category probabilities, base='A' serves as the reference category.

Dimensions

G-side Cov. Parameters	4
Columns in X	84
Columns in Z per Subject	4
Subjects (Blocks in V)	2753
Max Obs per Subject	6

Optimization Information

Optimization Technique	Dual Quasi-Newton
Parameters in Optimization	4
Equality Constraints	1

**Lower Boundaries**	4
**Upper Boundaries**	1
**Fixed Effects**	Profiled
**Starting From**	Data

Iteration History

Iteration	Restarts	Subiterations	Objective Function	Change	Max Gradient
**0**	**0**	**7**	188693.55234	2.00000000	0.037381
**1**	**0**	**3**	189090.22873	2.00000000	0.000023
**2**	**0**	**2**	191080.04613	2.00000000	0.009061
**3**	**0**	**2**	191279.13083	0.23401438	3.222E-8
**4**	**0**	**2**	191283.77184	0.00009077	3.624E-8
**5**	**0**	**0**	191283.77528	0.00000000	3.09E-6

Convergence criterion (PCONV=1.11022E-8) satisfied.

Estimated G matrix is not positive definite.

Fit Statistics

**-2 Res Log Pseudo-Likelihood**	191283.8

Covariance Parameter Estimates

Cov Parm	Subject	Group	Estimate	Standard Error
CHOL(1,1)	seedid	base A	0.5000	.
CHOL(1,1)	seedid	base C	0.4217	0.03410
CHOL(1,1)	seedid	base G	0	.
CHOL(1,1)	seedid	base U	0	.

Solutions for Fixed Effects

Effect	base	sample	pos	Estimate	Standard Error	DF	t Value	Pr > |t|
Intercept	C			-1.3128	0.09827	2729	-13.36	<.0001
Intercept	G			-1.1292	0.09066	2729	-12.46	<.0001
Intercept	U			-1.1480	0.09131	2729	-12.57	<.0001
sample	C	DicerKO		1.7684	0.1341	2729	13.19	<.0001
sample	G	DicerKO		1.4992	0.1290	2729	11.63	<.0001
sample	U	DicerKO		0.4891	0.1514	2729	3.23	0.0013
sample	C	DroshaKO		2.9584	0.2031	2729	14.57	<.0001
sample	G	DroshaKO		3.5761	0.1919	2729	18.64	<.0001
sample	U	DroshaKO		2.8811	0.1983	2729	14.53	<.0001
sample	C	Wildtype		0	.	.	.	.
sample	G	Wildtype		0	.	.	.	.
sample	U	Wildtype		0	.	.	.	.
pos	C		1	-0.9188	0.1731	2729	-5.31	<.0001
pos	G		1	0.6124	0.1164	2729	5.26	<.0001

**pos**	**U**		**1**	-0.5293	0.1448	2729	-3.66	0.0003
**pos**	**C**		**2**	1.3850	0.1341	2729	10.33	<.0001
**pos**	**G**		**2**	1.6704	0.1232	2729	13.55	<.0001
**pos**	**U**		**2**	0.2229	0.1544	2729	1.44	0.1491
**pos**	**C**		**3**	2.2700	0.1315	2729	17.26	<.0001
**pos**	**G**		**3**	0.9229	0.1443	2729	6.40	<.0001
**pos**	**U**		**3**	1.1018	0.1411	2729	7.81	<.0001
**pos**	**C**		**4**	0.3252	0.1415	2729	2.30	0.0216
**pos**	**G**		**4**	0.6586	0.1252	2729	5.26	<.0001
**pos**	**U**		**4**	0.8303	0.1230	2729	6.75	<.0001
**pos**	**C**		**5**	1.9927	0.1392	2729	14.31	<.0001
**pos**	**G**		**5**	1.0889	0.1471	2729	7.40	<.0001
**pos**	**U**		**5**	1.9906	0.1332	2729	14.94	<.0001
**pos**	**C**		**6**	0	.	.	.	.
**pos**	**G**		**6**	0	.	.	.	.
**pos**	**U**		**6**	0	.	.	.	.
**sample*pos**	**C**	**DicerKO**	**1**	1.1915	0.2115	13735	5.63	<.0001
**sample*pos**	**G**	**DicerKO**	**1**	-2.9601	0.2422	13735	-12.22	<.0001
**sample*pos**	**U**	**DicerKO**	**1**	1.0444	0.2125	13735	4.91	<.0001
**sample*pos**	**C**	**DicerKO**	**2**	1.5772	0.2703	13735	5.84	<.0001
**sample*pos**	**G**	**DicerKO**	**2**	0.004345	0.2737	13735	0.02	0.9873
**sample*pos**	**U**	**DicerKO**	**2**	1.2570	0.3223	13735	3.90	<.0001
**sample*pos**	**C**	**DicerKO**	**3**	0.3778	0.2389	13735	1.58	0.1139

**sample*pos**	**G**	**DicerKO**	**3**	-0.2250	0.2645	13735	-0.85	0.3948
**sample*pos**	**U**	**DicerKO**	**3**	-0.04753	0.2919	13735	-0.16	0.8707
**sample*pos**	**C**	**DicerKO**	**4**	0.8536	0.2047	13735	4.17	<.0001
**sample*pos**	**G**	**DicerKO**	**4**	-0.2423	0.2020	13735	-1.20	0.2305
**sample*pos**	**U**	**DicerKO**	**4**	0.6045	0.2157	13735	2.80	0.0051
**sample*pos**	**C**	**DicerKO**	**5**	-3.2896	0.2056	13735	-16.00	<.0001
**sample*pos**	**G**	**DicerKO**	**5**	-0.8736	0.1923	13735	-4.54	<.0001
**sample*pos**	**U**	**DicerKO**	**5**	-1.5167	0.2053	13735	-7.39	<.0001
**sample*pos**	**C**	**DicerKO**	**6**	0	.	.	.	.
**sample*pos**	**G**	**DicerKO**	**6**	0	.	.	.	.
**sample*pos**	**U**	**DicerKO**	**6**	0	.	.	.	.
**sample*pos**	**C**	**DroshaKO**	**1**	0.2040	0.2696	13735	0.76	0.4492
**sample*pos**	**G**	**DroshaKO**	**1**	-2.2260	0.2319	13735	-9.60	<.0001
**sample*pos**	**U**	**DroshaKO**	**1**	-0.8743	0.2567	13735	-3.41	0.0007
**sample*pos**	**C**	**DroshaKO**	**2**	-1.1516	0.2454	13735	-4.69	<.0001
**sample*pos**	**G**	**DroshaKO**	**2**	-4.8489	0.2699	13735	-17.97	<.0001
**sample*pos**	**U**	**DroshaKO**	**2**	-2.9818	0.3009	13735	-9.91	<.0001
**sample*pos**	**C**	**DroshaKO**	**3**	-2.1477	0.2460	13735	-8.73	<.0001
**sample*pos**	**G**	**DroshaKO**	**3**	-2.8291	0.2557	13735	-11.06	<.0001
**sample*pos**	**U**	**DroshaKO**	**3**	-3.7410	0.2932	13735	-12.76	<.0001
**sample*pos**	**C**	**DroshaKO**	**4**	0.06387	0.2823	13735	0.23	0.8210
**sample*pos**	**G**	**DroshaKO**	**4**	-1.2034	0.2702	13735	-4.45	<.0001
**sample*pos**	**U**	**DroshaKO**	**4**	-0.6354	0.2734	13735	-2.32	0.0201

**sample*pos**	**C**	**DroshaKO**	**5**	-2.8822	0.2559	13735	-11.26	<.0001
**sample*pos**	**G**	**DroshaKO**	**5**	-1.8644	0.2492	13735	-7.48	<.0001
**sample*pos**	**U**	**DroshaKO**	**5**	-4.5446	0.2854	13735	-15.92	<.0001
**sample*pos**	**C**	**DroshaKO**	**6**	0	.	.	.	.
**sample*pos**	**G**	**DroshaKO**	**6**	0	.	.	.	.
**sample*pos**	**U**	**DroshaKO**	**6**	0	.	.	.	.
**sample*pos**	**C**	**Wildtype**	**1**	0	.	.	.	.
**sample*pos**	**G**	**Wildtype**	**1**	0	.	.	.	.
**sample*pos**	**U**	**Wildtype**	**1**	0	.	.	.	.
**sample*pos**	**C**	**Wildtype**	**2**	0	.	.	.	.
**sample*pos**	**G**	**Wildtype**	**2**	0	.	.	.	.
**sample*pos**	**U**	**Wildtype**	**2**	0	.	.	.	.
**sample*pos**	**C**	**Wildtype**	**3**	0	.	.	.	.
**sample*pos**	**G**	**Wildtype**	**3**	0	.	.	.	.
**sample*pos**	**U**	**Wildtype**	**3**	0	.	.	.	.
**sample*pos**	**C**	**Wildtype**	**4**	0	.	.	.	.
**sample*pos**	**G**	**Wildtype**	**4**	0	.	.	.	.
**sample*pos**	**U**	**Wildtype**	**4**	0	.	.	.	.
**sample*pos**	**C**	**Wildtype**	**5**	0	.	.	.	.
**sample*pos**	**G**	**Wildtype**	**5**	0	.	.	.	.
**sample*pos**	**U**	**Wildtype**	**5**	0	.	.	.	.
**sample*pos**	**C**	**Wildtype**	**6**	0	.	.	.	.
**sample*pos**	**G**	**Wildtype**	**6**	0	.	.	.	.

**sample*pos**	**U**	**Wildtype**	**6**	0	.	.	.	.

Odds Ratio Estimates

Comparison	Estimate	DF	95% Confidence Limits	
**C: sample DicerKO vs Wildtype**	6.598	2729	5.696	7.643
**G: sample DicerKO vs Wildtype**	2.188	2729	1.881	2.546
**U: sample DicerKO vs Wildtype**	2.040	2729	1.743	2.387
**C: sample DroshaKO vs Wildtype**	7.191	2729	6.238	8.289
**G: sample DroshaKO vs Wildtype**	4.113	2729	3.581	4.724
**U: sample DroshaKO vs Wildtype**	2.120	2729	1.806	2.490
**C: pos 1 vs 6**	0.635	2729	0.524	0.771
**G: pos 1 vs 6**	0.327	2729	0.267	0.402
**U: pos 1 vs 6**	0.623	2729	0.512	0.759
**C: pos 2 vs 6**	4.604	2729	3.687	5.748
**G: pos 2 vs 6**	1.057	2729	0.833	1.341
**U: pos 2 vs 6**	0.703	2729	0.537	0.921
**C: pos 3 vs 6**	5.366	2729	4.362	6.600
**G: pos 3 vs 6**	0.909	2729	0.729	1.134
**U: pos 3 vs 6**	0.851	2729	0.660	1.098
**C: pos 4 vs 6**	1.880	2729	1.526	2.315
**G: pos 4 vs 6**	1.193	2729	0.972	1.464
**U: pos 4 vs 6**	2.270	2729	1.835	2.809
**C: pos 5 vs 6**	0.937	2729	0.772	1.138

**G: pos 5 vs 6**	1.193	2729	0.994	1.431
**U: pos 5 vs 6**	0.971	2729	0.785	1.201

Type III Tests of Fixed Effects

Effect	Num DF	Den DF	F Value	Pr > F
**sample**	6	2729	189.90	<.0001
**pos**	15	2729	138.39	<.0001
**sample*pos**	30	13735	56.61	<.0001

```
>
+ proc plm restore=gmxres noclprint plots=none;
+ lsmeans sample/ilink oddsratio adj=tukey cl e;
+ slice sample*pos/sliceby=pos diff oddsratio adj=tukey cl;
+ ods output slicediffs= sampleposdiffs diffs= samplediffs;
+ run;
```

```
> proc sort data=sampleposdiffs;
+ by sample slice base;
+
+ proc print data=sampleposdiffs;
+ where _sample = "Wildtype";
+ var sample _sample slice base OddsRatio AdjLowerOR AdjUpperOR Adjp;
+ run;
```

Obs	sample	_sample	Slice	base	OddsRatio	AdjLowerOR	AdjUpperOR	Adjp
**2**	DicerKO	Wildtype	pos 1	C	19.295	13.080	28.463	<.0001
**4**	DicerKO	Wildtype	pos 1	G	0.232	0.143	0.375	<.0001
**6**	DicerKO	Wildtype	pos 1	U	4.634	3.267	6.574	<.0001
**8**	DicerKO	Wildtype	pos 2	C	28.375	16.308	49.370	<.0001

**10**	DicerKO	Wildtype	pos 2	G	4.498	2.554	7.921	<.0001
**12**	DicerKO	Wildtype	pos 2	U	5.732	2.942	11.169	<.0001
**14**	DicerKO	Wildtype	pos 3	C	8.552	5.355	13.655	<.0001
**16**	DicerKO	Wildtype	pos 3	G	3.576	2.081	6.143	<.0001
**18**	DicerKO	Wildtype	pos 3	U	1.555	0.866	2.791	0.1800
**20**	DicerKO	Wildtype	pos 4	C	13.763	9.523	19.889	<.0001
**22**	DicerKO	Wildtype	pos 4	G	3.515	2.441	5.060	<.0001
**24**	DicerKO	Wildtype	pos 4	U	2.985	2.083	4.278	<.0001
**26**	DicerKO	Wildtype	pos 5	C	0.218	0.151	0.317	<.0001
**28**	DicerKO	Wildtype	pos 5	G	1.869	1.338	2.612	<.0001
**30**	DicerKO	Wildtype	pos 5	U	0.358	0.259	0.495	<.0001
**32**	DicerKO	Wildtype	pos 6	C	5.861	4.280	8.026	<.0001
**34**	DicerKO	Wildtype	pos 6	G	4.478	3.310	6.059	<.0001
**36**	DicerKO	Wildtype	pos 6	U	1.631	1.144	2.326	0.0036
**37**	DroshaKO	Wildtype	pos 1	C	23.628	15.512	35.989	<.0001
**38**	DroshaKO	Wildtype	pos 1	G	3.858	2.842	5.236	<.0001
**39**	DroshaKO	Wildtype	pos 1	U	7.439	5.076	10.904	<.0001
**40**	DroshaKO	Wildtype	pos 2	C	6.091	4.381	8.468	<.0001
**41**	DroshaKO	Wildtype	pos 2	G	0.280	0.179	0.437	<.0001
**42**	DroshaKO	Wildtype	pos 2	U	0.904	0.532	1.537	0.8967
**43**	DroshaKO	Wildtype	pos 3	C	2.249	1.614	3.134	<.0001
**44**	DroshaKO	Wildtype	pos 3	G	2.111	1.420	3.137	<.0001
**45**	DroshaKO	Wildtype	pos 3	U	0.423	0.255	0.702	0.0002

**46**	DroshaKO	Wildtype	pos 4	C	20.537	12.911	32.668	<.0001
**47**	DroshaKO	Wildtype	pos 4	G	10.726	6.868	16.753	<.0001
**48**	DroshaKO	Wildtype	pos 4	U	9.446	6.076	14.686	<.0001
**49**	DroshaKO	Wildtype	pos 5	C	1.079	0.745	1.563	0.8798
**50**	DroshaKO	Wildtype	pos 5	G	5.539	3.815	8.042	<.0001
**51**	DroshaKO	Wildtype	pos 5	U	0.189	0.117	0.307	<.0001
**52**	DroshaKO	Wildtype	pos 6	C	19.267	11.969	31.014	<.0001
**53**	DroshaKO	Wildtype	pos 6	G	35.734	22.791	56.027	<.0001
**54**	DroshaKO	Wildtype	pos 6	U	17.833	11.205	28.382	<.0001

```
> * update the path in the OUTFILE statement below to match datapath;
+ * note that sas uses single backslash for paths, e.g. "C:\sasdata";
+
+ proc export data=work.sampleposdiffs
+   outfile="G:\PeterM_XXX\Analysis\Data\MethodsPaper\F_OREstimates_Fig2_081221
.xlsx"
+   dbms=excel replace;
+   sheet="OR Position";
+ run;
```

4 Dicer vs. Wildtype

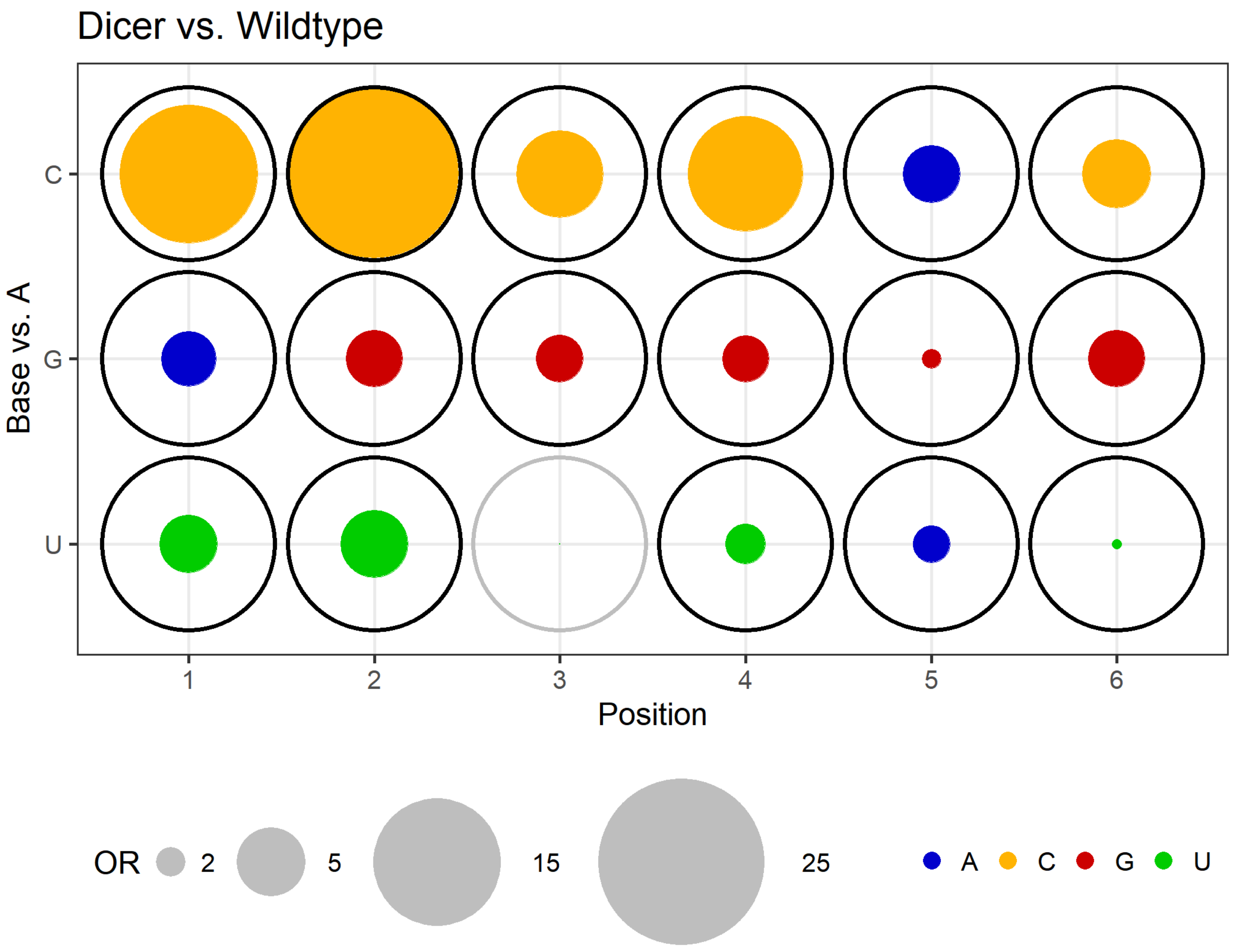

5 Drosha vs. Wildtype

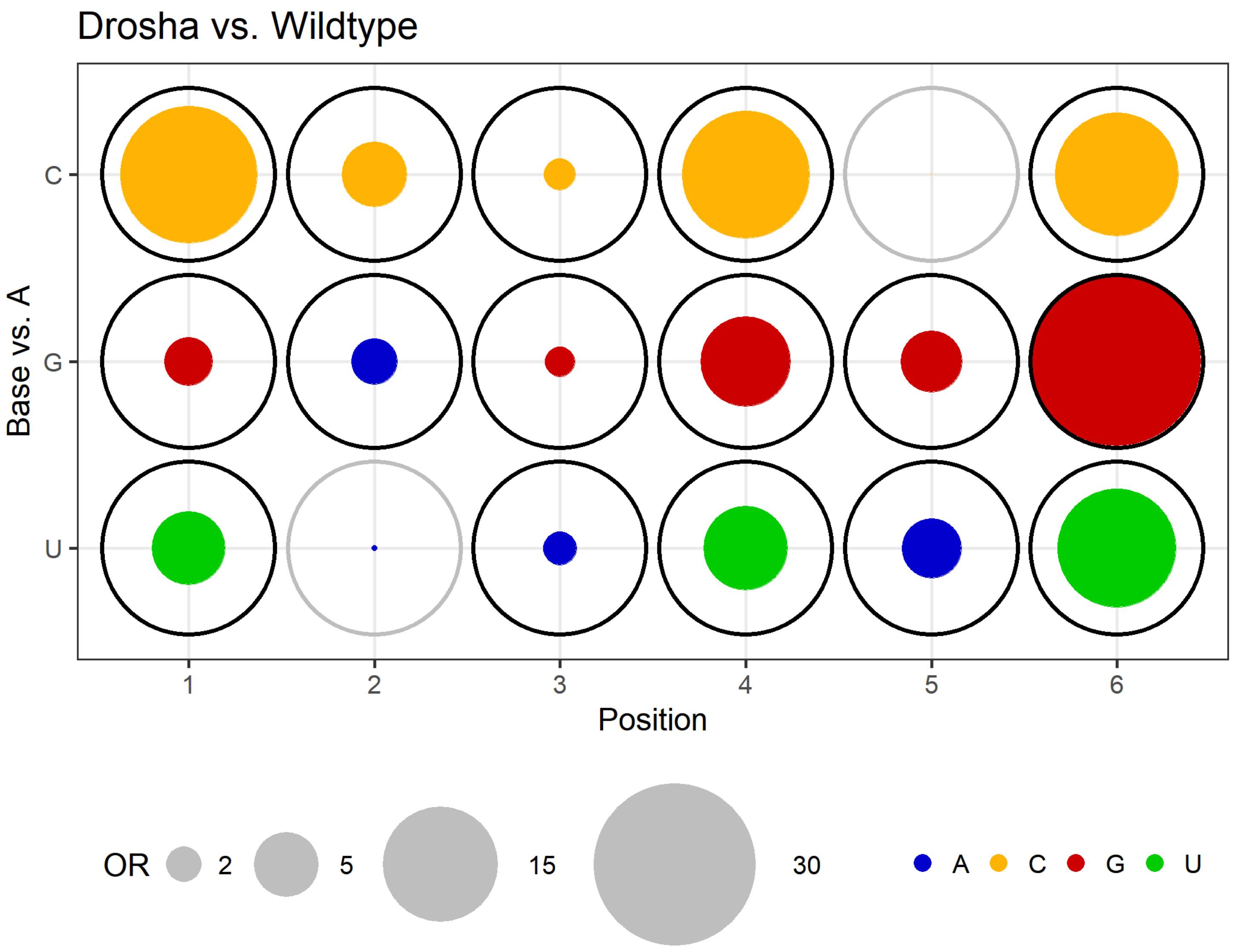
Drosha vs. Wildtype
Base vs. A
C
G
U
Position
1
2
3
4
5
6
OR
2
5
15
30
A
C
G
U
