# Supplementary material for: SPOROS: A pipeline to analyze DISE/6mer seed toxicity
Source: PLoS Comput Biol. 2022 Mar 31;18(3):e1010022. doi: 10.1371/journal.pcbi.1010022 (PMC9004739; doi:10.1371/journal.pcbi.1010022)
Supplement: S3 Dataset — (ZIP) [file pcbi.1010022.s007.zip › Suppl. Dataset 3_F_SPOROS_multmixed_analysis---Fig2---081221.html]

SPOROS pipeline analysis of seed composition data using multinomial mixed models


# SPOROS pipeline analysis of seed composition data using multinomial mixed models

# 1 Read in Output F from GitHub

```
> ## read in raw data from GitHub
> f.dicer <- "https://raw.githubusercontent.com/ebartom/SPOROS/main/Figure2.SPOROSpaper/totalCounts/sRNA/F_seedExpand.DicerKO.avg.sRNA.Figure2.txt"
> f.drosha <- "https://raw.githubusercontent.com/ebartom/SPOROS/main/Figure2.SPOROSpaper/totalCounts/sRNA/F_seedExpand.DroshaKO.avg.sRNA.Figure2.txt"
> f.wildtype <- "https://raw.githubusercontent.com/ebartom/SPOROS/main/Figure2.SPOROSpaper/totalCounts/sRNA/F_seedExpand.Wildtype.avg.sRNA.Figure2.txt"
> 
> dicer <- read.table(f.dicer, header=TRUE, sep="\t")
> drosha <- read.table(f.drosha, header=TRUE, sep="\t")
> wildtype <- read.table(f.wildtype, header=TRUE, sep="\t")
```

There are 913 seeds in DicerKO data.

There are 889 seeds in DroshaKO data.

There are 951 seeds in Wildtype data.

There are 2753 seeds in the combined data set.

Example of each data set:

```
> # look at each file to check that data were read and combined correctly
> dicer %>% head
```

```
       Seed  Sample    SeedID Pos Base
   1 AAAGUG DicerKO DicerKO.1   1    A
   2 AAAGUG DicerKO DicerKO.1   2    A
   3 AAAGUG DicerKO DicerKO.1   3    A
   4 AAAGUG DicerKO DicerKO.1   4    G
   5 AAAGUG DicerKO DicerKO.1   5    U
   6 AAAGUG DicerKO DicerKO.1   6    G
```

```
> drosha %>% head
```

```
       Seed   Sample     SeedID Pos Base
   1 AAAAGC DroshaKO DroshaKO.1   1    A
   2 AAAAGC DroshaKO DroshaKO.1   2    A
   3 AAAAGC DroshaKO DroshaKO.1   3    A
   4 AAAAGC DroshaKO DroshaKO.1   4    A
   5 AAAAGC DroshaKO DroshaKO.1   5    G
   6 AAAAGC DroshaKO DroshaKO.1   6    C
```

```
> wildtype %>% head
```

```
       Seed   Sample     SeedID Pos Base
   1 AAAGAA Wildtype Wildtype.1   1    A
   2 AAAGAA Wildtype Wildtype.1   2    A
   3 AAAGAA Wildtype Wildtype.1   3    A
   4 AAAGAA Wildtype Wildtype.1   4    G
   5 AAAGAA Wildtype Wildtype.1   5    A
   6 AAAGAA Wildtype Wildtype.1   6    A
```

```
> dataCombined %>% head
```

```
       Seed  Sample    SeedID Pos Base
   1 AAAGUG DicerKO DicerKO.1   1    A
   2 AAAGUG DicerKO DicerKO.1   2    A
   3 AAAGUG DicerKO DicerKO.1   3    A
   4 AAAGUG DicerKO DicerKO.1   4    G
   5 AAAGUG DicerKO DicerKO.1   5    U
   6 AAAGUG DicerKO DicerKO.1   6    G
```

```
> dataCombined %>% tail
```

```
           Seed   Sample       SeedID Pos Base
   16513 UUGGCA Wildtype Wildtype.951   1    U
   16514 UUGGCA Wildtype Wildtype.951   2    U
   16515 UUGGCA Wildtype Wildtype.951   3    G
   16516 UUGGCA Wildtype Wildtype.951   4    G
   16517 UUGGCA Wildtype Wildtype.951   5    C
   16518 UUGGCA Wildtype Wildtype.951   6    A
```

Do the counts match?

```
> # check total seeds/id's
> (nrow(dicer)/6 + nrow(drosha)/6 + nrow(wildtype)/6 ==
+    length(unique(dataCombined$SeedID)))
```

```
   [1] TRUE
```

# 2 Save the data

Data saved for SAS analyses into:

```
   [1] "G:\\PeterM_XXX\\Analysis\\Data\\MethodsPaper\\data.seedLong.2021-08-12.csv"
```

# 3 SAS Analysis using PROC GLIMMIX

```
> * update the path in the INFILE statement to match datapath above;
+ * note that sas uses single backslash in paths, e.g. "C:\sasdata";
+ 
+ data seedlong;
+ informat seed $6. sample $20. seedid $30.;
+ infile "G:\\PeterM_XXX\\Analysis\\Data\\MethodsPaper\\data.seedLong.2021-08-12.csv" 
+   dlm="," firstobs=2;
+ input Seed $ sample $ seedid $ pos base $ ;
+ run;
+ 
+ proc glimmix data=seedlong outdesign = xx method=rmpl;
+ class seed seedid sample(ref="Wildtype") pos base(ref="A");
+ model base = sample pos sample*pos /dist=multinomial link=glogit s or(label) ddfm=bw;
+ random intercept/ subject = seedid group=base type=chol;
+ store gmxres;
+ run;
```

| Model Information | |
| --- | --- |
| Data Set | WORK.SEEDLONG |
| Response Variable | base |
| Response Distribution | Multinomial (nominal) |
| Link Function | Generalized Logit |
| Variance Function | Default |
| Variance Matrix Blocked By | seedid |
| Estimation Technique | Residual MPL |
| Degrees of Freedom Method | Between-Within |

  
 

| Class Level Information | | |
| --- | --- | --- |
| Class | Levels | Values |
| seed | 240 | AAAAGC AAAGAA AAAGCA AAAGCU AAAGUG AACACC AACACU AACCGG AACGGA AAGCUG AAGGUG AAUACU AAUCCC ACAGUA ACAUUC ACCCCA ACCCGG ACCCGU ACCCUG ACCGAG ACCGCC ACCGGG ACCUCC ACCUCG ACCUGG ACGCCU ACGCGA ACUCUG ACUGCU ACUGGC AGCACC AGCAGC AGCCGU AGCCUG AGCGAG AGCUGC AGCUGG AGCUUA AGGAGC AGGUAG AGUACG AGUGCA AGUUUC AUAUAU AUCCAG AUCCCA AUCCCC AUCCGG AUCGGG AUGACA AUGCCU AUGGCA AUGGCG AUGGCU AUUAUU AUUGCA CAAAAC CAAAGU CAAGAG CACAGU CACCAG CACCGC CACCGG CACCUG CACUGC CAGGCU CAGUGC CAUAGC CCACCC CCACGG CCAGCU CCAGGA CCCACA CCCACC CCCACU CCCAUA CCCCAC CCCCAG CCCCCA CCCCGA CCCCGC CCCCGG CCCCGU CCCCUG CCCGCA CCCGCG CCCGGA CCCGGC CCCGGG CCCUGA CCCUGC CCCUGG CCCUGU CCGAGU CCGCCG CCGCGG CCGGAG CCGGCG CCGGCU CCGGGC CCGGGU CCUCAC CCUCGU CCUCUU CCUGCC CCUGCU CCUGGC CCUGGU CGAAAC CGAAUC CGACCG CGAGGA CGAUUC CGCACU CGCCGC CGCCUG CGCGAC CGCGGG CGGAGG CGGCGG CGGGCG CGGGUC CGGGUG CGUACC CGUACG CGUAUC CUACAU CUAUGC CUCACC CUCAUG CUCGCU CUCGGC CUCGGU CUCUCG CUGACU CUGAUU CUGCAG CUGCCC CUGGAC CUGGUC CUGGUU CUUUGG GAACAG GAGCCA GAGGGG GAGGGU GAGGUA GCACCA GCAGAG GCAGCA GCAUCC GCCCGC GCCGCG GCCGUG GCCUGG GCGAGG GCGCGU GCGGGC GCGGGG GCGGGU GCUACA GCUAUG GCUCAG GCUCGA GCUCGG GCUGGU GCUUAU GGAAGA GGAGAG GGCAAG GGCAGU GGCCGA GGCGGC GGCUCA GGCUGG GGGCUG GGGGCC GGGUCG GGUACG GGUGCG GUAAAC GUACCG GUAGCG GUAGUG GUGCAA GUGCGC GUUGUA GUUUCU UAAGCC UAAUAC UACAGU UAGACU UAGAGG UAGCAC UAGCGA UAGCUU UAUAAU UAUACG UAUCAG UCAAGU UCACAG UCACAU UCACCA UCACCU UCAGGA UCAUGG UCCAGC UCCAGU UCCCAC UCCCCG UCCCCU UCCCGG UCCCUC UCCCUG UCCGAG UCCGGC UCCGGG UCCUGC UCGCUU UCGGCG UCUAAA UCUCAC UCUCGC UCUCGG UCUUUG UGCAUA UGCAUU UGCCGC UGCGCA UGGUCC UGUACG UGUCUG UGUGCG UUCCCG UUCCGG UUCGAU UUCUCA UUGCAC UUGGCA UUUCCG |
| seedid | 2753 | not printed |
| sample | 3 | DicerKO DroshaKO Wildtype |
| pos | 6 | 1 2 3 4 5 6 |
| base | 4 | A C G U |

  
 

|  |  |
| --- | --- |
| Number of Observations Read | 16518 |
| Number of Observations Used | 16518 |

  
 

| Response Profile | | |
| --- | --- | --- |
| Ordered | base | Total |
| 1 | A | 3204 |
| 2 | C | 6355 |
| 3 | G | 4285 |
| 4 | U | 2674 |
| In modeling category probabilities, | | |
| --- | --- | --- |

  
 

| Dimensions | |
| --- | --- |
| G-side Cov. Parameters | 4 |
| Columns in X | 84 |
| Columns in Z per Subject | 4 |
| Subjects (Blocks in V) | 2753 |
| Max Obs per Subject | 6 |

  
 

| Optimization Information | |
| --- | --- |
| Optimization Technique | Dual Quasi-Newton |
| Parameters in Optimization | 4 |
| Equality Constraints | 1 |
| Lower Boundaries | 4 |
| Upper Boundaries | 1 |
| Fixed Effects | Profiled |
| Starting From | Data |

  
 

| Iteration History | | | | | |
| --- | --- | --- | --- | --- | --- |
| Iteration | Restarts | Subiterations | Objective | Change | Max |
| 0 | 0 | 7 | 188693.55234 | 2.00000000 | 0.037381 |
| 1 | 0 | 3 | 189090.22873 | 2.00000000 | 0.000023 |
| 2 | 0 | 2 | 191080.04613 | 2.00000000 | 0.009061 |
| 3 | 0 | 2 | 191279.13083 | 0.23401438 | 3.222E-8 |
| 4 | 0 | 2 | 191283.77184 | 0.00009077 | 3.624E-8 |
| 5 | 0 | 0 | 191283.77528 | 0.00000000 | 3.09E-6 |

  
 

|  |
| --- |
| Convergence criterion (PCONV=1.11022E-8) satisfied. |

  
   

|  |
| --- |
| Estimated G matrix is not positive definite. |

| Fit Statistics | |
| --- | --- |
| -2 Res Log Pseudo-Likelihood | 191283.8 |

  
 

| Covariance Parameter Estimates | | | | |
| --- | --- | --- | --- | --- |
| Cov Parm | Subject | Group | Estimate | Standard |
| CHOL(1,1) | seedid | base A | 0.5000 | . |
| CHOL(1,1) | seedid | base C | 0.4217 | 0.03410 |
| CHOL(1,1) | seedid | base G | 0 | . |
| CHOL(1,1) | seedid | base U | 0 | . |

  
 

| Solutions for Fixed Effects | | | | | | | | |
| --- | --- | --- | --- | --- | --- | --- | --- | --- |
| Effect | base | sample | pos | Estimate | Standard | DF | t Value | Pr > |t| |
| Intercept | C |  |  | -1.3128 | 0.09827 | 2729 | -13.36 | <.0001 |
| Intercept | G |  |  | -1.1292 | 0.09066 | 2729 | -12.46 | <.0001 |
| Intercept | U |  |  | -1.1480 | 0.09131 | 2729 | -12.57 | <.0001 |
| sample | C | DicerKO |  | 1.7684 | 0.1341 | 2729 | 13.19 | <.0001 |
| sample | G | DicerKO |  | 1.4992 | 0.1290 | 2729 | 11.63 | <.0001 |
| sample | U | DicerKO |  | 0.4891 | 0.1514 | 2729 | 3.23 | 0.0013 |
| sample | C | DroshaKO |  | 2.9584 | 0.2031 | 2729 | 14.57 | <.0001 |
| sample | G | DroshaKO |  | 3.5761 | 0.1919 | 2729 | 18.64 | <.0001 |
| sample | U | DroshaKO |  | 2.8811 | 0.1983 | 2729 | 14.53 | <.0001 |
| sample | C | Wildtype |  | 0 | . | . | . | . |
| sample | G | Wildtype |  | 0 | . | . | . | . |
| sample | U | Wildtype |  | 0 | . | . | . | . |
| pos | C |  | 1 | -0.9188 | 0.1731 | 2729 | -5.31 | <.0001 |
| pos | G |  | 1 | 0.6124 | 0.1164 | 2729 | 5.26 | <.0001 |
| pos | U |  | 1 | -0.5293 | 0.1448 | 2729 | -3.66 | 0.0003 |
| pos | C |  | 2 | 1.3850 | 0.1341 | 2729 | 10.33 | <.0001 |
| pos | G |  | 2 | 1.6704 | 0.1232 | 2729 | 13.55 | <.0001 |
| pos | U |  | 2 | 0.2229 | 0.1544 | 2729 | 1.44 | 0.1491 |
| pos | C |  | 3 | 2.2700 | 0.1315 | 2729 | 17.26 | <.0001 |
| pos | G |  | 3 | 0.9229 | 0.1443 | 2729 | 6.40 | <.0001 |
| pos | U |  | 3 | 1.1018 | 0.1411 | 2729 | 7.81 | <.0001 |
| pos | C |  | 4 | 0.3252 | 0.1415 | 2729 | 2.30 | 0.0216 |
| pos | G |  | 4 | 0.6586 | 0.1252 | 2729 | 5.26 | <.0001 |
| pos | U |  | 4 | 0.8303 | 0.1230 | 2729 | 6.75 | <.0001 |
| pos | C |  | 5 | 1.9927 | 0.1392 | 2729 | 14.31 | <.0001 |
| pos | G |  | 5 | 1.0889 | 0.1471 | 2729 | 7.40 | <.0001 |
| pos | U |  | 5 | 1.9906 | 0.1332 | 2729 | 14.94 | <.0001 |
| pos | C |  | 6 | 0 | . | . | . | . |
| pos | G |  | 6 | 0 | . | . | . | . |
| pos | U |  | 6 | 0 | . | . | . | . |
| sample\*pos | C | DicerKO | 1 | 1.1915 | 0.2115 | 13735 | 5.63 | <.0001 |
| sample\*pos | G | DicerKO | 1 | -2.9601 | 0.2422 | 13735 | -12.22 | <.0001 |
| sample\*pos | U | DicerKO | 1 | 1.0444 | 0.2125 | 13735 | 4.91 | <.0001 |
| sample\*pos | C | DicerKO | 2 | 1.5772 | 0.2703 | 13735 | 5.84 | <.0001 |
| sample\*pos | G | DicerKO | 2 | 0.004345 | 0.2737 | 13735 | 0.02 | 0.9873 |
| sample\*pos | U | DicerKO | 2 | 1.2570 | 0.3223 | 13735 | 3.90 | <.0001 |
| sample\*pos | C | DicerKO | 3 | 0.3778 | 0.2389 | 13735 | 1.58 | 0.1139 |
| sample\*pos | G | DicerKO | 3 | -0.2250 | 0.2645 | 13735 | -0.85 | 0.3948 |
| sample\*pos | U | DicerKO | 3 | -0.04753 | 0.2919 | 13735 | -0.16 | 0.8707 |
| sample\*pos | C | DicerKO | 4 | 0.8536 | 0.2047 | 13735 | 4.17 | <.0001 |
| sample\*pos | G | DicerKO | 4 | -0.2423 | 0.2020 | 13735 | -1.20 | 0.2305 |
| sample\*pos | U | DicerKO | 4 | 0.6045 | 0.2157 | 13735 | 2.80 | 0.0051 |
| sample\*pos | C | DicerKO | 5 | -3.2896 | 0.2056 | 13735 | -16.00 | <.0001 |
| sample\*pos | G | DicerKO | 5 | -0.8736 | 0.1923 | 13735 | -4.54 | <.0001 |
| sample\*pos | U | DicerKO | 5 | -1.5167 | 0.2053 | 13735 | -7.39 | <.0001 |
| sample\*pos | C | DicerKO | 6 | 0 | . | . | . | . |
| sample\*pos | G | DicerKO | 6 | 0 | . | . | . | . |
| sample\*pos | U | DicerKO | 6 | 0 | . | . | . | . |
| sample\*pos | C | DroshaKO | 1 | 0.2040 | 0.2696 | 13735 | 0.76 | 0.4492 |
| sample\*pos | G | DroshaKO | 1 | -2.2260 | 0.2319 | 13735 | -9.60 | <.0001 |
| sample\*pos | U | DroshaKO | 1 | -0.8743 | 0.2567 | 13735 | -3.41 | 0.0007 |
| sample\*pos | C | DroshaKO | 2 | -1.1516 | 0.2454 | 13735 | -4.69 | <.0001 |
| sample\*pos | G | DroshaKO | 2 | -4.8489 | 0.2699 | 13735 | -17.97 | <.0001 |
| sample\*pos | U | DroshaKO | 2 | -2.9818 | 0.3009 | 13735 | -9.91 | <.0001 |
| sample\*pos | C | DroshaKO | 3 | -2.1477 | 0.2460 | 13735 | -8.73 | <.0001 |
| sample\*pos | G | DroshaKO | 3 | -2.8291 | 0.2557 | 13735 | -11.06 | <.0001 |
| sample\*pos | U | DroshaKO | 3 | -3.7410 | 0.2932 | 13735 | -12.76 | <.0001 |
| sample\*pos | C | DroshaKO | 4 | 0.06387 | 0.2823 | 13735 | 0.23 | 0.8210 |
| sample\*pos | G | DroshaKO | 4 | -1.2034 | 0.2702 | 13735 | -4.45 | <.0001 |
| sample\*pos | U | DroshaKO | 4 | -0.6354 | 0.2734 | 13735 | -2.32 | 0.0201 |
| sample\*pos | C | DroshaKO | 5 | -2.8822 | 0.2559 | 13735 | -11.26 | <.0001 |
| sample\*pos | G | DroshaKO | 5 | -1.8644 | 0.2492 | 13735 | -7.48 | <.0001 |
| sample\*pos | U | DroshaKO | 5 | -4.5446 | 0.2854 | 13735 | -15.92 | <.0001 |
| sample\*pos | C | DroshaKO | 6 | 0 | . | . | . | . |
| sample\*pos | G | DroshaKO | 6 | 0 | . | . | . | . |
| sample\*pos | U | DroshaKO | 6 | 0 | . | . | . | . |
| sample\*pos | C | Wildtype | 1 | 0 | . | . | . | . |
| sample\*pos | G | Wildtype | 1 | 0 | . | . | . | . |
| sample\*pos | U | Wildtype | 1 | 0 | . | . | . | . |
| sample\*pos | C | Wildtype | 2 | 0 | . | . | . | . |
| sample\*pos | G | Wildtype | 2 | 0 | . | . | . | . |
| sample\*pos | U | Wildtype | 2 | 0 | . | . | . | . |
| sample\*pos | C | Wildtype | 3 | 0 | . | . | . | . |
| sample\*pos | G | Wildtype | 3 | 0 | . | . | . | . |
| sample\*pos | U | Wildtype | 3 | 0 | . | . | . | . |
| sample\*pos | C | Wildtype | 4 | 0 | . | . | . | . |
| sample\*pos | G | Wildtype | 4 | 0 | . | . | . | . |
| sample\*pos | U | Wildtype | 4 | 0 | . | . | . | . |
| sample\*pos | C | Wildtype | 5 | 0 | . | . | . | . |
| sample\*pos | G | Wildtype | 5 | 0 | . | . | . | . |
| sample\*pos | U | Wildtype | 5 | 0 | . | . | . | . |
| sample\*pos | C | Wildtype | 6 | 0 | . | . | . | . |
| sample\*pos | G | Wildtype | 6 | 0 | . | . | . | . |
| sample\*pos | U | Wildtype | 6 | 0 | . | . | . | . |

  
 

| Odds Ratio Estimates | | | | |
| --- | --- | --- | --- | --- |
| Comparison | Estimate | DF | 95% Confidence Limits | |
| C: sample DicerKO vs Wildtype | 6.598 | 2729 | 5.696 | 7.643 |
| G: sample DicerKO vs Wildtype | 2.188 | 2729 | 1.881 | 2.546 |
| U: sample DicerKO vs Wildtype | 2.040 | 2729 | 1.743 | 2.387 |
| C: sample DroshaKO vs Wildtype | 7.191 | 2729 | 6.238 | 8.289 |
| G: sample DroshaKO vs Wildtype | 4.113 | 2729 | 3.581 | 4.724 |
| U: sample DroshaKO vs Wildtype | 2.120 | 2729 | 1.806 | 2.490 |
| C: pos 1 vs 6 | 0.635 | 2729 | 0.524 | 0.771 |
| G: pos 1 vs 6 | 0.327 | 2729 | 0.267 | 0.402 |
| U: pos 1 vs 6 | 0.623 | 2729 | 0.512 | 0.759 |
| C: pos 2 vs 6 | 4.604 | 2729 | 3.687 | 5.748 |
| G: pos 2 vs 6 | 1.057 | 2729 | 0.833 | 1.341 |
| U: pos 2 vs 6 | 0.703 | 2729 | 0.537 | 0.921 |
| C: pos 3 vs 6 | 5.366 | 2729 | 4.362 | 6.600 |
| G: pos 3 vs 6 | 0.909 | 2729 | 0.729 | 1.134 |
| U: pos 3 vs 6 | 0.851 | 2729 | 0.660 | 1.098 |
| C: pos 4 vs 6 | 1.880 | 2729 | 1.526 | 2.315 |
| G: pos 4 vs 6 | 1.193 | 2729 | 0.972 | 1.464 |
| U: pos 4 vs 6 | 2.270 | 2729 | 1.835 | 2.809 |
| C: pos 5 vs 6 | 0.937 | 2729 | 0.772 | 1.138 |
| G: pos 5 vs 6 | 1.193 | 2729 | 0.994 | 1.431 |
| U: pos 5 vs 6 | 0.971 | 2729 | 0.785 | 1.201 |

  
 

| Type III Tests of Fixed Effects | | | | |
| --- | --- | --- | --- | --- |
| Effect | Num DF | Den DF | F Value | Pr > F |
| sample | 6 | 2729 | 189.90 | <.0001 |
| pos | 15 | 2729 | 138.39 | <.0001 |
| sample\*pos | 30 | 13735 | 56.61 | <.0001 |

```
> 
+ proc plm restore=gmxres noclprint plots=none;
+ lsmeans sample/ilink oddsratio adj=tukey cl e;
+ slice sample*pos/sliceby=pos diff oddsratio adj=tukey cl;
+ ods output slicediffs= sampleposdiffs diffs= samplediffs;
+ run;
```

```
> proc sort data=sampleposdiffs;
+ by sample slice base;
+ 
+ proc print data=sampleposdiffs;
+ where _sample = "Wildtype";
+ var sample _sample slice base OddsRatio AdjLowerOR AdjUpperOR Adjp;
+ run;
```

| Obs | sample | \_sample | Slice | base | OddsRatio | AdjLowerOR | AdjUpperOR | Adjp |
| --- | --- | --- | --- | --- | --- | --- | --- | --- |
| 2 | DicerKO | Wildtype | pos 1 | C | 19.295 | 13.080 | 28.463 | <.0001 |
| 4 | DicerKO | Wildtype | pos 1 | G | 0.232 | 0.143 | 0.375 | <.0001 |
| 6 | DicerKO | Wildtype | pos 1 | U | 4.634 | 3.267 | 6.574 | <.0001 |
| 8 | DicerKO | Wildtype | pos 2 | C | 28.375 | 16.308 | 49.370 | <.0001 |
| 10 | DicerKO | Wildtype | pos 2 | G | 4.498 | 2.554 | 7.921 | <.0001 |
| 12 | DicerKO | Wildtype | pos 2 | U | 5.732 | 2.942 | 11.169 | <.0001 |
| 14 | DicerKO | Wildtype | pos 3 | C | 8.552 | 5.355 | 13.655 | <.0001 |
| 16 | DicerKO | Wildtype | pos 3 | G | 3.576 | 2.081 | 6.143 | <.0001 |
| 18 | DicerKO | Wildtype | pos 3 | U | 1.555 | 0.866 | 2.791 | 0.1800 |
| 20 | DicerKO | Wildtype | pos 4 | C | 13.763 | 9.523 | 19.889 | <.0001 |
| 22 | DicerKO | Wildtype | pos 4 | G | 3.515 | 2.441 | 5.060 | <.0001 |
| 24 | DicerKO | Wildtype | pos 4 | U | 2.985 | 2.083 | 4.278 | <.0001 |
| 26 | DicerKO | Wildtype | pos 5 | C | 0.218 | 0.151 | 0.317 | <.0001 |
| 28 | DicerKO | Wildtype | pos 5 | G | 1.869 | 1.338 | 2.612 | <.0001 |
| 30 | DicerKO | Wildtype | pos 5 | U | 0.358 | 0.259 | 0.495 | <.0001 |
| 32 | DicerKO | Wildtype | pos 6 | C | 5.861 | 4.280 | 8.026 | <.0001 |
| 34 | DicerKO | Wildtype | pos 6 | G | 4.478 | 3.310 | 6.059 | <.0001 |
| 36 | DicerKO | Wildtype | pos 6 | U | 1.631 | 1.144 | 2.326 | 0.0036 |
| 37 | DroshaKO | Wildtype | pos 1 | C | 23.628 | 15.512 | 35.989 | <.0001 |
| 38 | DroshaKO | Wildtype | pos 1 | G | 3.858 | 2.842 | 5.236 | <.0001 |
| 39 | DroshaKO | Wildtype | pos 1 | U | 7.439 | 5.076 | 10.904 | <.0001 |
| 40 | DroshaKO | Wildtype | pos 2 | C | 6.091 | 4.381 | 8.468 | <.0001 |
| 41 | DroshaKO | Wildtype | pos 2 | G | 0.280 | 0.179 | 0.437 | <.0001 |
| 42 | DroshaKO | Wildtype | pos 2 | U | 0.904 | 0.532 | 1.537 | 0.8967 |
| 43 | DroshaKO | Wildtype | pos 3 | C | 2.249 | 1.614 | 3.134 | <.0001 |
| 44 | DroshaKO | Wildtype | pos 3 | G | 2.111 | 1.420 | 3.137 | <.0001 |
| 45 | DroshaKO | Wildtype | pos 3 | U | 0.423 | 0.255 | 0.702 | 0.0002 |
| 46 | DroshaKO | Wildtype | pos 4 | C | 20.537 | 12.911 | 32.668 | <.0001 |
| 47 | DroshaKO | Wildtype | pos 4 | G | 10.726 | 6.868 | 16.753 | <.0001 |
| 48 | DroshaKO | Wildtype | pos 4 | U | 9.446 | 6.076 | 14.686 | <.0001 |
| 49 | DroshaKO | Wildtype | pos 5 | C | 1.079 | 0.745 | 1.563 | 0.8798 |
| 50 | DroshaKO | Wildtype | pos 5 | G | 5.539 | 3.815 | 8.042 | <.0001 |
| 51 | DroshaKO | Wildtype | pos 5 | U | 0.189 | 0.117 | 0.307 | <.0001 |
| 52 | DroshaKO | Wildtype | pos 6 | C | 19.267 | 11.969 | 31.014 | <.0001 |
| 53 | DroshaKO | Wildtype | pos 6 | G | 35.734 | 22.791 | 56.027 | <.0001 |
| 54 | DroshaKO | Wildtype | pos 6 | U | 17.833 | 11.205 | 28.382 | <.0001 |

```
> * update the path in the OUTFILE statement below to match datapath;
+ * note that sas uses single backslash for paths, e.g. "C:\sasdata";
+ 
+ proc export data=work.sampleposdiffs
+   outfile="G:\PeterM_XXX\Analysis\Data\MethodsPaper\F_OREstimates_Fig2_081221.xlsx"
+   dbms=excel replace;
+   sheet="OR Position";
+ run;
```

# 4 Dicer vs. Wildtype

# 5 Drosha vs. Wildtype
