# Supplementary material for: SPOROS: A pipeline to analyze DISE/6mer seed toxicity
Source: PLoS Comput Biol. 2022 Mar 31;18(3):e1010022. doi: 10.1371/journal.pcbi.1010022 (PMC9004739; doi:10.1371/journal.pcbi.1010022)
Supplement: S4 Dataset — (ZIP) [file pcbi.1010022.s008.zip › Suppl. Dataset 4.pdf]

SPOROS pipeline analysis of seed composition data using multinomial mixed models

1 Read in Output F from GitHub

```
> ## read in raw data from GitHub
>
> f.down <- "https://raw.githubusercontent.com/ebartom/SPOROS/main/Figure3.SPOR
OSpaper/differential/ADnTPDvsCtrl/pvalue/sRNA/F_seedExpand.Delta.sRNA.dn.txt"
> f.up <- "https://raw.githubusercontent.com/ebartom/SPOROS/main/Figure3.SPOROS
paper/differential/ADnTPDvsCtrl/pvalue/sRNA/F_seedExpand.Delta.sRNA.up.txt"
>
> f.up <- "G:\\PeterM_XXX\\Analysis\\Data\\MethodsPaper\\Files for Figure 3\\F_
seedExpand.Delta.sRNA.up.txt"
> f.down <- "G:\\PeterM_XXX\\Analysis\\Data\\MethodsPaper\\Files for Figure 3\\
F_seedExpand.Delta.sRNA.dn.txt"
>
>
> down <- read.table(f.down, header=TRUE, sep="\t")
> up <- read.table(f.up, header=TRUE, sep="\t")
```

There are 998 seeds in Up data.

There are 1000 seeds in Down data.

There are 1998 seeds in the combined data set.

Example of each data set:

```
> # look at each file to check that data were read and combined correctly
> up %>% head
```

```
    Seed Sample    SeedID Pos Base
1 AAGGUU     Up Up.Delta.1   1    A
2 AAGGUU     Up Up.Delta.1   2    A
3 AAGGUU     Up Up.Delta.1   3    G
4 AAGGUU     Up Up.Delta.1   4    G
5 AAGGUU     Up Up.Delta.1   5    U
6 AAGGUU     Up Up.Delta.1   6    U
```

```
> down %>% head
```

```
    Seed Sample      SeedID Pos Base
1 AAACAG   Down Down.Delta.1   1    A
2 AAACAG   Down Down.Delta.1   2    A
3 AAACAG   Down Down.Delta.1   3    A
4 AAACAG   Down Down.Delta.1   4    C
5 AAACAG   Down Down.Delta.1   5    A
6 AAACAG   Down Down.Delta.1   6    G
```

```
> dataCombined %>% head
```

```
    Seed Sample    SeedID Pos Base
1 AAGGUU     Up Up.Delta.1   1    A
2 AAGGUU     Up Up.Delta.1   2    A
3 AAGGUU     Up Up.Delta.1   3    G
4 AAGGUU     Up Up.Delta.1   4    G
5 AAGGUU     Up Up.Delta.1   5    U
6 AAGGUU     Up Up.Delta.1   6    U
```

```
> dataCombined %>% tail
```

```
        Seed Sample         SeedID Pos Base
11983 UUUUCC   Down Down.Delta.1000   1    U
11984 UUUUCC   Down Down.Delta.1000   2    U
11985 UUUUCC   Down Down.Delta.1000   3    U
11986 UUUUCC   Down Down.Delta.1000   4    U
11987 UUUUCC   Down Down.Delta.1000   5    C
11988 UUUUCC   Down Down.Delta.1000   6    C
```

Do the counts match?

```
> # check total seeds/id's
> (nrow(up)/6 + nrow(down)/6 ==
+    length(unique(dataCombined$SeedID)))
```

```
   [1] TRUE
```

2 Save the data

Data saved for SAS analyses into:

```
   [1] "G:\\PeterM_XXX\\Analysis\\Data\\MethodsPaper\\data.sas.Fig3.2021-08-12.
csv"
```

3 SAS Analysis using PROC GLIMMIX

```
> * update the path in the INFILE statement to match datapath above;
+ * note that sas uses single backslash in paths, e.g. "C:\sasdata";
+
+ data seedlong;
+ informat seed $6. sample $20. seedid $30.;
+ infile "G:\\PeterM_XXX\\Analysis\\Data\\MethodsPaper\\data.sas.Fig3.2021-08-1
2.csv"
+   dlm="," firstobs=2;
+ input Seed $ sample $ seedid $ pos base $ ;
+ run;
+
+
+ * note: if the model has convergence issues, different estimation can be used
;
+ *       "method=laplace" was used in Fig. 3 data analysis;
+ *       "method = rmpl" was used in Fig. 2 data analysis;
+ proc glimmix data=seedlong outdesign = xx method=laplace;
+ class seed seedid sample(ref="Down") pos base(ref="A");
+ model base = sample pos sample*pos /dist=multinomial link=glogit s or(label)
ddfm=bw;
+ random intercept/ subject = seedid group=base type=chol;
+ store gmxres;
+ run;
```

Model Information	
**Data Set**	WORK.SEEDLONG
**Response Variable**	base
**Response Distribution**	Multinomial (nominal)
**Link Function**	Generalized Logit
**Variance Function**	Default
**Variance Matrix Blocked By**	seedid
**Estimation Technique**	Maximum Likelihood
**Likelihood Approximation**	Laplace
**Degrees of Freedom Method**	Between-Within

Class Level Information		
**Class**	**Levels**	**Values**
**seed**	88	AAACAG AAACCG AAGCGG AAGGUU AAUUGU AAUUUU ACGUGU ACUUCG AGACUA AGAGUG AGAUUA AGAUUC AGCUUC AGGAUC AGGCUC AGGGUC AGGUCC AGGUUC AGGUUG AGUACA AGUAGU AGUUUC AUCUUG AUGGAG AUUAGC AUUGGC AUUGUC CAAUAU CAGAUU CAGUAA CAUAAG CCAAGA CCAGAA CCCGAC CCCGGG CCGGUA CGGAGC CGGCCU CGGUAA CUAAAC CUCCAG CUCGGU CUGGAC GAACUG GAAGUG GAAUCG GAAUUG GAGGUU GAGUGU GAUUAU GAUUCC GAUUCU GAUUGU GGAAUG GGAGCG GGCAGU GGCCCC GGCGGA GGCUGU GGGAGC GGGAUU GGGUGU GGUAAA GGUAUU GGUUCC GGUUGU GUCAGU GUCGAU GUGUUA UAACUA UAUUGC UCACGG UCCAGA UCCCCG UCGACU UCGUGG UCUCUG UCUCUU UGACUG UGACUU UGAUUC UGCAUU UGGGGC UGUAUG UGUCUG UUGUGG UUUCUG UUUUCC
**seedid**	1998	Down.Delta.1 Down.Delta.10 Down.Delta.100 Down.Delta.1000 Down.Delta.101 Down.Delta.102 Down.Delta.103 Down.Delta.104 Down.Delta.105 Down.Delta.106 Down.Delta.107 Down.Delta.108 Down.Delta.109 Down.Delta.11 Down.Delta.110 Down.Delta.111 Down.Delta.112 Down.Delta.113 Down.Delta.114 Down.Delta.115 Down.Delta.116 Down.Delta.117 Down.Delta.118 Down.Delta.119 Down.Delta.12 Down.Delta.120 Down.Delta.121 Down.Delta.122 Down.Delta.123 Down.Delta.124

Down.Delta.125 Down.Delta.126 Down.Delta.127 Down.Delta.128 Down.Delta.129
Down.Delta.13 Down.Delta.130 Down.Delta.131 Down.Delta.132 Down.Delta.133
Down.Delta.134 Down.Delta.135 Down.Delta.136 Down.Delta.137 Down.Delta.138
Down.Delta.139 Down.Delta.14 Down.Delta.140 Down.Delta.141 Down.Delta.142
Down.Delta.143 Down.Delta.144 Down.Delta.145 Down.Delta.146 Down.Delta.147
Down.Delta.148 Down.Delta.149 Down.Delta.15 Down.Delta.150 Down.Delta.151
Down.Delta.152 Down.Delta.153 Down.Delta.154 Down.Delta.155 Down.Delta.156
Down.Delta.157 Down.Delta.158 Down.Delta.159 Down.Delta.16 Down.Delta.160
Down.Delta.161 Down.Delta.162 Down.Delta.163 Down.Delta.164 Down.Delta.165
Down.Delta.166 Down.Delta.167 Down.Delta.168 Down.Delta.169 Down.Delta.17
Down.Delta.170 Down.Delta.171 Down.Delta.172 Down.Delta.173 Down.Delta.174
Down.Delta.175 Down.Delta.176 Down.Delta.177 Down.Delta.178 Down.Delta.179
Down.Delta.18 Down.Delta.180 Down.Delta.181 Down.Delta.182 Down.Delta.183
Down.Delta.184 Down.Delta.185 Down.Delta.186 Down.Delta.187 Down.Delta.188
Down.Delta.189 Down.Delta.19 Down.Delta.190 Down.Delta.191 Down.Delta.192
Down.Delta.193 Down.Delta.194 Down.Delta.195 Down.Delta.196 Down.Delta.197
Down.Delta.198 Down.Delta.199 Down.Delta.2 Down.Delta.20 Down.Delta.200
Down.Delta.201 Down.Delta.202 Down.Delta.203 Down.Delta.204 Down.Delta.205
Down.Delta.206 Down.Delta.207 Down.Delta.208 Down.Delta.209 Down.Delta.21
Down.Delta.210 Down.Delta.211 Down.Delta.212 Down.Delta.213 Down.Delta.214
Down.Delta.215 Down.Delta.216 Down.Delta.217 Down.Delta.218 Down.Delta.219
Down.Delta.22 Down.Delta.220 Down.Delta.221 Down.Delta.222 Down.Delta.223
Down.Delta.224 Down.Delta.225 Down.Delta.226 Down.Delta.227 Down.Delta.228
Down.Delta.229 Down.Delta.23 Down.Delta.230 Down.Delta.231 Down.Delta.232
Down.Delta.233 Down.Delta.234 Down.Delta.235 Down.Delta.236 Down.Delta.237
Down.Delta.238 Down.Delta.239 Down.Delta.24 Down.Delta.240 Down.Delta.241
Down.Delta.242 Down.Delta.243 Down.Delta.244 Down.Delta.245 Down.Delta.246
Down.Delta.247 Down.Delta.248 Down.Delta.249 Down.Delta.25 Down.Delta.250
Down.Delta.251 Down.Delta.252 Down.Delta.253 Down.Delta.254 Down.Delta.255
Down.Delta.256 Down.Delta.257 Down.Delta.258 Down.Delta.259 Down.Delta.26
Down.Delta.260 Down.Delta.261 Down.Delta.262 Down.Delta.263 Down.Delta.264
Down.Delta.265 Down.Delta.266 Down.Delta.267 Down.Delta.268 Down.Delta.269
Down.Delta.27 Down.Delta.270 Down.Delta.271 Down.Delta.272 Down.Delta.273
Down.Delta.274 Down.Delta.275 Down.Delta.276 Down.Delta.277 Down.Delta.278
Down.Delta.279 Down.Delta.28 Down.Delta.280 Down.Delta.281 Down.Delta.282
Down.Delta.283 Down.Delta.284 Down.Delta.285 Down.Delta.286 Down.Delta.287
Down.Delta.288 Down.Delta.289 Down.Delta.29 Down.Delta.290 Down.Delta.291
Down.Delta.292 Down.Delta.293 Down.Delta.294 Down.Delta.295 Down.Delta.296
Down.Delta.297 Down.Delta.298 Down.Delta.299 Down.Delta.3 Down.Delta.30
Down.Delta.300 Down.Delta.301 Down.Delta.302 Down.Delta.303 Down.Delta.304
Down.Delta.305 Down.Delta.306 Down.Delta.307 Down.Delta.308 Down.Delta.309
Down.Delta.31 Down.Delta.310 Down.Delta.311 Down.Delta.312 Down.Delta.313

Down.Delta.314 Down.Delta.315 Down.Delta.316 Down.Delta.317 Down.Delta.318
Down.Delta.319 Down.Delta.32 Down.Delta.320 Down.Delta.321 Down.Delta.322
Down.Delta.323 Down.Delta.324 Down.Delta.325 Down.Delta.326 Down.Delta.327
Down.Delta.328 Down.Delta.329 Down.Delta.33 Down.Delta.330 Down.Delta.331
Down.Delta.332 Down.Delta.333 Down.Delta.334 Down.Delta.335 Down.Delta.336
Down.Delta.337 Down.Delta.338 Down.Delta.339 Down.Delta.34 Down.Delta.340
Down.Delta.341 Down.Delta.342 Down.Delta.343 Down.Delta.344 Down.Delta.345
Down.Delta.346 Down.Delta.347 Down.Delta.348 Down.Delta.349 Down.Delta.35
Down.Delta.350 Down.Delta.351 Down.Delta.352 Down.Delta.353 Down.Delta.354
Down.Delta.355 Down.Delta.356 Down.Delta.357 Down.Delta.358 Down.Delta.359
Down.Delta.36 Down.Delta.360 Down.Delta.361 Down.Delta.362 Down.Delta.363
Down.Delta.364 Down.Delta.365 Down.Delta.366 Down.Delta.367 Down.Delta.368
Down.Delta.369 Down.Delta.37 Down.Delta.370 Down.Delta.371 Down.Delta.372
Down.Delta.373 Down.Delta.374 Down.Delta.375 Down.Delta.376 Down.Delta.377
Down.Delta.378 Down.Delta.379 Down.Delta.38 Down.Delta.380 Down.Delta.381
Down.Delta.382 Down.Delta.383 Down.Delta.384 Down.Delta.385 Down.Delta.386
Down.Delta.387 Down.Delta.388 Down.Delta.389 Down.Delta.39 Down.Delta.390
Down.Delta.391 Down.Delta.392 Down.Delta.393 Down.Delta.394 Down.Delta.395
Down.Delta.396 Down.Delta.397 Down.Delta.398 Down.Delta.399 Down.Delta.4
Down.Delta.40 Down.Delta.400 Down.Delta.401 Down.Delta.402 Down.Delta.403
Down.Delta.404 Down.Delta.405 Down.Delta.406 Down.Delta.407 Down.Delta.408
Down.Delta.409 Down.Delta.41 Down.Delta.410 Down.Delta.411 Down.Delta.412
Down.Delta.413 Down.Delta.414 Down.Delta.415 Down.Delta.416 Down.Delta.417
Down.Delta.418 Down.Delta.419 Down.Delta.42 Down.Delta.420 Down.Delta.421
Down.Delta.422 Down.Delta.423 Down.Delta.424 Down.Delta.425 Down.Delta.426
Down.Delta.427 Down.Delta.428 Down.Delta.429 Down.Delta.43 Down.Delta.430
Down.Delta.431 Down.Delta.432 Down.Delta.433 Down.Delta.434 Down.Delta.435
Down.Delta.436 Down.Delta.437 Down.Delta.438 Down.Delta.439 Down.Delta.44
Down.Delta.440 Down.Delta.441 Down.Delta.442 Down.Delta.443 Down.Delta.444
Down.Delta.445 Down.Delta.446 Down.Delta.447 Down.Delta.448 Down.Delta.449
Down.Delta.45 Down.Delta.450 Down.Delta.451 Down.Delta.452 Down.Delta.453
Down.Delta.454 Down.Delta.455 Down.Delta.456 Down.Delta.457 Down.Delta.458
Down.Delta.459 Down.Delta.46 Down.Delta.460 Down.Delta.461 Down.Delta.462
Down.Delta.463 Down.Delta.464 Down.Delta.465 Down.Delta.466 Down.Delta.467
Down.Delta.468 Down.Delta.469 Down.Delta.47 Down.Delta.470 Down.Delta.471
Down.Delta.472 Down.Delta.473 Down.Delta.474 Down.Delta.475 Down.Delta.476
Down.Delta.477 Down.Delta.478 Down.Delta.479 Down.Delta.48 Down.Delta.480
Down.Delta.481 Down.Delta.482 Down.Delta.483 Down.Delta.484 Down.Delta.485
Down.Delta.486 Down.Delta.487 Down.Delta.488 Down.Delta.489 Down.Delta.49
Down.Delta.490 Down.Delta.491 Down.Delta.492 Down.Delta.493 Down.Delta.494
Down.Delta.495 Down.Delta.496 Down.Delta.497 Down.Delta.498 Down.Delta.499
Down.Delta.5 Down.Delta.50 Down.Delta.500 Down.Delta.501 Down.Delta.502

Down.Delta.503 Down.Delta.504 Down.Delta.505 Down.Delta.506 Down.Delta.507
Down.Delta.508 Down.Delta.509 Down.Delta.51 Down.Delta.510 Down.Delta.511
Down.Delta.512 Down.Delta.513 Down.Delta.514 Down.Delta.515 Down.Delta.516
Down.Delta.517 Down.Delta.518 Down.Delta.519 Down.Delta.52 Down.Delta.520
Down.Delta.521 Down.Delta.522 Down.Delta.523 Down.Delta.524 Down.Delta.525
Down.Delta.526 Down.Delta.527 Down.Delta.528 Down.Delta.529 Down.Delta.53
Down.Delta.530 Down.Delta.531 Down.Delta.532 Down.Delta.533 Down.Delta.534
Down.Delta.535 Down.Delta.536 Down.Delta.537 Down.Delta.538 Down.Delta.539
Down.Delta.54 Down.Delta.540 Down.Delta.541 Down.Delta.542 Down.Delta.543
Down.Delta.544 Down.Delta.545 Down.Delta.546 Down.Delta.547 Down.Delta.548
Down.Delta.549 Down.Delta.55 Down.Delta.550 Down.Delta.551 Down.Delta.552
Down.Delta.553 Down.Delta.554 Down.Delta.555 Down.Delta.556 Down.Delta.557
Down.Delta.558 Down.Delta.559 Down.Delta.56 Down.Delta.560 Down.Delta.561
Down.Delta.562 Down.Delta.563 Down.Delta.564 Down.Delta.565 Down.Delta.566
Down.Delta.567 Down.Delta.568 Down.Delta.569 Down.Delta.57 Down.Delta.570
Down.Delta.571 Down.Delta.572 Down.Delta.573 Down.Delta.574 Down.Delta.575
Down.Delta.576 Down.Delta.577 Down.Delta.578 Down.Delta.579 Down.Delta.58
Down.Delta.580 Down.Delta.581 Down.Delta.582 Down.Delta.583 Down.Delta.584
Down.Delta.585 Down.Delta.586 Down.Delta.587 Down.Delta.588 Down.Delta.589
Down.Delta.59 Down.Delta.590 Down.Delta.591 Down.Delta.592 Down.Delta.593
Down.Delta.594 Down.Delta.595 Down.Delta.596 Down.Delta.597 Down.Delta.598
Down.Delta.599 Down.Delta.6 Down.Delta.60 Down.Delta.600 Down.Delta.601
Down.Delta.602 Down.Delta.603 Down.Delta.604 Down.Delta.605 Down.Delta.606
Down.Delta.607 Down.Delta.608 Down.Delta.609 Down.Delta.61 Down.Delta.610
Down.Delta.611 Down.Delta.612 Down.Delta.613 Down.Delta.614 Down.Delta.615
Down.Delta.616 Down.Delta.617 Down.Delta.618 Down.Delta.619 Down.Delta.62
Down.Delta.620 Down.Delta.621 Down.Delta.622 Down.Delta.623 Down.Delta.624
Down.Delta.625 Down.Delta.626 Down.Delta.627 Down.Delta.628 Down.Delta.629
Down.Delta.63 Down.Delta.630 Down.Delta.631 Down.Delta.632 Down.Delta.633
Down.Delta.634 Down.Delta.635 Down.Delta.636 Down.Delta.637 Down.Delta.638
Down.Delta.639 Down.Delta.64 Down.Delta.640 Down.Delta.641 Down.Delta.642
Down.Delta.643 Down.Delta.644 Down.Delta.645 Down.Delta.646 Down.Delta.647
Down.Delta.648 Down.Delta.649 Down.Delta.65 Down.Delta.650 Down.Delta.651
Down.Delta.652 Down.Delta.653 Down.Delta.654 Down.Delta.655 Down.Delta.656
Down.Delta.657 Down.Delta.658 Down.Delta.659 Down.Delta.66 Down.Delta.660
Down.Delta.661 Down.Delta.662 Down.Delta.663 Down.Delta.664 Down.Delta.665
Down.Delta.666 Down.Delta.667 Down.Delta.668 Down.Delta.669 Down.Delta.67
Down.Delta.670 Down.Delta.671 Down.Delta.672 Down.Delta.673 Down.Delta.674
Down.Delta.675 Down.Delta.676 Down.Delta.677 Down.Delta.678 Down.Delta.679
Down.Delta.68 Down.Delta.680 Down.Delta.681 Down.Delta.682 Down.Delta.683
Down.Delta.684 Down.Delta.685 Down.Delta.686 Down.Delta.687 Down.Delta.688
Down.Delta.689 Down.Delta.69 Down.Delta.690 Down.Delta.691 Down.Delta.692

Down.Delta.693 Down.Delta.694 Down.Delta.695 Down.Delta.696 Down.Delta.697
Down.Delta.698 Down.Delta.699 Down.Delta.7 Down.Delta.70 Down.Delta.700
Down.Delta.701 Down.Delta.702 Down.Delta.703 Down.Delta.704 Down.Delta.705
Down.Delta.706 Down.Delta.707 Down.Delta.708 Down.Delta.709 Down.Delta.71
Down.Delta.710 Down.Delta.711 Down.Delta.712 Down.Delta.713 Down.Delta.714
Down.Delta.715 Down.Delta.716 Down.Delta.717 Down.Delta.718 Down.Delta.719
Down.Delta.72 Down.Delta.720 Down.Delta.721 Down.Delta.722 Down.Delta.723
Down.Delta.724 Down.Delta.725 Down.Delta.726 Down.Delta.727 Down.Delta.728
Down.Delta.729 Down.Delta.73 Down.Delta.730 Down.Delta.731 Down.Delta.732
Down.Delta.733 Down.Delta.734 Down.Delta.735 Down.Delta.736 Down.Delta.737
Down.Delta.738 Down.Delta.739 Down.Delta.74 Down.Delta.740 Down.Delta.741
Down.Delta.742 Down.Delta.743 Down.Delta.744 Down.Delta.745 Down.Delta.746
Down.Delta.747 Down.Delta.748 Down.Delta.749 Down.Delta.75 Down.Delta.750
Down.Delta.751 Down.Delta.752 Down.Delta.753 Down.Delta.754 Down.Delta.755
Down.Delta.756 Down.Delta.757 Down.Delta.758 Down.Delta.759 Down.Delta.76
Down.Delta.760 Down.Delta.761 Down.Delta.762 Down.Delta.763 Down.Delta.764
Down.Delta.765 Down.Delta.766 Down.Delta.767 Down.Delta.768 Down.Delta.769
Down.Delta.77 Down.Delta.770 Down.Delta.771 Down.Delta.772 Down.Delta.773
Down.Delta.774 Down.Delta.775 Down.Delta.776 Down.Delta.777 Down.Delta.778
Down.Delta.779 Down.Delta.78 Down.Delta.780 Down.Delta.781 Down.Delta.782
Down.Delta.783 Down.Delta.784 Down.Delta.785 Down.Delta.786 Down.Delta.787
Down.Delta.788 Down.Delta.789 Down.Delta.79 Down.Delta.790 Down.Delta.791
Down.Delta.792 Down.Delta.793 Down.Delta.794 Down.Delta.795 Down.Delta.796
Down.Delta.797 Down.Delta.798 Down.Delta.799 Down.Delta.8 Down.Delta.80
Down.Delta.800 Down.Delta.801 Down.Delta.802 Down.Delta.803 Down.Delta.804
Down.Delta.805 Down.Delta.806 Down.Delta.807 Down.Delta.808 Down.Delta.809
Down.Delta.81 Down.Delta.810 Down.Delta.811 Down.Delta.812 Down.Delta.813
Down.Delta.814 Down.Delta.815 Down.Delta.816 Down.Delta.817 Down.Delta.818
Down.Delta.819 Down.Delta.82 Down.Delta.820 Down.Delta.821 Down.Delta.822
Down.Delta.823 Down.Delta.824 Down.Delta.825 Down.Delta.826 Down.Delta.827
Down.Delta.828 Down.Delta.829 Down.Delta.83 Down.Delta.830 Down.Delta.831
Down.Delta.832 Down.Delta.833 Down.Delta.834 Down.Delta.835 Down.Delta.836
Down.Delta.837 Down.Delta.838 Down.Delta.839 Down.Delta.84 Down.Delta.840
Down.Delta.841 Down.Delta.842 Down.Delta.843 Down.Delta.844 Down.Delta.845
Down.Delta.846 Down.Delta.847 Down.Delta.848 Down.Delta.849 Down.Delta.85
Down.Delta.850 Down.Delta.851 Down.Delta.852 Down.Delta.853 Down.Delta.854
Down.Delta.855 Down.Delta.856 Down.Delta.857 Down.Delta.858 Down.Delta.859
Down.Delta.86 Down.Delta.860 Down.Delta.861 Down.Delta.862 Down.Delta.863
Down.Delta.864 Down.Delta.865 Down.Delta.866 Down.Delta.867 Down.Delta.868
Down.Delta.869 Down.Delta.87 Down.Delta.870 Down.Delta.871 Down.Delta.872
Down.Delta.873 Down.Delta.874 Down.Delta.875 Down.Delta.876 Down.Delta.877
Down.Delta.878 Down.Delta.879 Down.Delta.88 Down.Delta.880 Down.Delta.881

Down.Delta.882 Down.Delta.883 Down.Delta.884 Down.Delta.885 Down.Delta.886
Down.Delta.887 Down.Delta.888 Down.Delta.889 Down.Delta.89 Down.Delta.890
Down.Delta.891 Down.Delta.892 Down.Delta.893 Down.Delta.894 Down.Delta.895
Down.Delta.896 Down.Delta.897 Down.Delta.898 Down.Delta.899 Down.Delta.9
Down.Delta.90 Down.Delta.900 Down.Delta.901 Down.Delta.902 Down.Delta.903
Down.Delta.904 Down.Delta.905 Down.Delta.906 Down.Delta.907 Down.Delta.908
Down.Delta.909 Down.Delta.91 Down.Delta.910 Down.Delta.911 Down.Delta.912
Down.Delta.913 Down.Delta.914 Down.Delta.915 Down.Delta.916 Down.Delta.917
Down.Delta.918 Down.Delta.919 Down.Delta.92 Down.Delta.920 Down.Delta.921
Down.Delta.922 Down.Delta.923 Down.Delta.924 Down.Delta.925 Down.Delta.926
Down.Delta.927 Down.Delta.928 Down.Delta.929 Down.Delta.93 Down.Delta.930
Down.Delta.931 Down.Delta.932 Down.Delta.933 Down.Delta.934 Down.Delta.935
Down.Delta.936 Down.Delta.937 Down.Delta.938 Down.Delta.939 Down.Delta.94
Down.Delta.940 Down.Delta.941 Down.Delta.942 Down.Delta.943 Down.Delta.944
Down.Delta.945 Down.Delta.946 Down.Delta.947 Down.Delta.948 Down.Delta.949
Down.Delta.95 Down.Delta.950 Down.Delta.951 Down.Delta.952 Down.Delta.953
Down.Delta.954 Down.Delta.955 Down.Delta.956 Down.Delta.957 Down.Delta.958
Down.Delta.959 Down.Delta.96 Down.Delta.960 Down.Delta.961 Down.Delta.962
Down.Delta.963 Down.Delta.964 Down.Delta.965 Down.Delta.966 Down.Delta.967
Down.Delta.968 Down.Delta.969 Down.Delta.97 Down.Delta.970 Down.Delta.971
Down.Delta.972 Down.Delta.973 Down.Delta.974 Down.Delta.975 Down.Delta.976
Down.Delta.977 Down.Delta.978 Down.Delta.979 Down.Delta.98 Down.Delta.980
Down.Delta.981 Down.Delta.982 Down.Delta.983 Down.Delta.984 Down.Delta.985
Down.Delta.986 Down.Delta.987 Down.Delta.988 Down.Delta.989 Down.Delta.99
Down.Delta.990 Down.Delta.991 Down.Delta.992 Down.Delta.993 Down.Delta.994
Down.Delta.995 Down.Delta.996 Down.Delta.997 Down.Delta.998 Down.Delta.999
Up.Delta.1 Up.Delta.10 Up.Delta.100 Up.Delta.101 Up.Delta.102 Up.Delta.103
Up.Delta.104 Up.Delta.105 Up.Delta.106 Up.Delta.107 Up.Delta.108 Up.Delta.109
Up.Delta.11 Up.Delta.110 Up.Delta.111 Up.Delta.112 Up.Delta.113 Up.Delta.114
Up.Delta.115 Up.Delta.116 Up.Delta.117 Up.Delta.118 Up.Delta.119 Up.Delta.12
Up.Delta.120 Up.Delta.121 Up.Delta.122 Up.Delta.123 Up.Delta.124 Up.Delta.125
Up.Delta.126 Up.Delta.127 Up.Delta.128 Up.Delta.129 Up.Delta.13 Up.Delta.130
Up.Delta.131 Up.Delta.132 Up.Delta.133 Up.Delta.134 Up.Delta.135 Up.Delta.136
Up.Delta.137 Up.Delta.138 Up.Delta.139 Up.Delta.14 Up.Delta.140 Up.Delta.141
Up.Delta.142 Up.Delta.143 Up.Delta.144 Up.Delta.145 Up.Delta.146 Up.Delta.147
Up.Delta.148 Up.Delta.149 Up.Delta.15 Up.Delta.150 Up.Delta.151 Up.Delta.152
Up.Delta.153 Up.Delta.154 Up.Delta.155 Up.Delta.156 Up.Delta.157 Up.Delta.158
Up.Delta.159 Up.Delta.16 Up.Delta.160 Up.Delta.161 Up.Delta.162 Up.Delta.163
Up.Delta.164 Up.Delta.165 Up.Delta.166 Up.Delta.167 Up.Delta.168 Up.Delta.169
Up.Delta.17 Up.Delta.170 Up.Delta.171 Up.Delta.172 Up.Delta.173 Up.Delta.174
Up.Delta.175 Up.Delta.176 Up.Delta.177 Up.Delta.178 Up.Delta.179 Up.Delta.18
Up.Delta.180 Up.Delta.181 Up.Delta.182 Up.Delta.183 Up.Delta.184 Up.Delta.185

Up.Delta.186 Up.Delta.187 Up.Delta.188 Up.Delta.189 Up.Delta.19 Up.Delta.190
Up.Delta.191 Up.Delta.192 Up.Delta.193 Up.Delta.194 Up.Delta.195 Up.Delta.196
Up.Delta.197 Up.Delta.198 Up.Delta.199 Up.Delta.2 Up.Delta.20 Up.Delta.200
Up.Delta.201 Up.Delta.202 Up.Delta.203 Up.Delta.204 Up.Delta.205 Up.Delta.206
Up.Delta.207 Up.Delta.208 Up.Delta.209 Up.Delta.21 Up.Delta.210 Up.Delta.211
Up.Delta.212 Up.Delta.213 Up.Delta.214 Up.Delta.215 Up.Delta.216 Up.Delta.217
Up.Delta.218 Up.Delta.219 Up.Delta.22 Up.Delta.220 Up.Delta.221 Up.Delta.222
Up.Delta.223 Up.Delta.224 Up.Delta.225 Up.Delta.226 Up.Delta.227 Up.Delta.228
Up.Delta.229 Up.Delta.23 Up.Delta.230 Up.Delta.231 Up.Delta.232 Up.Delta.233
Up.Delta.234 Up.Delta.235 Up.Delta.236 Up.Delta.237 Up.Delta.238 Up.Delta.239
Up.Delta.24 Up.Delta.240 Up.Delta.241 Up.Delta.242 Up.Delta.243 Up.Delta.244
Up.Delta.245 Up.Delta.246 Up.Delta.247 Up.Delta.248 Up.Delta.249 Up.Delta.25
Up.Delta.250 Up.Delta.251 Up.Delta.252 Up.Delta.253 Up.Delta.254 Up.Delta.255
Up.Delta.256 Up.Delta.257 Up.Delta.258 Up.Delta.259 Up.Delta.26 Up.Delta.260
Up.Delta.261 Up.Delta.262 Up.Delta.263 Up.Delta.264 Up.Delta.265 Up.Delta.266
Up.Delta.267 Up.Delta.268 Up.Delta.269 Up.Delta.27 Up.Delta.270 Up.Delta.271
Up.Delta.272 Up.Delta.273 Up.Delta.274 Up.Delta.275 Up.Delta.276 Up.Delta.277
Up.Delta.278 Up.Delta.279 Up.Delta.28 Up.Delta.280 Up.Delta.281 Up.Delta.282
Up.Delta.283 Up.Delta.284 Up.Delta.285 Up.Delta.286 Up.Delta.287 Up.Delta.288
Up.Delta.289 Up.Delta.29 Up.Delta.290 Up.Delta.291 Up.Delta.292 Up.Delta.293
Up.Delta.294 Up.Delta.295 Up.Delta.296 Up.Delta.297 Up.Delta.298 Up.Delta.299
Up.Delta.3 Up.Delta.30 Up.Delta.300 Up.Delta.301 Up.Delta.302 Up.Delta.303
Up.Delta.304 Up.Delta.305 Up.Delta.306 Up.Delta.307 Up.Delta.308 Up.Delta.309
Up.Delta.31 Up.Delta.310 Up.Delta.311 Up.Delta.312 Up.Delta.313 Up.Delta.314
Up.Delta.315 Up.Delta.316 Up.Delta.317 Up.Delta.318 Up.Delta.319 Up.Delta.32
Up.Delta.320 Up.Delta.321 Up.Delta.322 Up.Delta.323 Up.Delta.324 Up.Delta.325
Up.Delta.326 Up.Delta.327 Up.Delta.328 Up.Delta.329 Up.Delta.33 Up.Delta.330
Up.Delta.331 Up.Delta.332 Up.Delta.333 Up.Delta.334 Up.Delta.335 Up.Delta.336
Up.Delta.337 Up.Delta.338 Up.Delta.339 Up.Delta.34 Up.Delta.340 Up.Delta.341
Up.Delta.342 Up.Delta.343 Up.Delta.344 Up.Delta.345 Up.Delta.346 Up.Delta.347
Up.Delta.348 Up.Delta.349 Up.Delta.35 Up.Delta.350 Up.Delta.351 Up.Delta.352
Up.Delta.353 Up.Delta.354 Up.Delta.355 Up.Delta.356 Up.Delta.357 Up.Delta.358
Up.Delta.359 Up.Delta.36 Up.Delta.360 Up.Delta.361 Up.Delta.362 Up.Delta.363
Up.Delta.364 Up.Delta.365 Up.Delta.366 Up.Delta.367 Up.Delta.368 Up.Delta.369
Up.Delta.37 Up.Delta.370 Up.Delta.371 Up.Delta.372 Up.Delta.373 Up.Delta.374
Up.Delta.375 Up.Delta.376 Up.Delta.377 Up.Delta.378 Up.Delta.379 Up.Delta.38
Up.Delta.380 Up.Delta.381 Up.Delta.382 Up.Delta.383 Up.Delta.384 Up.Delta.385
Up.Delta.386 Up.Delta.387 Up.Delta.388 Up.Delta.389 Up.Delta.39 Up.Delta.390
Up.Delta.391 Up.Delta.392 Up.Delta.393 Up.Delta.394 Up.Delta.395 Up.Delta.396
Up.Delta.397 Up.Delta.398 Up.Delta.399 Up.Delta.4 Up.Delta.40 Up.Delta.400
Up.Delta.401 Up.Delta.402 Up.Delta.403 Up.Delta.404 Up.Delta.405 Up.Delta.406
Up.Delta.407 Up.Delta.408 Up.Delta.409 Up.Delta.41 Up.Delta.410 Up.Delta.411

Up.Delta.412 Up.Delta.413 Up.Delta.414 Up.Delta.415 Up.Delta.416 Up.Delta.417
Up.Delta.418 Up.Delta.419 Up.Delta.42 Up.Delta.420 Up.Delta.421 Up.Delta.422
Up.Delta.423 Up.Delta.424 Up.Delta.425 Up.Delta.426 Up.Delta.427 Up.Delta.428
Up.Delta.429 Up.Delta.43 Up.Delta.430 Up.Delta.431 Up.Delta.432 Up.Delta.433
Up.Delta.434 Up.Delta.435 Up.Delta.436 Up.Delta.437 Up.Delta.438 Up.Delta.439
Up.Delta.44 Up.Delta.440 Up.Delta.441 Up.Delta.442 Up.Delta.443 Up.Delta.444
Up.Delta.445 Up.Delta.446 Up.Delta.447 Up.Delta.448 Up.Delta.449 Up.Delta.45
Up.Delta.450 Up.Delta.451 Up.Delta.452 Up.Delta.453 Up.Delta.454 Up.Delta.455
Up.Delta.456 Up.Delta.457 Up.Delta.458 Up.Delta.459 Up.Delta.46 Up.Delta.460
Up.Delta.461 Up.Delta.462 Up.Delta.463 Up.Delta.464 Up.Delta.465 Up.Delta.466
Up.Delta.467 Up.Delta.468 Up.Delta.469 Up.Delta.47 Up.Delta.470 Up.Delta.471
Up.Delta.472 Up.Delta.473 Up.Delta.474 Up.Delta.475 Up.Delta.476 Up.Delta.477
Up.Delta.478 Up.Delta.479 Up.Delta.48 Up.Delta.480 Up.Delta.481 Up.Delta.482
Up.Delta.483 Up.Delta.484 Up.Delta.485 Up.Delta.486 Up.Delta.487 Up.Delta.488
Up.Delta.489 Up.Delta.49 Up.Delta.490 Up.Delta.491 Up.Delta.492 Up.Delta.493
Up.Delta.494 Up.Delta.495 Up.Delta.496 Up.Delta.497 Up.Delta.498 Up.Delta.499
Up.Delta.5 Up.Delta.50 Up.Delta.500 Up.Delta.501 Up.Delta.502 Up.Delta.503
Up.Delta.504 Up.Delta.505 Up.Delta.506 Up.Delta.507 Up.Delta.508 Up.Delta.509
Up.Delta.51 Up.Delta.510 Up.Delta.511 Up.Delta.512 Up.Delta.513 Up.Delta.514
Up.Delta.515 Up.Delta.516 Up.Delta.517 Up.Delta.518 Up.Delta.519 Up.Delta.52
Up.Delta.520 Up.Delta.521 Up.Delta.522 Up.Delta.523 Up.Delta.524 Up.Delta.525
Up.Delta.526 Up.Delta.527 Up.Delta.528 Up.Delta.529 Up.Delta.53 Up.Delta.530
Up.Delta.531 Up.Delta.532 Up.Delta.533 Up.Delta.534 Up.Delta.535 Up.Delta.536
Up.Delta.537 Up.Delta.538 Up.Delta.539 Up.Delta.54 Up.Delta.540 Up.Delta.541
Up.Delta.542 Up.Delta.543 Up.Delta.544 Up.Delta.545 Up.Delta.546 Up.Delta.547
Up.Delta.548 Up.Delta.549 Up.Delta.55 Up.Delta.550 Up.Delta.551 Up.Delta.552
Up.Delta.553 Up.Delta.554 Up.Delta.555 Up.Delta.556 Up.Delta.557 Up.Delta.558
Up.Delta.559 Up.Delta.56 Up.Delta.560 Up.Delta.561 Up.Delta.562 Up.Delta.563
Up.Delta.564 Up.Delta.565 Up.Delta.566 Up.Delta.567 Up.Delta.568 Up.Delta.569
Up.Delta.57 Up.Delta.570 Up.Delta.571 Up.Delta.572 Up.Delta.573 Up.Delta.574
Up.Delta.575 Up.Delta.576 Up.Delta.577 Up.Delta.578 Up.Delta.579 Up.Delta.58
Up.Delta.580 Up.Delta.581 Up.Delta.582 Up.Delta.583 Up.Delta.584 Up.Delta.585
Up.Delta.586 Up.Delta.587 Up.Delta.588 Up.Delta.589 Up.Delta.59 Up.Delta.590
Up.Delta.591 Up.Delta.592 Up.Delta.593 Up.Delta.594 Up.Delta.595 Up.Delta.596
Up.Delta.597 Up.Delta.598 Up.Delta.599 Up.Delta.6 Up.Delta.60 Up.Delta.600
Up.Delta.601 Up.Delta.602 Up.Delta.603 Up.Delta.604 Up.Delta.605 Up.Delta.606
Up.Delta.607 Up.Delta.608 Up.Delta.609 Up.Delta.61 Up.Delta.610 Up.Delta.611
Up.Delta.612 Up.Delta.613 Up.Delta.614 Up.Delta.615 Up.Delta.616 Up.Delta.617
Up.Delta.618 Up.Delta.619 Up.Delta.62 Up.Delta.620 Up.Delta.621 Up.Delta.622
Up.Delta.623 Up.Delta.624 Up.Delta.625 Up.Delta.626 Up.Delta.627 Up.Delta.628
Up.Delta.629 Up.Delta.63 Up.Delta.630 Up.Delta.631 Up.Delta.632 Up.Delta.633
Up.Delta.634 Up.Delta.635 Up.Delta.636 Up.Delta.637 Up.Delta.638 Up.Delta.639

Up.Delta.64 Up.Delta.640 Up.Delta.641 Up.Delta.642 Up.Delta.643 Up.Delta.644
Up.Delta.645 Up.Delta.646 Up.Delta.647 Up.Delta.648 Up.Delta.649 Up.Delta.65
Up.Delta.650 Up.Delta.651 Up.Delta.652 Up.Delta.653 Up.Delta.654 Up.Delta.655
Up.Delta.656 Up.Delta.657 Up.Delta.658 Up.Delta.659 Up.Delta.66 Up.Delta.660
Up.Delta.661 Up.Delta.662 Up.Delta.663 Up.Delta.664 Up.Delta.665 Up.Delta.666
Up.Delta.667 Up.Delta.668 Up.Delta.669 Up.Delta.67 Up.Delta.670 Up.Delta.671
Up.Delta.672 Up.Delta.673 Up.Delta.674 Up.Delta.675 Up.Delta.676 Up.Delta.677
Up.Delta.678 Up.Delta.679 Up.Delta.68 Up.Delta.680 Up.Delta.681 Up.Delta.682
Up.Delta.683 Up.Delta.684 Up.Delta.685 Up.Delta.686 Up.Delta.687 Up.Delta.688
Up.Delta.689 Up.Delta.69 Up.Delta.690 Up.Delta.691 Up.Delta.692 Up.Delta.693
Up.Delta.694 Up.Delta.695 Up.Delta.696 Up.Delta.697 Up.Delta.698 Up.Delta.699
Up.Delta.7 Up.Delta.70 Up.Delta.700 Up.Delta.701 Up.Delta.702 Up.Delta.703
Up.Delta.704 Up.Delta.705 Up.Delta.706 Up.Delta.707 Up.Delta.708 Up.Delta.709
Up.Delta.71 Up.Delta.710 Up.Delta.711 Up.Delta.712 Up.Delta.713 Up.Delta.714
Up.Delta.715 Up.Delta.716 Up.Delta.717 Up.Delta.718 Up.Delta.719 Up.Delta.72
Up.Delta.720 Up.Delta.721 Up.Delta.722 Up.Delta.723 Up.Delta.724 Up.Delta.725
Up.Delta.726 Up.Delta.727 Up.Delta.728 Up.Delta.729 Up.Delta.73 Up.Delta.730
Up.Delta.731 Up.Delta.732 Up.Delta.733 Up.Delta.734 Up.Delta.735 Up.Delta.736
Up.Delta.737 Up.Delta.738 Up.Delta.739 Up.Delta.74 Up.Delta.740 Up.Delta.741
Up.Delta.742 Up.Delta.743 Up.Delta.744 Up.Delta.745 Up.Delta.746 Up.Delta.747
Up.Delta.748 Up.Delta.749 Up.Delta.75 Up.Delta.750 Up.Delta.751 Up.Delta.752
Up.Delta.753 Up.Delta.754 Up.Delta.755 Up.Delta.756 Up.Delta.757 Up.Delta.758
Up.Delta.759 Up.Delta.76 Up.Delta.760 Up.Delta.761 Up.Delta.762 Up.Delta.763
Up.Delta.764 Up.Delta.765 Up.Delta.766 Up.Delta.767 Up.Delta.768 Up.Delta.769
Up.Delta.77 Up.Delta.770 Up.Delta.771 Up.Delta.772 Up.Delta.773 Up.Delta.774
Up.Delta.775 Up.Delta.776 Up.Delta.777 Up.Delta.778 Up.Delta.779 Up.Delta.78
Up.Delta.780 Up.Delta.781 Up.Delta.782 Up.Delta.783 Up.Delta.784 Up.Delta.785
Up.Delta.786 Up.Delta.787 Up.Delta.788 Up.Delta.789 Up.Delta.79 Up.Delta.790
Up.Delta.791 Up.Delta.792 Up.Delta.793 Up.Delta.794 Up.Delta.795 Up.Delta.796
Up.Delta.797 Up.Delta.798 Up.Delta.799 Up.Delta.8 Up.Delta.80 Up.Delta.800
Up.Delta.801 Up.Delta.802 Up.Delta.803 Up.Delta.804 Up.Delta.805 Up.Delta.806
Up.Delta.807 Up.Delta.808 Up.Delta.809 Up.Delta.81 Up.Delta.810 Up.Delta.811
Up.Delta.812 Up.Delta.813 Up.Delta.814 Up.Delta.815 Up.Delta.816 Up.Delta.817
Up.Delta.818 Up.Delta.819 Up.Delta.82 Up.Delta.820 Up.Delta.821 Up.Delta.822
Up.Delta.823 Up.Delta.824 Up.Delta.825 Up.Delta.826 Up.Delta.827 Up.Delta.828
Up.Delta.829 Up.Delta.83 Up.Delta.830 Up.Delta.831 Up.Delta.832 Up.Delta.833
Up.Delta.834 Up.Delta.835 Up.Delta.836 Up.Delta.837 Up.Delta.838 Up.Delta.839
Up.Delta.84 Up.Delta.840 Up.Delta.841 Up.Delta.842 Up.Delta.843 Up.Delta.844
Up.Delta.845 Up.Delta.846 Up.Delta.847 Up.Delta.848 Up.Delta.849 Up.Delta.85
Up.Delta.850 Up.Delta.851 Up.Delta.852 Up.Delta.853 Up.Delta.854 Up.Delta.855
Up.Delta.856 Up.Delta.857 Up.Delta.858 Up.Delta.859 Up.Delta.86 Up.Delta.860
Up.Delta.861 Up.Delta.862 Up.Delta.863 Up.Delta.864 Up.Delta.865 Up.Delta.866

		Up.Delta.867 Up.Delta.868 Up.Delta.869 Up.Delta.87 Up.Delta.870 Up.Delta.871 Up.Delta.872 Up.Delta.873 Up.Delta.874 Up.Delta.875 Up.Delta.876 Up.Delta.877 Up.Delta.878 Up.Delta.879 Up.Delta.88 Up.Delta.880 Up.Delta.881 Up.Delta.882 Up.Delta.883 Up.Delta.884 Up.Delta.885 Up.Delta.886 Up.Delta.887 Up.Delta.888 Up.Delta.889 Up.Delta.89 Up.Delta.890 Up.Delta.891 Up.Delta.892 Up.Delta.893 Up.Delta.894 Up.Delta.895 Up.Delta.896 Up.Delta.897 Up.Delta.898 Up.Delta.899 Up.Delta.9 Up.Delta.90 Up.Delta.900 Up.Delta.901 Up.Delta.902 Up.Delta.903 Up.Delta.904 Up.Delta.905 Up.Delta.906 Up.Delta.907 Up.Delta.908 Up.Delta.909 Up.Delta.91 Up.Delta.910 Up.Delta.911 Up.Delta.912 Up.Delta.913 Up.Delta.914 Up.Delta.915 Up.Delta.916 Up.Delta.917 Up.Delta.918 Up.Delta.919 Up.Delta.92 Up.Delta.920 Up.Delta.921 Up.Delta.922 Up.Delta.923 Up.Delta.924 Up.Delta.925 Up.Delta.926 Up.Delta.927 Up.Delta.928 Up.Delta.929 Up.Delta.93 Up.Delta.930 Up.Delta.931 Up.Delta.932 Up.Delta.933 Up.Delta.934 Up.Delta.935 Up.Delta.936 Up.Delta.937 Up.Delta.938 Up.Delta.939 Up.Delta.94 Up.Delta.940 Up.Delta.941 Up.Delta.942 Up.Delta.943 Up.Delta.944 Up.Delta.945 Up.Delta.946 Up.Delta.947 Up.Delta.948 Up.Delta.949 Up.Delta.95 Up.Delta.950 Up.Delta.951 Up.Delta.952 Up.Delta.953 Up.Delta.954 Up.Delta.955 Up.Delta.956 Up.Delta.957 Up.Delta.958 Up.Delta.959 Up.Delta.96 Up.Delta.960 Up.Delta.961 Up.Delta.962 Up.Delta.963 Up.Delta.964 Up.Delta.965 Up.Delta.966 Up.Delta.967 Up.Delta.968 Up.Delta.969 Up.Delta.97 Up.Delta.970 Up.Delta.971 Up.Delta.972 Up.Delta.973 Up.Delta.974 Up.Delta.975 Up.Delta.976 Up.Delta.977 Up.Delta.978 Up.Delta.979 Up.Delta.98 Up.Delta.980 Up.Delta.981 Up.Delta.982 Up.Delta.983 Up.Delta.984 Up.Delta.985 Up.Delta.986 Up.Delta.987 Up.Delta.988 Up.Delta.989 Up.Delta.99 Up.Delta.990 Up.Delta.991 Up.Delta.992 Up.Delta.993 Up.Delta.994 Up.Delta.995 Up.Delta.996 Up.Delta.997 Up.Delta.998
**sample**	2	Up Down
**pos**	6	1 2 3 4 5 6
**base**	4	A C G U

**Number of Observations Read**	11988
**Number of Observations Used**	11988

Response Profile

**Ordered**	**Total**

Value	base	Frequency
1	A	2327
2	C	1453
3	G	3692
4	U	4516

In modeling category probabilities, base='A' serves as the reference category.

Dimensions	
G-side Cov. Parameters	3
Columns in X	63
Columns in Z per Subject	3
Subjects (Blocks in V)	1998
Max Obs per Subject	6

Optimization Information	
Optimization Technique	Dual Quasi-Newton
Parameters in Optimization	39
Lower Boundaries	3
Upper Boundaries	0
Fixed Effects	Not Profiled
Starting From	GLM estimates

Iteration History					
**Iteration**	**Restarts**	**Evaluations**	**Objective Function**	**Change**	**Max Gradient**
**0**	**0**	4	21783.176573	.	1102.086
**1**	**0**	2	21123.365173	659.81139944	423.161
**2**	**0**	3	21122.306221	1.05895253	417.6768
**3**	**0**	4	21014.275125	108.03109540	260.8271
**4**	**0**	2	20968.685652	45.58947316	227.9763
**5**	**0**	2	20954.785929	13.89972266	69.56161
**6**	**0**	2	20951.020615	3.76531481	24.43601
**7**	**0**	3	20950.103258	0.91735640	38.05331
**8**	**0**	3	20949.498121	0.60513755	12.10819
**9**	**0**	3	20949.156716	0.34140464	8.945594
**10**	**0**	3	20949.056246	0.10047026	8.130956
**11**	**0**	3	20948.99494	0.06130605	4.947842
**12**	**0**	2	20948.974477	0.02046313	9.782639
**13**	**0**	4	20948.918067	0.05640952	2.822133
**14**	**0**	4	20948.784316	0.13375057	3.830968
**15**	**0**	2	20948.702941	0.08137551	5.603241
**16**	**0**	2	20948.623834	0.07910672	2.422144
**17**	**0**	3	20948.594599	0.02923494	1.8497
**18**	**0**	2	20948.556795	0.03780390	2.236341
**19**	**0**	2	20948.50031	0.05648498	1.753916

**20**	**0**	3	20948.466767	0.03354318	2.413428
**21**	**0**	2	20948.411885	0.05488224	1.338206
**22**	**0**	3	20948.399861	0.01202395	1.224934
**23**	**0**	2	20948.386503	0.01335831	2.024217
**24**	**0**	2	20948.371293	0.01520938	1.858681
**25**	**0**	2	20948.34717	0.02412377	1.107685
**26**	**0**	3	20948.330785	0.01638486	1.248665
**27**	**0**	2	20948.325155	0.00562993	2.218039
**28**	**0**	4	20948.306201	0.01895357	0.815453
**29**	**0**	2	20948.28558	0.02062088	0.655605
**30**	**0**	3	20948.282629	0.00295102	0.505051
**31**	**0**	4	20948.267217	0.01541252	1.222814
**32**	**0**	2	20948.245674	0.02154276	1.066225
**33**	**0**	3	20948.236967	0.00870724	0.805149
**34**	**0**	3	20948.233631	0.00333622	0.665553
**35**	**0**	4	20948.220206	0.01342478	0.421357
**36**	**0**	3	20948.218906	0.00129997	0.373942
**37**	**0**	4	20948.210212	0.00869384	0.400355
**38**	**0**	3	20948.209409	0.00080295	0.44697
**39**	**0**	4	20948.205	0.00440918	0.467956
**40**	**0**	2	20948.199508	0.00549158	0.630752
**41**	**0**	3	20948.198253	0.00125532	0.465919
**42**	**0**	4	20948.193008	0.00524492	0.355395

**43**	**0**	3	20948.190766	0.00224201	0.212836
**44**	**0**	3	20948.190054	0.00071246	0.301902
**45**	**0**	4	20948.183961	0.00609257	0.646081
**46**	**0**	3	20948.181942	0.00201847	0.29453
**47**	**0**	2	20948.18027	0.00167221	0.454104
**48**	**0**	2	20948.17795	0.00232074	0.320849
**49**	**0**	4	20948.16649	0.01145993	0.363537
**50**	**0**	3	20948.164308	0.00218162	0.436886
**51**	**0**	4	20948.15881	0.00549836	0.327065
**52**	**0**	3	20948.157981	0.00082838	0.331947
**53**	**0**	4	20948.154048	0.00393307	0.541978
**54**	**0**	2	20948.152457	0.00159141	0.648352
**55**	**0**	2	20948.150189	0.00226768	0.245159
**56**	**0**	3	20948.149004	0.00118487	0.332197
**57**	**0**	2	20948.148032	0.00097214	0.349204
**58**	**0**	2	20948.146727	0.00130519	0.198074
**59**	**0**	2	20948.145236	0.00149053	0.298491
**60**	**0**	3	20948.144638	0.00059810	0.24206
**61**	**0**	4	20948.134922	0.00971593	0.318445
**62**	**0**	3	20948.133071	0.00185107	0.490788
**63**	**0**	2	20948.131935	0.00113636	0.514495
**64**	**0**	4	20948.129102	0.00283298	0.430228
**65**	**0**	4	20948.114718	0.01438411	0.19267

**66**	**0**	3	20948.114436	0.00028205	0.210218

Convergence criterion (GCONV=1E-8) satisfied.

Estimated G matrix is not positive definite.

Fit Statistics	
**-2 Log Likelihood**	20948.11
**AIC (smaller is better)**	21020.11
**AICC (smaller is better)**	21020.34
**BIC (smaller is better)**	21221.71
**CAIC (smaller is better)**	21257.71
**HQIC (smaller is better)**	21094.14

Fit Statistics for Conditional Distribution	
**-2 log L(base | r. effects)**	20948.11

Covariance Parameter Estimates

Cov Parm	Subject	Group	Estimate	Standard Error
**CHOL(1,1)**	**seedid**	**base C**	0	.
**CHOL(1,1)**	**seedid**	**base G**	0	.
**CHOL(1,1)**	**seedid**	**base U**	0	.

Solutions for Fixed Effects

Effect	base	sample	pos	Estimate	Standard Error	DF	t Value	Pr > |t|
**Intercept**	**C**			1.4478	0.3503	1977	4.13	<.0001
**Intercept**	**G**			3.9195	0.3183	1977	12.31	<.0001
**Intercept**	**U**			3.7778	0.3188	1977	11.85	<.0001
**sample**	**C**	**Up**		2.9104	0.4887	1977	5.96	<.0001
**sample**	**G**	**Up**		-1.2101	0.4729	1977	-2.56	0.0106
**sample**	**U**	**Up**		-0.7666	0.4711	1977	-1.63	0.1038
**sample**	**C**	**Down**		0	.	.	.	.
**sample**	**G**	**Down**		0	.	.	.	.
**sample**	**U**	**Down**		0	.	.	.	.
**pos**	**C**		**1**	-4.5954	0.4576	1977	-10.04	<.0001
**pos**	**G**		**1**	-3.1089	0.3263	1977	-9.53	<.0001
**pos**	**U**		**1**	-5.0575	0.3436	1977	-14.72	<.0001
**pos**	**C**		**2**	-5.7400	0.4619	1977	-12.43	<.0001
**pos**	**G**		**2**	-5.7511	0.3319	1977	-17.33	<.0001
**pos**	**U**		**2**	-6.7973	0.3576	1977	-19.01	<.0001
**pos**	**C**		**3**	-3.5142	0.3746	1977	-9.38	<.0001
**pos**	**G**		**3**	-7.5800	0.4246	1977	-17.85	<.0001
**pos**	**U**		**3**	-3.9667	0.3256	1977	-12.18	<.0001
**pos**	**C**		**4**	-3.4175	0.4985	1977	-6.86	<.0001
**pos**	**G**		**4**	-3.5390	0.3567	1977	-9.92	<.0001

**pos**	**U**		**4**	-1.2282	0.3438	1977	-3.57	0.0004
**pos**	**C**		**5**	-0.5309	0.4785	1977	-1.11	0.2674
**pos**	**G**		**5**	-0.5415	0.4241	1977	-1.28	0.2018
**pos**	**U**		**5**	-0.01360	0.4235	1977	-0.03	0.9744
**pos**	**C**		**6**	0	.	.	.	.
**pos**	**G**		**6**	0	.	.	.	.
**pos**	**U**		**6**	0	.	.	.	.
**sample*pos**	**C**	**Up**	**1**	-2.9586	0.6192	5979	-4.78	<.0001
**sample*pos**	**G**	**Up**	**1**	0.3264	0.4832	5979	0.68	0.4994
**sample*pos**	**U**	**Up**	**1**	0.8524	0.4982	5979	1.71	0.0871
**sample*pos**	**C**	**Up**	**2**	2.7748	0.6129	5979	4.53	<.0001
**sample*pos**	**G**	**Up**	**2**	6.3789	0.5204	5979	12.26	<.0001
**sample*pos**	**U**	**Up**	**2**	5.1038	0.5432	5979	9.40	<.0001
**sample*pos**	**C**	**Up**	**3**	0.09304	0.5432	5979	0.17	0.8640
**sample*pos**	**G**	**Up**	**3**	7.4060	0.5767	5979	12.84	<.0001
**sample*pos**	**U**	**Up**	**3**	3.3988	0.5064	5979	6.71	<.0001
**sample*pos**	**C**	**Up**	**4**	0.5769	0.6376	5979	0.90	0.3656
**sample*pos**	**G**	**Up**	**4**	2.1113	0.5417	5979	3.90	<.0001
**sample*pos**	**U**	**Up**	**4**	1.4391	0.5237	5979	2.75	0.0060
**sample*pos**	**C**	**Up**	**5**	-2.8778	0.5997	5979	-4.80	<.0001
**sample*pos**	**G**	**Up**	**5**	-2.4834	0.5717	5979	-4.34	<.0001
**sample*pos**	**U**	**Up**	**5**	-1.1916	0.5584	5979	-2.13	0.0329
**sample*pos**	**C**	**Up**	**6**	0	.	.	.	.

**sample*pos**	**G**	**Up**	**6**	0	.	.	.	.
**sample*pos**	**U**	**Up**	**6**	0	.	.	.	.
**sample*pos**	**C**	**Down**	**1**	0	.	.	.	.
**sample*pos**	**G**	**Down**	**1**	0	.	.	.	.
**sample*pos**	**U**	**Down**	**1**	0	.	.	.	.
**sample*pos**	**C**	**Down**	**2**	0	.	.	.	.
**sample*pos**	**G**	**Down**	**2**	0	.	.	.	.
**sample*pos**	**U**	**Down**	**2**	0	.	.	.	.
**sample*pos**	**C**	**Down**	**3**	0	.	.	.	.
**sample*pos**	**G**	**Down**	**3**	0	.	.	.	.
**sample*pos**	**U**	**Down**	**3**	0	.	.	.	.
**sample*pos**	**C**	**Down**	**4**	0	.	.	.	.
**sample*pos**	**G**	**Down**	**4**	0	.	.	.	.
**sample*pos**	**U**	**Down**	**4**	0	.	.	.	.
**sample*pos**	**C**	**Down**	**5**	0	.	.	.	.
**sample*pos**	**G**	**Down**	**5**	0	.	.	.	.
**sample*pos**	**U**	**Down**	**5**	0	.	.	.	.
**sample*pos**	**C**	**Down**	**6**	0	.	.	.	.
**sample*pos**	**G**	**Down**	**6**	0	.	.	.	.
**sample*pos**	**U**	**Down**	**6**	0	.	.	.	.

Odds Ratio Estimates

**Comparison**	**Estimate**	**DF**	**95% Confidence Limits**	

**C: sample Up vs Down**	12.328	1977	9.096	16.707
**G: sample Up vs Down**	2.944	1977	2.304	3.762
**U: sample Up vs Down**	2.302	1977	1.828	2.899
**C: pos 1 vs 6**	0.002	1977	0.001	0.004
**G: pos 1 vs 6**	0.053	1977	0.033	0.084
**U: pos 1 vs 6**	0.010	1977	0.006	0.016
**C: pos 2 vs 6**	0.013	1977	0.007	0.023
**G: pos 2 vs 6**	0.077	1977	0.046	0.129
**U: pos 2 vs 6**	0.014	1977	0.008	0.024
**C: pos 3 vs 6**	0.031	1977	0.018	0.053
**G: pos 3 vs 6**	0.021	1977	0.012	0.036
**U: pos 3 vs 6**	0.104	1977	0.063	0.170
**C: pos 4 vs 6**	0.044	1977	0.023	0.082
**G: pos 4 vs 6**	0.083	1977	0.049	0.142
**U: pos 4 vs 6**	0.601	1977	0.360	1.005
**C: pos 5 vs 6**	0.139	1977	0.077	0.251
**G: pos 5 vs 6**	0.168	1977	0.096	0.294
**U: pos 5 vs 6**	0.544	1977	0.314	0.940

Type III Tests of Fixed Effects

Effect	Num DF	Den DF	F Value	Pr > F
**sample**	3	1977	95.36	<.0001
**pos**	15	1977	179.83	<.0001

**sample*pos**	15	5979	93.07	<.0001

```
>
+ proc plm restore=gmxres noclprint plots=none;
+ lsmeans sample/ilink oddsratio adj=tukey cl e;
+ slice sample*pos/sliceby=pos diff oddsratio adj=tukey cl;
+ ods output slicediffs= sampleposdiffs diffs= samplediffs;
+ run;
```

```
> proc sort data=sampleposdiffs;
+ by sample slice base;
+
+ proc print data=sampleposdiffs;
+ var sample _sample slice base OddsRatio AdjLowerOR AdjUpperOR Adjp;
+ run;
```

Obs	sample	_sample	Slice	base	OddsRatio	AdjLowerOR	AdjUpperOR	Adjp
**1**	Up	Down	pos 1	C	0.953	0.452	2.008	0.8992
**2**	Up	Down	pos 1	G	0.413	0.340	0.502	<.0001
**3**	Up	Down	pos 1	U	1.090	0.793	1.496	0.5962
**4**	Up	Down	pos 2	C	294.486	142.625	608.046	<.0001
**5**	Up	Down	pos 2	G	175.698	114.791	268.922	<.0001
**6**	Up	Down	pos 2	U	76.492	45.017	129.976	<.0001
**7**	Up	Down	pos 3	C	20.156	12.663	32.083	<.0001
**8**	Up	Down	pos 3	G	490.730	256.967	937.149	<.0001
**9**	Up	Down	pos 3	U	13.903	9.659	20.012	<.0001
**10**	Up	Down	pos 4	C	32.699	14.652	72.970	<.0001
**11**	Up	Down	pos 4	G	2.462	1.467	4.133	0.0007
**12**	Up	Down	pos 4	U	1.959	1.251	3.067	0.0033

**13**	Up	Down	pos 5	C	1.033	0.523	2.042	0.9251
**14**	Up	Down	pos 5	G	0.025	0.013	0.047	<.0001
**15**	Up	Down	pos 5	U	0.141	0.078	0.254	<.0001
**16**	Up	Down	pos 6	C	18.365	7.045	47.872	<.0001
**17**	Up	Down	pos 6	G	0.298	0.118	0.753	0.0105
**18**	Up	Down	pos 6	U	0.465	0.184	1.170	0.1037

```
> * update the path in the OUTFILE statement below to match datapath;
+ * note that sas uses single backslash for paths, e.g. "C:\sasdata";
+
+ proc export data=work.sampleposdiffs
+   outfile="G:\PeterM_XXX\Analysis\Data\MethodsPaper\F_OREstimates_Fig3_081221
.xlsx"
+   dbms=excel replace;
+   sheet="OR Position";
+ run;
```

4 Up vs. Down

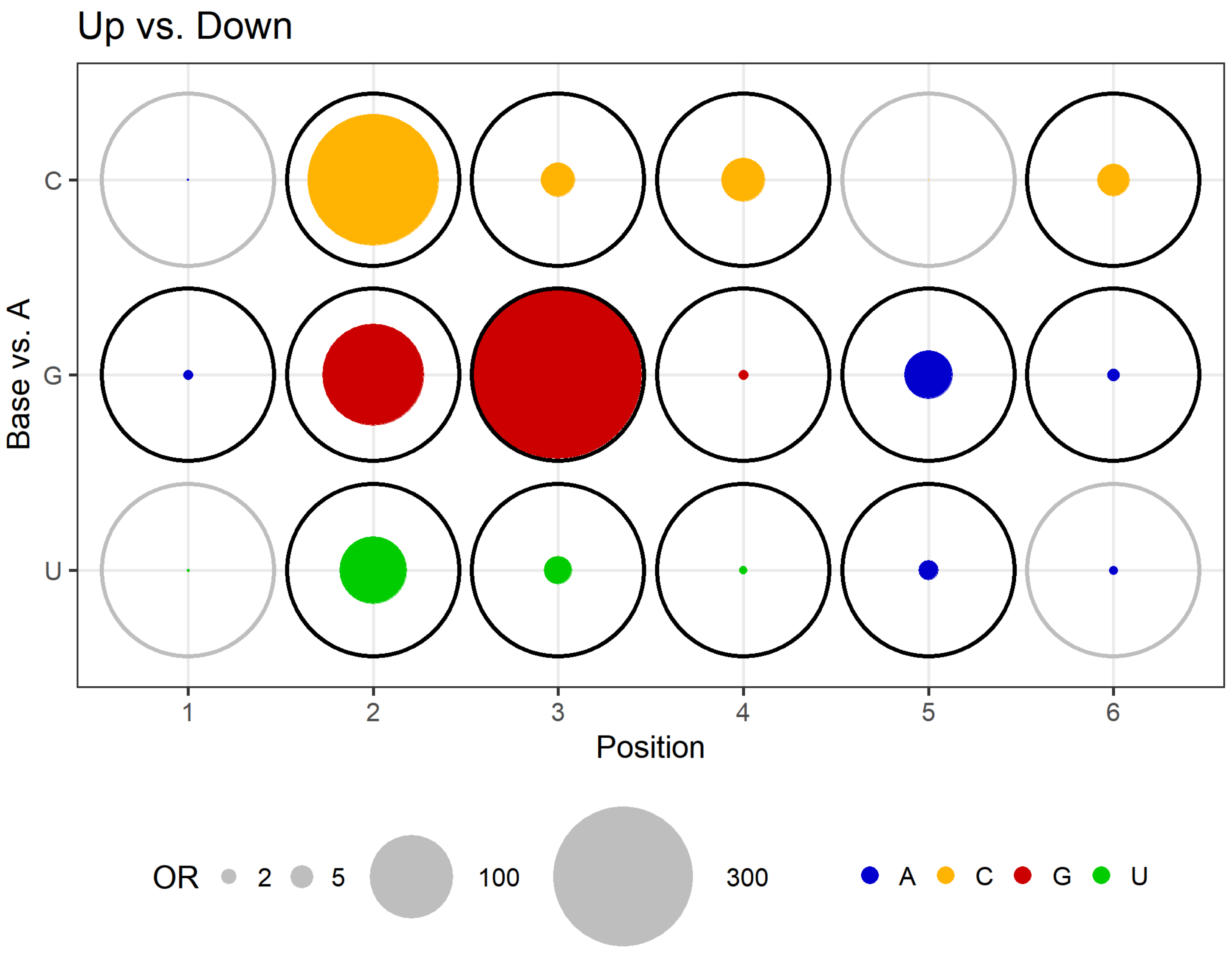
Up vs. Down
Base vs. A
C
G
U
1
2
3
4
5
6
Position
OR
2
5
100
300
A
C
G
U
